# Supplementary material for: Design and Evaluation of Indole-Based Schiff Bases as α-Glucosidase Inhibitors: CNN-Enhanced Docking, MD Simulations, ADMET Profiling, and SAR Analysis
Source: Molecules. 2025 Sep 8;30(17):3651. doi: 10.3390/molecules30173651 (PMC12430303; doi:10.3390/molecules30173651)
Supplement: Supplementary file 1 [file molecules-30-03651-s001.zip › molecules-3830332-supplementary.pdf]

Design and Evaluation of Indole-Based Schiff Bases as α-Glucosidase Inhibitors: CNN-Enhanced Docking, MD Simulations, ADMET Profiling, and SAR Analysis

Seema K. Bhagwat [1], Sachin V. Patil [1], Abraham Vidal-Limon [2], J. Oscar C. Jimenez-Halla [3], Balasaheb K. Ghotekar [1], Vivek D. Bobade, [1] Irving David Perez-Landa [4], Enrique Delgado-Alvarado [5], Fabiola Hernandez-Rosas [6,7*], and Tushar Janardan Pawar [2*]

1 Department of Chemistry, Research Centre HPT Arts and RYK Science College (Affiliated to S. P. Pune University), Nashik, 422005, Maharashtra, India; seemabhagwat777@gmail.com (S.K.B.); sachin.dhokare@gmail.com (S.V.P.); balaghotekar@gmail.com (B.K.G); v_bobade31@rediffmail.com (V.D.B.)
2 Red de Estudios Moleculares Avanzados, Instituto de Ecología, A. C., Carretera Antigua a Coatepec 351, 91073, Xalapa, Veracruz, México; abraham.vidal@inecol.mx (A.V.L.) tushar.janardan@inecol.mx (T.J.P.)
3 Departamento de Química, División de Ciencias Naturales y Exactas, Universidad de Guanajuato, Noria Alta S/N, 36050, Guanajuato, Guanajuato, México; jjimenez@ugto.mx (J.O.C.J.H.)
4 Tecnológico Nacional de México/Instituto Tecnológico de Boca del Río, Carretera Veracruz-Córdoba 12, Boca del Río, Veracruz, 94290, Mexico; irvingperez@bdelrio.tecnm.mx (I.D.P.L.)
5 Micro and Nanotechnology Research Center, Universidad Veracruzana, Blvd. Av. Ruiz Cortines No. 455 Fracc. Costa Verde, Boca del Río, Veracruz 94294, México; endelgado@uv.mx (E.D.A.)
6 Centro de Investigación, Universidad Anáhuac Querétaro, El Marqués, Querétaro 76246, México. fabiola.hernandezro@anahuac.mx (F.H.-R.)
7 Facultad de Química, Universidad Autónoma de Querétaro, Querétaro 76010, México.
* Correspondence: tushar.janardan@inecol.mx (T.J.P.); fabiola.hernandezro@anahuac.mx (F.H.-R.)

Table of Content

1. IC_{50} Plots — S2
2. DFT Study — S3
3. Molecular Dynamics Simulations — S18
4. ADMET Profiling — S22
5. Statistical Analysis and Visualizations — S28
6. SAR Analysis — S36
7. NMR Spectra — S49
8. Mass Spectra — S74
9. References — S85

S1. IC_{50} Plots

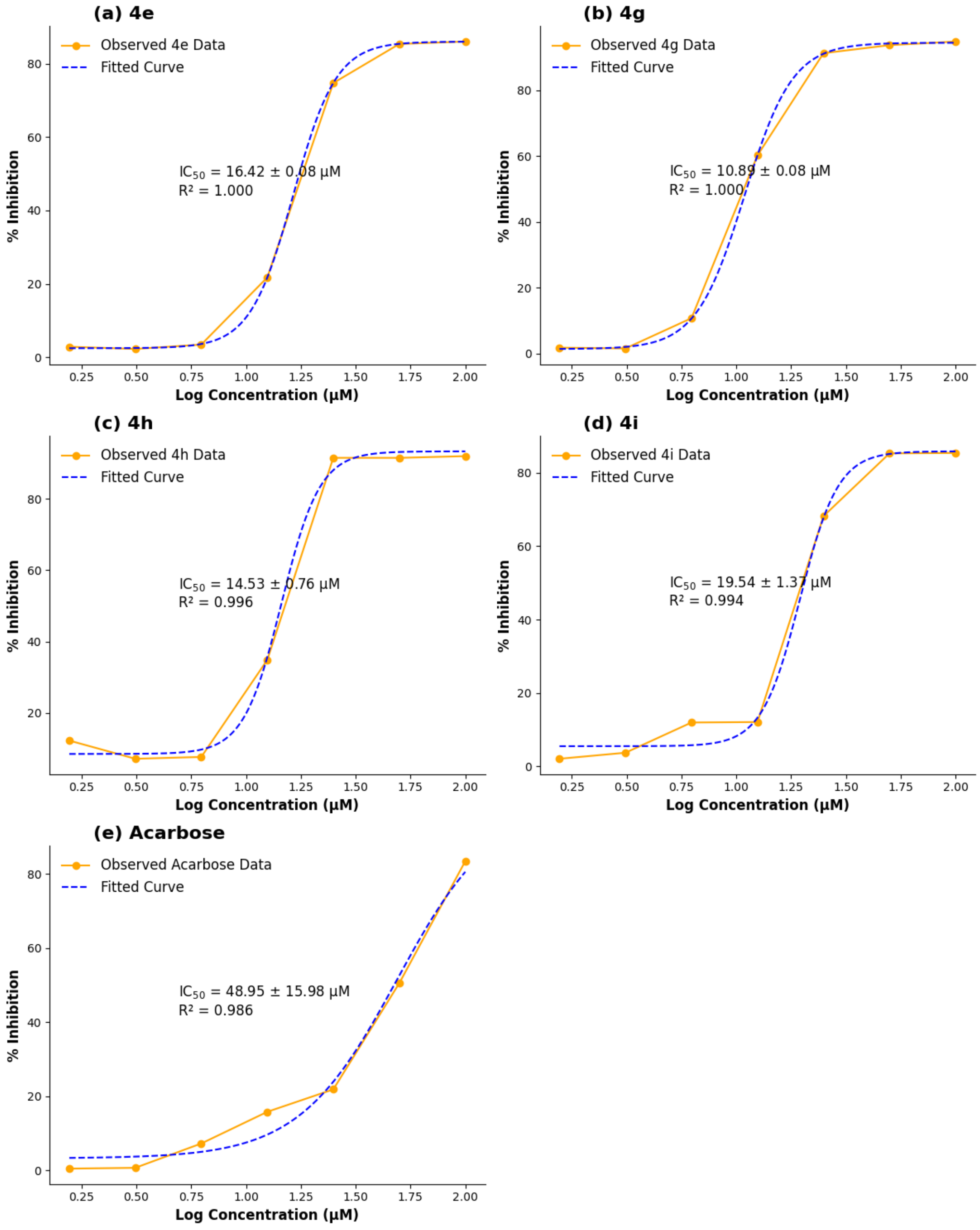

Figure S1. Dose-response curves for the *α*-glucosidase inhibition assay. Plots show the observed % inhibition versus the logarithm of concentration for the most active compounds: (a) **4e**, (b) **4g**, (c) **4h**, (d) **4i**, and the standard inhibitor (e) Acarbose. The calculated IC_{50} and R^2 values are shown for each curve..

S2. DFT Study

S2.1. Methodology

Theoretical calculations were performed using the Gaussian09 rev. D.01 package.[1] Gas-phase geometry optimizations were carried out (no constraints or symmetry restrictions were used) with the long-range hybrid functional ωB97X-D[2] in conjunction with Ahlrichs' def2-tzvpp basis set[3] for all the atoms. Subsequent harmonic frequency calculations were performed to corroborate the local minimum character of each optimized species (all the values are positive for all the compounds). We plotted the electrostatic potential of each compound using Gaussview program.[4] The band gap energy was calculated simply as the difference between the LUMO energy and the HOMO energy: $E_{gap} = E_{LUMO} - E_{HOMO}$ expressed in electron-volts (eV).

We also took into account different conformations for each **4a-j** derivative as follows:

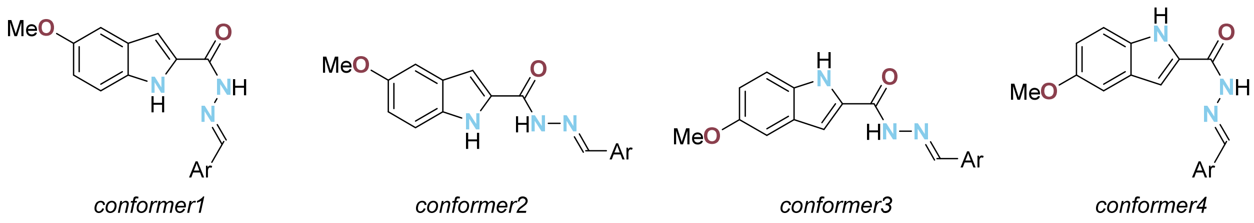

Figure S2a. Conformational analysis of **4a–4j**. Four conformers (1-4) were identified, with Conformer1 being the most stable for most derivatives due to its minimized steric repulsion and optimized intramolecular interactions.

We also tested the rotation over the C-Ar bond to find possible hydrogen bonds that could stabilize each compound. *Conformer1* was found to be the lowest-in-energy isomer (more than 5.0 kcal/mol than the rest of conformers) for all the cases but **4e** which has a different one (*conformer2*). Moreover, we also found that compounds **4a** and **4f** are planar (C_s symmetry) whereas the rest of compounds are not planar due to a strong H-H repulsion between the indole and the benzene

rings. Presumably, the thiazole (5-membered ring instead of benzene) in **4a** or the strong H--F interaction in **4f** induce the planarity in these cases.

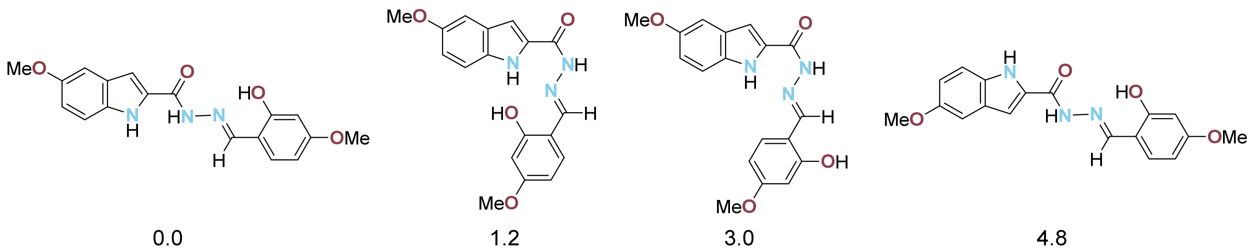

Figure S2b. Conformational energy profile for compound **4e**. The stability of different conformers is shown in Gibbs free energy (kcal/mol).

Tested conformations of **4e** (all local minima have symmetry C_1) with energy values expressed as Gibbs free energies in kcal/mol. Presumably, the lowest in energy has a strong hydrogen bond between the amidic nitrogen and the hydroxyl group (*conformer3*) which prevents the conversion to conformer4 as for the other compounds.

S2.2. HOMO-LUMO Gap Analysis

Table S1. Orbital energies of HOMO and LUMO in hartrees and energy gap (in eV) calculated at the ωB97X-D/def2-tzvpp level.

Compound	HOMO	LUMO	Energy gap
**4a**	–0.2693	–0.0218	6.74
**4b**	–0.2763	–0.0191	7.00
**4c**	–0.2707	–0.0034	7.28
**4d**	–0.2778	–0.0500	6.20
**4e**	–0.2843	–0.0011	7.71
**4f**	–0.2684	–0.0125	6.96
**4g**	–0.2755	–0.0179	7.01
**4h**	–0.2795	–0.0505	6.23
**4i**	–0.2722	–0.0154	6.99
**4j**	–0.2725	–0.0100	7.14

S2.3. Molecular Electrostatic Potential (MEP) Analysis

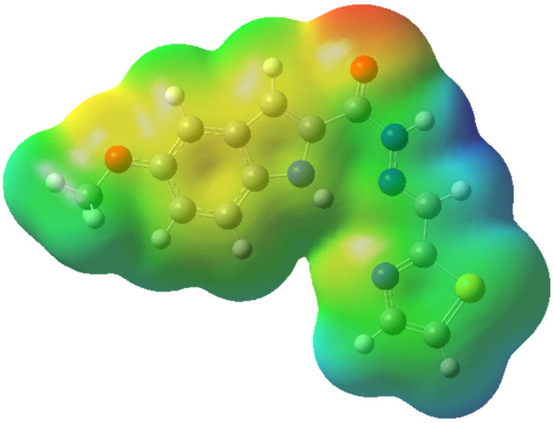	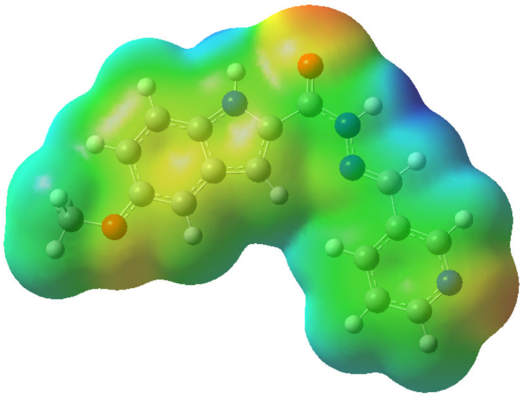
**4a**	**4b**

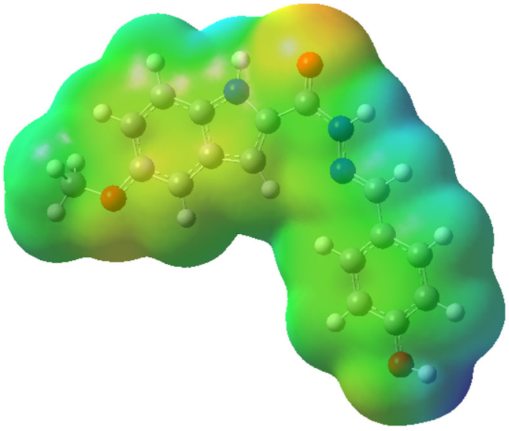

4c

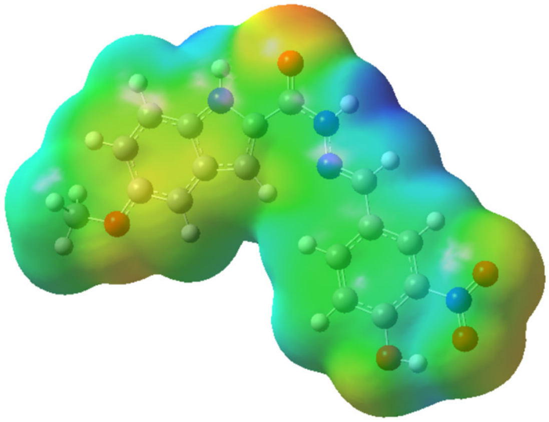

4d

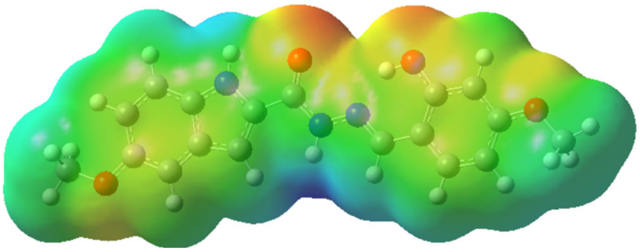

4e

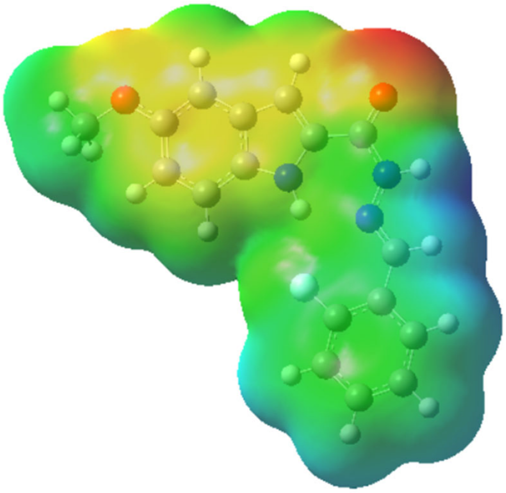

4f

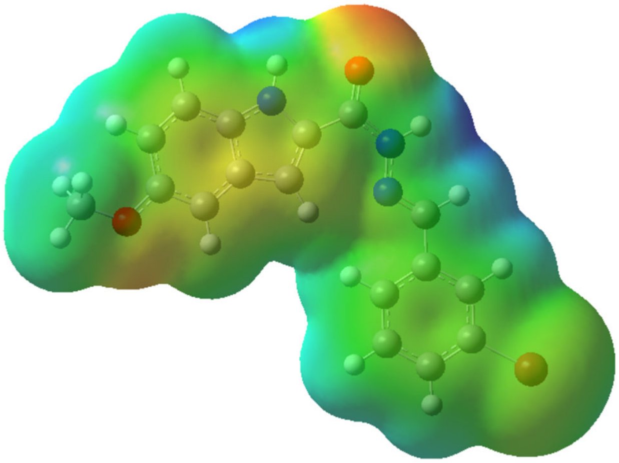 4g	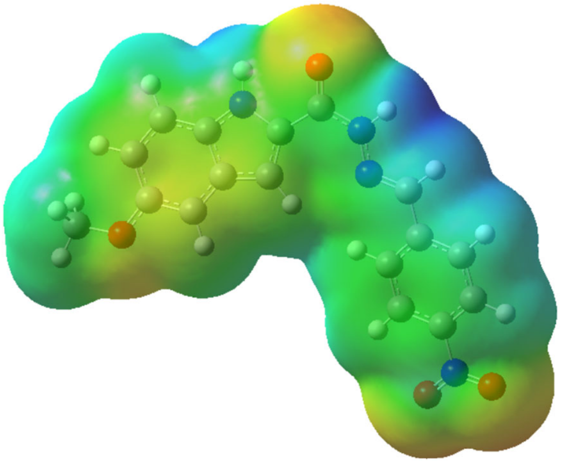 4h

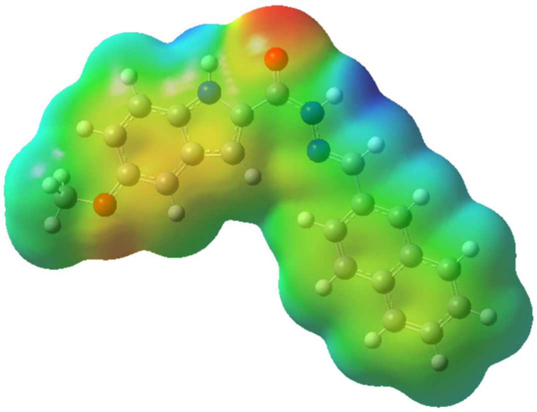 4i	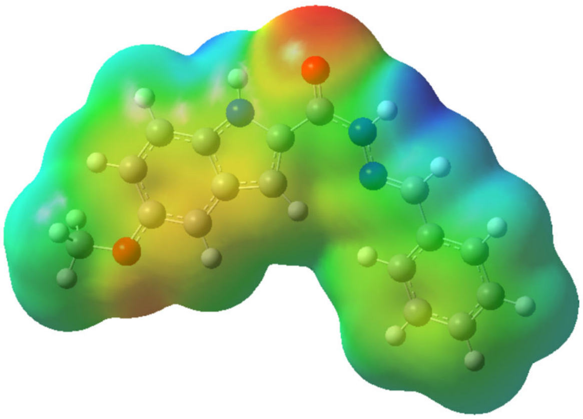 4j

Molecular Electrostatic Potential (MEP) maps were generated by mapping the charge distribution onto the electron density. These maps illustrate the partial distribution of charge across the molecule's surface. For compounds **4a**-**b**, **4f**-**g**, and **4i**-**j**, the carboxylic oxygen was identified as the most nucleophilic site, while the hydrazine protons were determined to be the most acidic. In contrast, compounds **4c**-**d** and **4h** exhibited a more distributed charge, with an extended nucleophilic and electrophilic zone. Compound **4e**, due to its distinct conformation, displayed the carboxylic oxygen as the most nucleophilic site and the indole proton as the most electrophilic site.

These results indicate that, although all these compounds are indole-based Schiff base derivatives, their reactivity patterns can be classified into three distinct groups depending on the substituents.

Table S2. Cartesian coordinates (xyz) of the geometry optimizations for compounds 4a-j calculated at the ωB97X-D/def2-tzvpp level.

4a

E(scf) = -1308.37146145 a.u.

N	-1.058309	-2.182599	0.000000
C	-0.686299	-3.397178	0.000000
H	-1.391333	-4.229739	0.000000
N	-2.354102	-1.875293	0.000000
H	-3.069418	-2.593620	0.000000
C	-2.835108	-0.571334	0.000000
O	-4.035973	-0.415867	0.000000
C	0.740910	-3.694300	0.000000
C	2.911825	-3.398500	0.000000
C	2.910709	-4.758298	0.000000
H	3.800200	-2.784786	0.000000
H	3.752592	-5.429011	0.000000
N	1.688980	-2.802692	0.000000
S	1.303934	-5.325620	0.000000
C	-1.891474	0.557778	0.000000
C	-2.284227	1.871567	0.000000
C	-1.101198	2.659802	0.000000
H	-3.306839	2.206483	0.000000
C	-0.015842	1.757171	0.000000
H	0.000000	-0.375056	0.000000
C	-0.856452	4.036518	0.000000
C	1.300802	2.207058	0.000000
C	0.448009	4.481271	0.000000
H	-1.663199	4.756399	0.000000
C	1.522204	3.567176	0.000000
H	2.131487	1.514298	0.000000
H	2.540667	3.925399	0.000000
N	-0.517152	0.488194	0.000000
O	0.620876	5.832706	0.000000
C	1.927823	6.345357	0.000000
H	1.829231	7.428109	0.000000
H	2.484217	6.039466	0.891425
H	2.484217	6.039466	-0.891425

4b

E(scf) = -987.593433257 a.u.

N	-2.192501	1.163086	-0.013930
C	-3.454396	1.017363	-0.053038
H	-4.125036	1.878313	-0.130341
N	-1.708689	2.417664	-0.055687
H	-2.337859	3.212091	-0.071457
C	-0.379222	2.749807	-0.012607
O	-0.073786	3.928915	0.012910
C	-4.076263	-0.303672	0.000516
C	-3.351258	-1.476438	0.199321
C	-5.455871	-0.416068	-0.148285
C	-4.027794	-2.677703	0.229656
H	-2.279280	-1.432112	0.333459
H	-6.056578	0.476031	-0.300292
C	-5.407610	-2.673115	0.064733
H	-3.501786	-3.609694	0.382437
H	-5.965746	-3.602285	0.085195
N	-6.116503	-1.566285	-0.119677
C	0.657494	1.705677	-0.006207
C	0.671598	0.338531	-0.110635
C	2.037596	-0.064885	-0.064439
H	-0.190994	-0.290634	-0.214399
C	2.807349	1.110465	0.069195
H	2.161666	3.145750	0.164636
C	2.675294	-1.306801	-0.123688
C	4.195214	1.062448	0.144548
C	4.051365	-1.354180	-0.049516
H	2.117723	-2.227538	-0.226459
C	4.806800	-0.171504	0.084069
H	4.786984	1.962049	0.247249
H	5.883840	-0.218708	0.141194
N	1.945340	2.167523	0.102151
O	4.612412	-2.593053	-0.112847
C	6.010910	-2.704199	-0.043655
H	6.234664	-3.765797	-0.112821
H	6.500652	-2.183085	-0.871904
H	6.401981	-2.319552	0.903196

4c

E(scf) = -1046.79341026 a.u.

N	-1.726821	1.548526	-0.006842
C	-2.997390	1.548929	-0.043271
H	-3.558361	2.487410	-0.103265
N	-1.108546	2.748336	-0.031760
H	-1.650208	3.604757	-0.040921
C	0.244908	2.937047	0.000426
O	0.678983	4.076979	0.021932
C	-3.782394	0.318438	-0.010061
C	-5.165343	0.388765	-0.135983
C	-3.192394	-0.938579	0.146553
C	-5.945719	-0.754920	-0.114847
H	-5.644755	1.352848	-0.255384
H	-2.119262	-1.007316	0.256861
C	-5.342329	-1.995975	0.037056
C	-3.957868	-2.082750	0.169438
H	-3.504064	-3.056254	0.292196
H	-7.022026	-0.680766	-0.215944
O	-6.044581	-3.152753	0.066950
H	-6.979197	-2.971091	-0.031004
C	1.165926	1.787868	0.001820
C	1.034518	0.425497	-0.075856
C	2.351792	-0.118297	-0.045285
H	0.108364	-0.110500	-0.150229
C	3.243102	0.971884	0.051822
H	2.813284	3.065317	0.125187
C	2.854962	-1.420906	-0.091187
C	4.619262	0.779549	0.103299
C	4.219848	-1.611382	-0.040400
H	2.201942	-2.279580	-0.165487
C	5.097532	-0.512933	0.056288
H	5.303907	1.613736	0.177707
H	6.164711	-0.671494	0.095059
N	2.496875	2.113810	0.078879
O	4.646404	-2.904162	-0.089164
C	6.026055	-3.160594	-0.039842
H	6.136029	-4.241077	-0.089605
H	6.553157	-2.710558	-0.886746
H	6.472688	-2.800491	0.892070

4d

E(scf) = -1251.32165660 a.u.

N	0.753223	1.699635	0.029601
C	2.022740	1.739339	-0.003833
H	2.563097	2.686392	-0.090420
N	0.089450	2.868540	-0.027021
H	0.593761	3.747532	-0.036554
C	-1.275010	2.999431	0.014382
O	-1.752159	4.119651	0.047764
C	2.827731	0.521844	0.069735
C	4.194910	0.599551	-0.080192
C	2.251418	-0.740478	0.293866
C	4.981851	-0.547786	-0.020444
H	4.686797	1.546733	-0.249845
H	1.181033	-0.809498	0.429244
C	4.410813	-1.811435	0.195204
C	3.018974	-1.872291	0.352914
H	2.576902	-2.842955	0.527115
N	6.413168	-0.396436	-0.187206
O	7.110363	-1.410332	-0.137350
O	6.868800	0.705976	-0.367201
O	5.091585	-2.945829	0.264263
H	6.033221	-2.727099	0.137361
C	-2.144322	1.812434	0.006806
C	-1.955391	0.461465	-0.132900
C	-3.245411	-0.142545	-0.091270
H	-1.010112	-0.029686	-0.259063
C	-4.180606	0.900932	0.076146
H	-3.845839	3.007254	0.214444
C	-3.690924	-1.463982	-0.180027
C	-5.545138	0.644162	0.156310
C	-5.043860	-1.718365	-0.101309
H	-3.003025	-2.288198	-0.309288
C	-5.966111	-0.665342	0.066222
H	-6.263790	1.442253	0.285071
H	-7.023580	-0.874115	0.126555
N	-3.485663	2.073822	0.132925
O	-5.414103	-3.024918	-0.194505
C	-6.779737	-3.346066	-0.121832
H	-6.842548	-4.427075	-0.218014

H	-7.215781	-3.048680	0.836694
H	-7.348839	-2.883380	-0.933835

4e

E(scf) = -1161.33816444 a.u.

N	-1.593534	0.221767	0.000179
C	-2.423497	-0.747550	0.000390
H	-2.080423	-1.787039	0.000597
N	-0.270287	-0.057217	0.000247
H	0.037313	-1.018379	-0.000033
C	0.647044	0.956754	0.000178
O	0.351104	2.131656	0.000274
C	-3.852660	-0.525633	0.000227
C	-4.716008	-1.618713	0.000324
C	-4.416410	0.772807	0.000000
C	-6.088211	-1.478922	0.000210
H	-4.290727	-2.615463	0.000515
C	-6.626739	-0.188981	-0.000031
C	-5.794011	0.921271	-0.000136
H	-6.221217	1.913490	-0.000327
H	-6.716838	-2.355174	0.000334
O	-3.672469	1.879615	-0.000123
H	-2.726852	1.639123	-0.000101
O	-7.949092	0.073984	-0.000143
C	-8.853070	-1.006589	-0.000521
H	-9.847123	-0.567909	-0.000947
H	-8.733785	-1.627320	-0.892394
H	-8.734554	-1.627308	0.891465
C	2.056747	0.515590	-0.000037
C	2.662698	-0.713561	-0.000095
C	4.070305	-0.479812	-0.000124
H	2.180724	-1.677334	-0.000206
C	4.248098	0.920364	-0.000117
H	2.772041	2.471921	0.000111
C	5.186581	-1.318636	-0.000118
C	5.515839	1.489186	-0.000055
C	6.445393	-0.753538	-0.000081
H	5.089491	-2.395529	-0.000181
C	6.607156	0.646098	-0.000042
H	5.653192	2.562047	-0.000037

4f

E(scf) = -1070.80962134 a.u.

N	0.297654	2.369471	0.000000
C	-0.428705	3.413294	0.000000
H	0.025482	4.408279	0.000000
N	1.630473	2.526515	0.000000
H	2.041777	3.452993	0.000000
C	2.552794	1.493133	0.000000
O	3.728901	1.787181	0.000000
C	-1.889304	3.390817	0.000000
C	-2.565511	4.613610	0.000000
C	-2.679924	2.242166	0.000000
C	-3.945825	4.686828	0.000000
H	-1.981250	5.525387	0.000000
C	-4.695067	3.520549	0.000000
C	-4.057866	2.290622	0.000000
H	-4.612889	1.363183	0.000000
H	-4.435024	5.650642	0.000000
H	-5.775462	3.563326	0.000000
F	-2.108452	1.030304	0.000000
C	2.094033	0.095288	0.000000
C	2.934044	-0.987573	0.000000
C	2.113416	-2.149242	0.000000
H	4.008604	-0.931575	0.000000
C	0.775338	-1.698335	0.000000
H	0.000000	0.289873	0.000000
C	2.380107	-3.521635	0.000000
C	-0.290943	-2.591650	0.000000
C	1.322989	-4.406276	0.000000
H	3.391819	-3.903191	0.000000
C	-0.008116	-3.940729	0.000000
H	-1.315508	-2.244754	0.000000
H	-0.829061	-4.641919	0.000000
N	0.788262	-0.333565	0.000000
O	1.648831	-5.729422	0.000000
C	0.614325	-6.678258	0.000000
H	1.096234	-7.652885	0.000000

H	7.595872	1.079288	0.000033
N	3.007159	1.495597	-0.000054
O	7.490592	-1.624394	-0.000122
C	8.798009	-1.109730	-0.000044
H	9.462347	-1.970144	-0.000122
H	8.996278	-0.507212	-0.891579
H	8.996231	-0.507395	0.891627

H	-0.014967	-6.593393	0.891450
H	-0.014967	-6.593393	-0.891450

4g

E(scf) = -3545.17145794 a.u.

N	-0.569355	1.574471	0.057788
C	-1.839712	1.577159	0.039824
H	-2.404963	2.509471	-0.050328
N	0.058462	2.760981	-0.019579
H	-0.471590	3.624678	-0.038856
C	1.419068	2.931429	-0.008185
O	1.864273	4.065395	-0.007554
C	-2.613334	0.337417	0.134152
C	-3.994346	0.400745	-0.026910
C	-2.008748	-0.894461	0.382080
C	-4.749237	-0.756951	0.050595
H	-4.477812	1.350015	-0.215528
H	-0.938605	-0.940612	0.523750
C	-4.155380	-1.982990	0.289713
C	-2.777333	-2.039395	0.456128
H	-2.305690	-2.993308	0.649697
H	-4.755833	-2.879197	0.349230
Br	-6.626832	-0.654154	-0.174728
C	2.322011	1.769809	-0.007449
C	2.168539	0.410220	-0.098514
C	3.476485	-0.155040	-0.074253
H	1.234584	-0.111253	-0.180333
C	4.385256	0.919398	0.033003
H	3.992737	3.018569	0.118163
C	3.957961	-1.465466	-0.133530
C	5.758379	0.704253	0.081894
C	5.319420	-1.678549	-0.085636
H	3.290960	-2.312498	-0.216409
C	6.214916	-0.595109	0.021540
H	6.456606	1.526281	0.164314
H	7.279274	-0.772157	0.057936

4h

E(scf) = -1176.07947548 a.u.

N	0.953467	-2.051722	-0.018317
C	2.212244	-2.219042	-0.078564
H	2.651623	-3.216013	-0.172170
N	0.172866	-3.140500	-0.053496
H	0.580197	-4.068696	-0.067586
C	-1.200498	-3.125906	0.014839
O	-1.787321	-4.190743	0.069139
C	3.135112	-1.084009	-0.031102
C	4.493356	-1.308632	-0.245180
C	2.690322	0.214049	0.226925
C	5.397786	-0.262870	-0.215648
H	4.847959	-2.312412	-0.441061
H	1.640257	0.388125	0.411825
C	4.925371	1.010750	0.034592
C	3.581961	1.265569	0.258727
H	3.255347	2.274747	0.458032
H	6.452078	-0.421671	-0.382471
N	5.879931	2.130525	0.069384
O	5.438944	3.236767	0.292866
O	7.048803	1.877823	-0.128015
C	-1.938433	-1.855146	0.009981
C	-1.608663	-0.533495	-0.150810
C	-2.825261	0.205571	-0.091736
H	-0.619274	-0.147076	-0.301999
C	-3.863132	-0.729580	0.107258
H	-3.755417	-2.857283	0.266311
C	-3.127948	1.566435	-0.188890
C	-5.190516	-0.327028	0.210881
C	-4.443803	1.965272	-0.087067
H	-2.358389	2.310409	-0.341910
C	-5.470021	1.018904	0.112171

N	3.658227	2.073294	0.072027
O	5.725238	-2.976721	-0.147586
C	7.101100	-3.255769	-0.103723
H	7.193462	-4.337188	-0.165816
H	7.633547	-2.804752	-0.946631
H	7.554749	-2.913463	0.831403

H	-5.987656	-1.041936	0.363929
H	-6.497316	1.341461	0.190532
N	-3.297413	-1.969847	0.165318
O	-4.674161	3.302291	-0.189906
C	-5.995895	3.769159	-0.095647
H	-5.944156	4.849443	-0.205081
H	-6.627561	3.361271	-0.890556
H	-6.441657	3.531316	0.874921

4i

E(scf) = -1125.19934590 a.u.

N	-0.781087	1.826692	0.007179
C	-2.047014	1.940744	-0.018462
H	-2.525230	2.923714	-0.073233
N	-0.058066	2.963283	-0.025426
H	-0.519304	3.865670	-0.035444
C	1.309012	3.028387	-0.000384
O	1.842262	4.124708	0.015461
C	-2.932699	0.779813	0.020721
C	-4.286788	0.968676	-0.073288
C	-2.421156	-0.536671	0.155394
C	-5.186562	-0.121131	-0.045328
H	-4.687935	1.970993	-0.173971
C	-3.263558	-1.603107	0.185428
H	-1.353071	-0.675768	0.240019
C	-6.585925	0.057113	-0.143660
C	-4.667982	-1.432335	0.085092
H	-2.867348	-2.605427	0.290108
C	-7.428407	-1.017175	-0.113824
H	-6.980258	1.060774	-0.243501
C	-5.564233	-2.523554	0.113422
C	-6.912001	-2.321805	0.016133
H	-8.497511	-0.870428	-0.189969
H	-5.165372	-3.525281	0.213611
H	-7.589487	-3.164924	0.038535
C	2.121825	1.801216	0.000276
C	1.866461	0.456316	-0.074068
C	3.128650	-0.205106	-0.047804
H	0.895105	0.006616	-0.143419
C	4.115949	0.799372	0.043697
H	3.880103	2.923197	0.113506

4j

E(scf) = -971.557635123 a.u.

N	-2.183341	1.174151	-0.007142
C	-3.445792	1.035319	-0.047505
H	-4.107416	1.904043	-0.122932
N	-1.695660	2.429332	-0.044753
H	-2.323210	3.225048	-0.058669
C	-0.366995	2.757248	-0.006557
O	-0.055116	3.935605	0.016575
C	-4.086649	-0.279497	0.000049
C	-5.466902	-0.362637	-0.162538
C	-3.356298	-1.450368	0.206078
C	-6.108693	-1.590650	-0.129524
H	-6.042455	0.541770	-0.319101
C	-5.375285	-2.748670	0.069893
C	-3.997799	-2.673607	0.238827
H	-3.423438	-3.576034	0.400445
H	-7.181393	-1.641594	-0.259207
H	-5.873188	-3.708664	0.097489
H	-2.286423	-1.388365	0.347296
C	0.666357	1.708983	-0.002403
C	0.674693	0.341297	-0.097226
C	2.039922	-0.066295	-0.058042
H	-0.191508	-0.284995	-0.188878
C	2.814863	1.107308	0.061664
H	2.175678	3.145321	0.150117
C	2.673079	-1.310610	-0.113335
C	4.202942	1.054976	0.126949
C	4.049578	-1.362213	-0.049077
H	2.111379	-2.229976	-0.205370
C	4.810122	-0.181612	0.070594
H	4.798540	1.953254	0.218885

C	3.510228	-1.548424	-0.093152
C	5.469014	0.481937	0.090466
C	4.852063	-1.862972	-0.047228
H	2.780950	-2.343671	-0.163604
C	5.826709	-0.848869	0.044150
H	6.227467	1.249946	0.161036
H	6.875203	-1.103474	0.079400
N	3.477357	2.004701	0.071489
O	5.158270	-3.189251	-0.095187
C	6.508817	-3.571537	-0.051578
H	6.518574	-4.657554	-0.101001
H	7.071293	-3.172243	-0.901130
H	6.990729	-3.253859	0.878156

H	5.887412	-0.232074	0.120053
N	1.956557	2.167413	0.093206
O	4.605997	-2.603953	-0.108080
C	6.004238	-2.719116	-0.047504
H	6.224314	-3.781927	-0.110482
H	6.490324	-2.205555	-0.882691
H	6.403156	-2.328872	0.893847

S3. Molecular Dynamics Simulation Methods

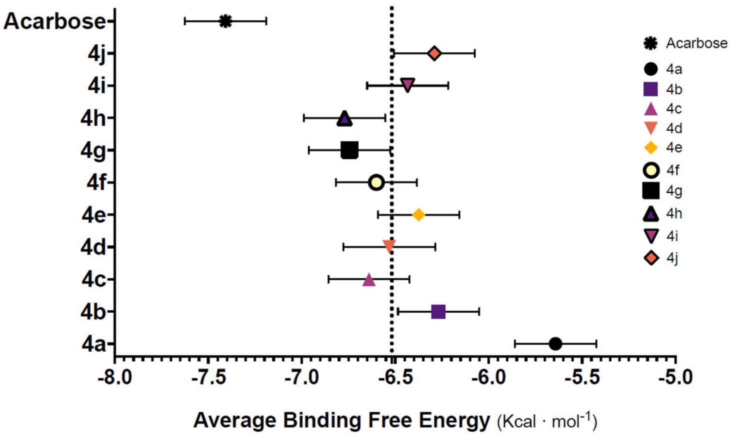

Figure S3. Plot of average binding free energies (BFE) for the indole-based Schiff base derivatives (**4a–4j**) and Acarbose. The values represent the average BFE calculated from docking interactions with 10 distinct conformations of the α-glucosidase enzyme, with error bars indicating the standard deviation.

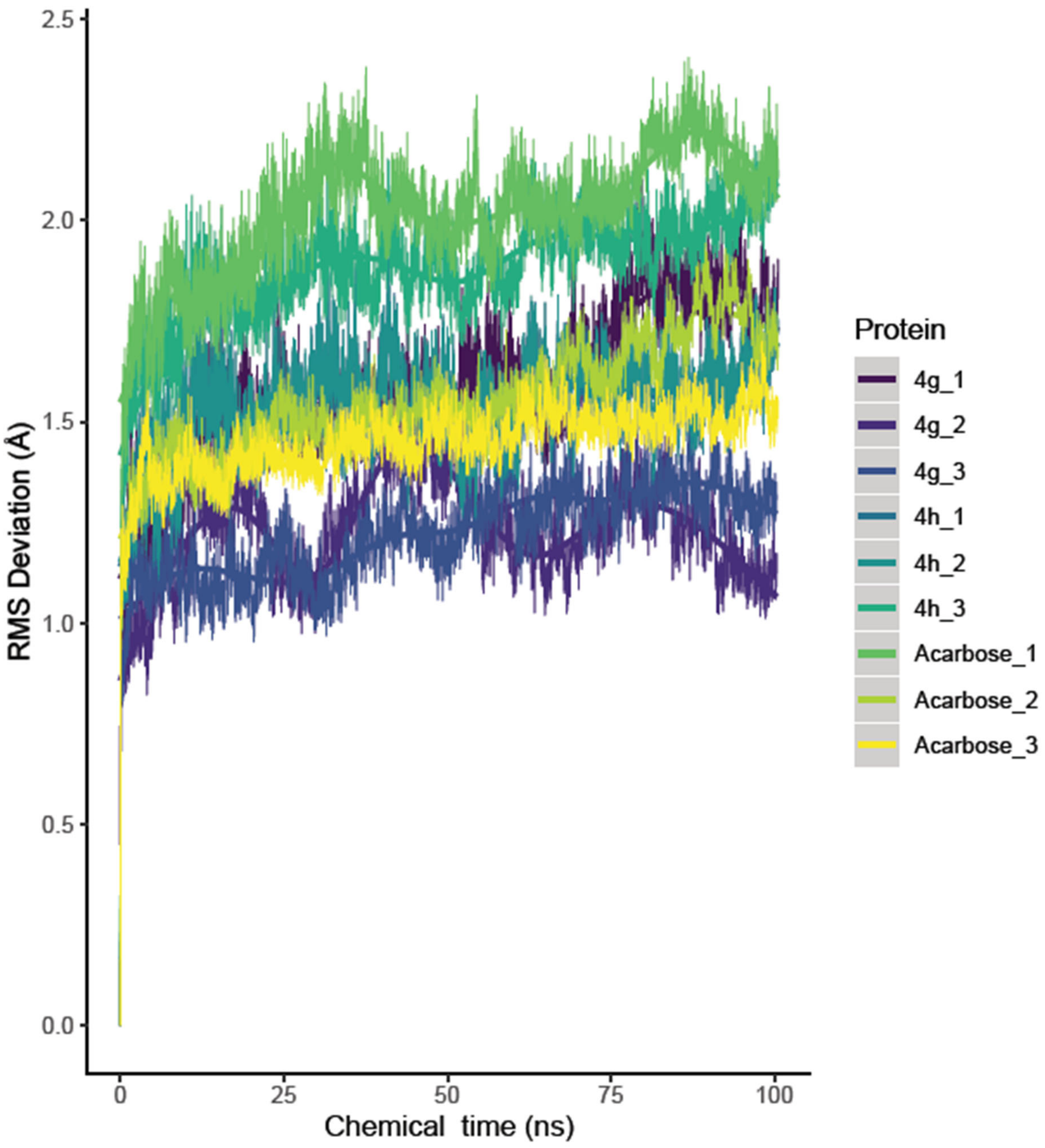

Figure S4a: RMSD plot

Molecular dynamics data

A

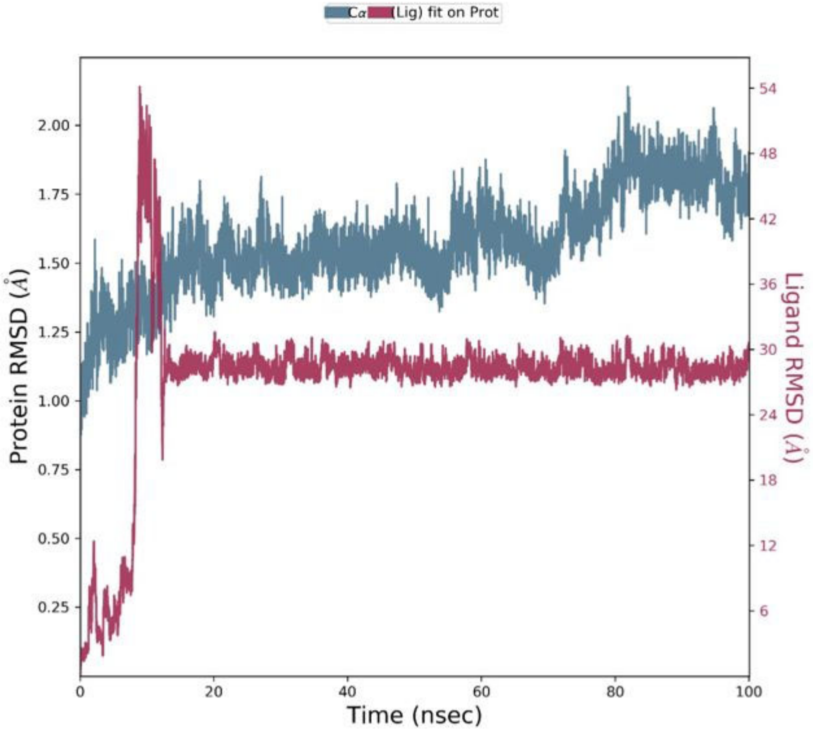

C

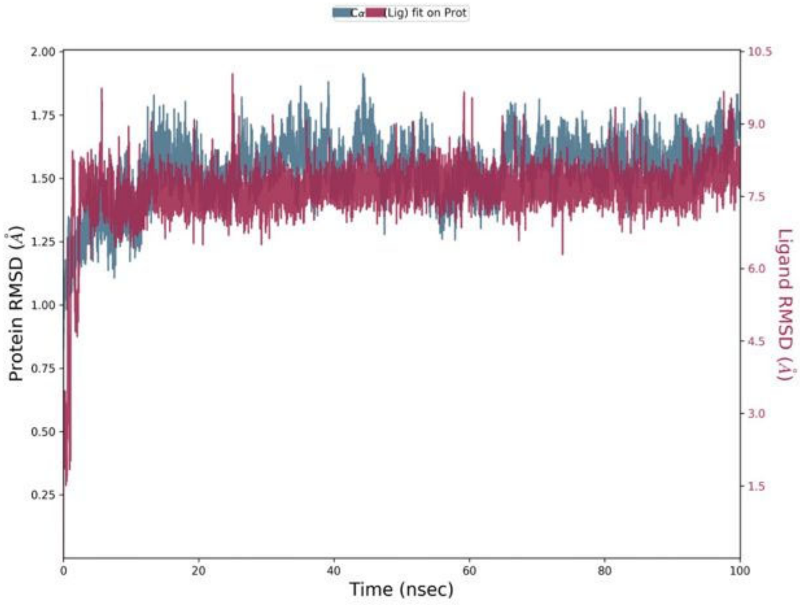

Figure S4b: Time-dependet RMSD plot of the αGLU enzyme (alpha-carbon) reflecting conformational equilibration of complexes along simulation of **4g** (A) and **4h** (C) deriatives. 2D interaction diagrams of the final ligand-binding interactions for **4g** (B) and **4h** (D) derivative.

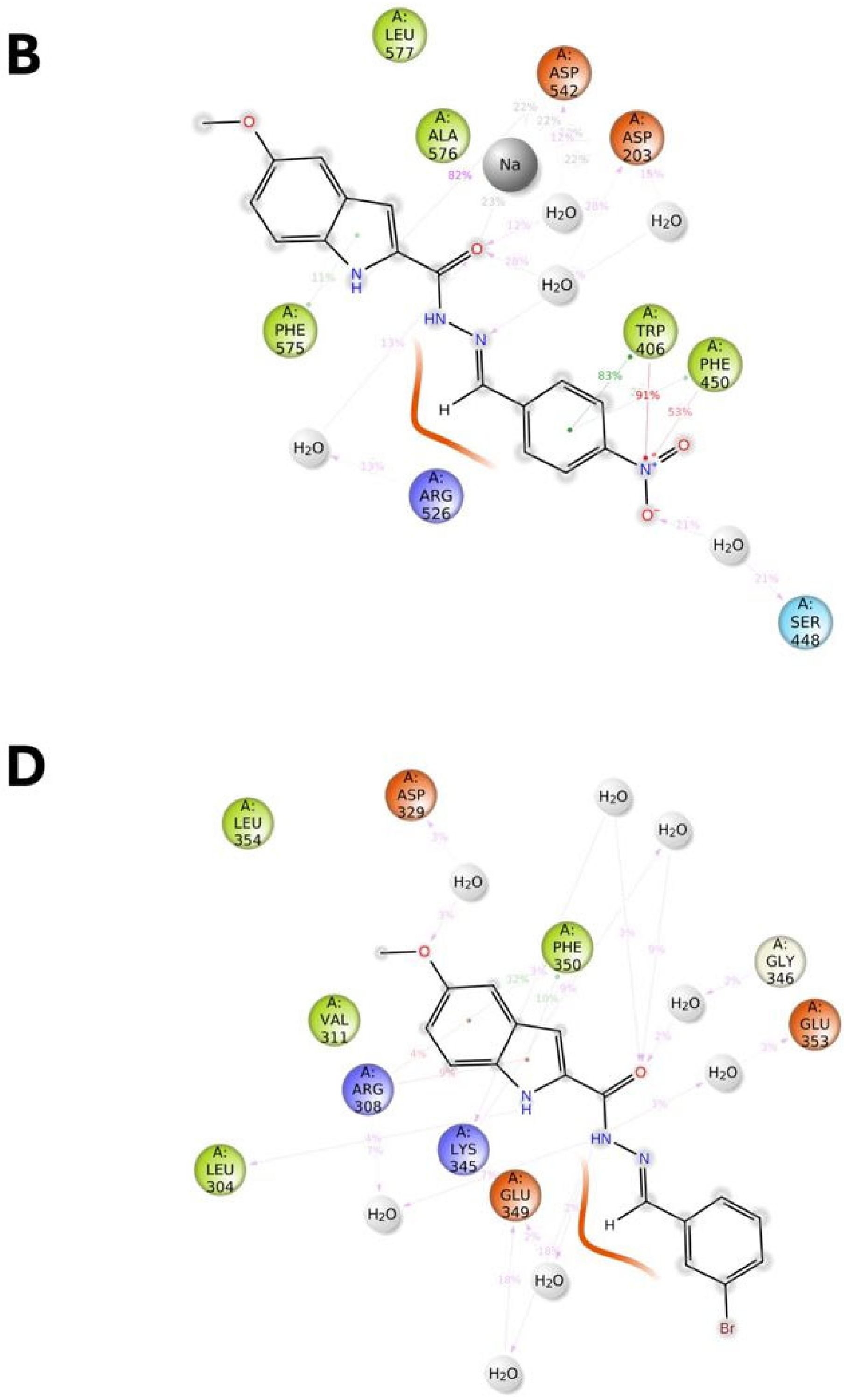

Figure S4c: 2D interaction diagrams of the final ligand-binding interactions for **4g** (B) and **4h** (D) derivative.

S4. ADMET Profiling of Selected Schiff Base Derivatives

Table S3: Summary of key ADMET properties for active compounds analyzed, including molecular weight, lipophilicity (logP), topological polar surface area (TPSA), absorption (Caco-2 permeability, human intestinal absorption, and bioavailability at 50%), distribution (plasma protein binding and volume of distribution), metabolism (CYP1A2 and CYP3A4 enzyme interactions), excretion (plasma clearance and half-life), and toxicity indicators (hERG blockade, Ames mutagenicity, drug-induced liver injury, and carcinogenicity).

Parameter	4e	4g	4h	4i	Acarbose	Comment
**(A) Physicochemical Property**						
Molecular Weight	339.12	371.03	338.1	343.13	645.25	Contain hydrogen atoms. Optimal: 100~600
Volume	338.638	323.045	329.702	359.116	573.315	Van der Waals volume
Density	1.001	1.149	1.025	0.955	1.125	Density = MW / Volume
nHA	7	5	8	5	19	Number of hydrogen bond acceptors. Optimal: 0~12
nHD	3	2	2	2	14	Number of hydrogen bond donors. Optimal: 0~7
nRot	6	5	6	5	9	Number of rotatable bonds. Optimal: 0~11
nRing	3	3	3	4	4	Number of rings. Optimal: 0~6
MaxRing	9	9	9	10	6	Number of atoms in the biggest ring. Optimal: 0~18
nHet	7	6	8	5	19	Number of heteroatoms. Optimal: 1~15
fChar	0	0	0	0	0	Formal charge. Optimal: -4~4
nRig	18	18	19	23	24	Number of rigid bonds. Optimal: 0~30
Flexibility	0.333	0.278	0.316	0.217	0.375	Flexibility = nRot / nRig
Stereo Centers	0	0	0	0	19	Stereo Centers. Optimal: ≤ 2
TPSA	95.94	66.48	109.62	66.48	321.17	Topological Polar Surface Area. Optimal: 0~140
logS	-4.876	-5.769	-5.43	-6.273	0.525	The logarithm of aqueous solubility value.
logP	3.184	4.126	2.925	3.845	-4.477	The logarithm of the n-octanol/water distribution coefficients at pH=7.4.
logD	3.207	3.82	3.241	3.73	-3.141	The logarithm of the n-octanol/water distribution coefficient.
pKa (Acid)	10.093	10.713	10.141	12.001	7.083	Acid-base dissociation constant (pKa) value represents the strength of a drug molecule's acidity or basicity.
pKa (Base)	3.919	3.853	3.13	3.691	5.625	Acid-base dissociation constant (pKa) value represents the strength of a drug molecule's acidity or basicity.

Parameter	4e	4g	4h	4i	Acarbose	Comment
Melting Point	198.966	193.867	233.486	256.845	190.822	The predicted melting point of a compound is expressed in degrees Celsius (°C). Solid if >25°C.
Boiling Point	366.619	382.366	396.567	422.688	442.946	The predicted boiling point of a compound is expressed in degrees Celsius (°C). Gas if <25°C.
**(B) Medicinal Chemistry**						
QED	0.492	0.542	0.423	0.433	0.103	A measure of drug-likeness based on desirability; Attractive: > 0.67; Unattractive: 0.49~0.67; Too complex: < 0.34
GASA	0	0	0	0	1	Easy to synthesize (ES); Hard to synthesize (HS). Value indicates the probability of being difficult to synthesize.
Synth	2	2	2	2	5	Synthetic accessibility score. Easy if <6; Difficult if ≥6.
Fsp3	0.111	0.059	0.059	0.048	0.92	The number of sp3 hybridized carbons / total carbon count. Fsp3 ≥0.42 is suitable.
MCE-18	17	16	17	20	89.333	Medicinal Chemistry Evolution score. Suitable if ≥45.
NPscore	-0.993	-1.632	-1.7	-1.243	1.829	Natural product-likeness score. Higher scores indicate higher natural product likelihood.
Lipinski Rule	0	0	0	0	1	MW ≤ 500; logP ≤ 5; Hacc ≤ 10; Hdon ≤ 5. One property out of range acceptable; two properties indicate poor absorption/permeability.
Pfizer Rule	0	1	0	1	0	logP > 3; TPSA < 75. Compounds with high logP and low TPSA are likely toxic.
GSK Rule	0	1	0	0	1	MW ≤ 400; logP ≤ 4. Satisfying the GSK rule indicates a favorable ADMET profile.
Golden Triangle	0	0	0	0	1	200 ≤ MW ≤ 500; -2 ≤ logD ≤ 5. Indicates a favorable ADMET profile.
PAINS	1 alerts	0 alerts	0 alerts	0 alerts	0 alerts	Frequent hitters, alpha-screen artifacts, or reactive compound substructures.
ALARM NMR	3 alerts	3 alerts	3 alerts	2 alerts	0 alerts	Thiol-reactive compounds.
BMS	0 alerts	0 alerts	0 alerts	0 alerts	1 alert	Indicates undesirable, reactive compounds.
Chelator Rule	0 alerts	0 alerts	0 alerts	0 alerts	0 alerts	Indicates chelating compounds.
Colloidal Aggregators	0.999	1	1	1	0.314	Category 0: non-colloidal aggregators; Category 1: colloidal aggregators.
**(C) Absorption**						
Caco-2	-5.394	-5.201	-5.421	-5.282	-7.289	Caco-2 Permeability; Optimal: higher than -5.15 Log unit

Parameter	4e	4g	4h	4i	Acarbose	Comment
MDCK Permeability	-5.082	-4.919	-4.972	-4.981	-4.852	Low permeability: < 2 × 10^{-6} cm/s; Medium: 2-20 × 10^{-6} cm/s; High: > 20 × 10^{-6} cm/s
PAMPA	0.887	0.964	0.892	0.981	1	Molecules with logPeff below 2.0 classified as low-permeability; above 2.5 as high-permeability
Pgp-inhibitor	0	0	0.002	0	0	Category 0: Non-inhibitor; Category 1: Inhibitor
Pgp-substrate	0.541	0.885	0.083	0.899	1	Category 0: Non-substrate; Category 1: Substrate
HIA	0	0	0	0	0.998	Human Intestinal Absorption. Category 1: HIA+ (<30%); Category 0: HIA- (≥30%)
F 20%	1	0.964	0.98	0.994	0.958	20% Bioavailability. Category 1: F 20%+ (<20%); Category 0: F 20%- (≥20%)
F 30%	0.301	0.283	0.228	0.614	1	30% Bioavailability. Category 1: F 30%+ (<30%); Category 0: F 30%- (≥30%)
F 50%	0.989	0.949	0.97	0.992	1	50% Bioavailability. Category 1: F 50%+ (<50%); Category 0: F 50%- (≥50%)
**(D) Distribution**						
PPB	93.401	98.439	97.463	98.841	15.221	Plasma Protein Binding. Optimal: <90%. High binding indicates a low therapeutic index.
VDss	-0.498	0.222	-0.145	-0.102	-0.576	Volume of Distribution. Optimal: 0.04–20 L/kg
BBB	0	0	0	0	0	Blood-Brain Barrier Penetration. Category 1: BBB+; Category 0: BBB-
Fu	4.354	1.46	1.842	0.533	81.75	Fraction unbound in plasma. Low: <5%; Medium: 5~20%; High: >20%
OATP1B1 inhibitor	0.012	0.006	0	0.002	0	Category 0: Non-inhibitor;
OATP1B3 inhibitor	1	0.984	0.964	0.972	0	Category 1: Inhibitor
BCRP inhibitor	0	0	0	0	0	
MRP1 inhibitor	0	0.004	0.023	0	0.006	
**(E) Metabolism**						
CYP1A2 inhibitor	0.996	0.983	0.949	0.91	0	Category 0: Non-inhibitor;
CYP1A2 substrate	0.889	0.99	0.845	0.936	0	Category 1: Inhibitor
CYP2C19 inhibitor	0.623	0.991	0.733	0.997	0	Category 0: Non-substrate;
CYP2C19 substrate	0.916	0.995	0.465	0.946	0	Category 1: Substrate
CYP2C9 inhibitor	0.032	0.552	0.059	0.048	0	
CYP2C9 substrate	0.848	0.322	0.59	0.649	0.001	
CYP2D6 inhibitor	0.001	0.002	0	0	0	
CYP2D6 substrate	0.63	0.001	0.467	0.148	0	
CYP3A4 inhibitor	0.162	0.016	0.012	0.016	0	
CYP3A4 substrate	0.006	0.586	0.023	0.027	0	
CYP2B6 inhibitor	0.016	0.067	0.025	0.032	0.002	
CYP2B6 substrate	0	0	0	0	0	
CYP2C8 inhibitor	0.982	0.997	1	1	0.992	

Parameter	4e	4g	4h	4i	Acarbose	Comment
HLM Stability	0.06	0.094	0.072	0.075	0.123	Human liver microsomal stability. Probability of instability: value closer to 1 indicates higher likelihood of instability.
**(F) Excretion**						
CL plasma	6.066	5.731	5.212	5.817	0.14	Plasma clearance in ml/min/kg. High clearance: >15 ml/min/kg; Moderate: 5–15 ml/min/kg; Low: <5 ml/min/kg
$T_1/_2$	0.695	0.573	0.633	0.515	3.635	Half-life (hours). Ultra-short: <1 hour; Short: 1–4 hours; Intermediate: 4–8 hours; Long: >8 hours
**(G) Toxicity**						
hERG Blockers	0.288	0.342	0.44	0.533	0.001	Probability of being hERG+. Molecules with IC50 ≤10 μM are classified as hERG+ (Category 1).
hERG Blockers (10 μM)	0.472	0.531	0.622	0.609	0.024	Probability of being hERG+. Molecules with IC50 ≤10 μM are classified as hERG+ (Category 1).
DILI	0.983	0.993	0.999	0.989	0.711	Drug-Induced Liver Injury. Category 1: High risk; Category 0: Low risk.
AMES Mutagenicity	0.901	0.836	0.99	0.962	0.885	AMES toxicity. Category 1: Ames positive (mutagenic); Category 0: Ames negative (non-mutagenic).
Rat Oral Acute Toxicity	0.492	0.591	0.644	0.655	0.002	Probability of being toxic. Low-toxicity: >500 mg/kg; High-toxicity: <500 mg/kg.
FDAMDD	0.829	0.891	0.801	0.897	0.001	FDA Maximum Daily Dose. Category 1: Positive; Category 0: Negative.
Skin Sensitization	0.068	0.282	0.756	0.113	0.998	Probability of being a skin sensitizer. Category 1: Sensitizer; Category 0: Non-sensitizer.
Carcinogenicity	0.87	0.872	0.935	0.936	0.003	Probability of being a carcinogen. Category 1: Carcinogenic; Category 0: Non-carcinogenic.
Eye Corrosion	0	0	0	0	0	Probability of causing eye corrosion. Category 1: Corrosive; Category 0: Non-corrosive.
Eye Irritation	0.778	0.918	0.92	0.611	0.02	Probability of causing eye irritation. Category 1: Irritant; Category 0: Non-irritant.
Respiratory	0.92	0.785	0.897	0.837	0.001	Probability of being a respiratory toxicant. Category 1: Toxicant; Category 0: Non-toxicant.
Human Hepatotoxicity	0.673	0.627	0.666	0.641	0.39	Probability of being hepatotoxic. Category 1: Positive; Category 0: Negative.

Parameter	4e	4g	4h	4i	Acarbose	Comment
Drug-Induced Nephrotoxicity	0.887	0.791	0.757	0.897	0.974	Probability of being nephrotoxic. Category 1: Nephrotoxic; Category 0: Non-nephrotoxic.
Ototoxicity	0.433	0.265	0.203	0.38	1	Probability of being ototoxic. Category 1: Ototoxic; Category 0: Non-ototoxic.
Hematotoxicity	0.65	0.536	0.668	0.55	0.31	Probability of being hematotoxic. Category 1: Hematotoxic; Category 0: Non-hematotoxic.
Genotoxicity	0.986	0.997	1	0.991	0.031	Probability of being genotoxic. Category 1: Genotoxic; Category 0: Non-genotoxic.
RPMI-8226 Immunotoxicity	0.245	0.115	0.07	0.098	0.292	Probability of being immunotoxic. Category 1: Cytotoxic; Category 0: Non-cytotoxic.
A549 Cytotoxicity	0.424	0.183	0.147	0.21	0.04	Probability of being cytotoxic. Category 1: Cytotoxic; Category 0: Non-cytotoxic.
Hek293 Cytotoxicity	0.742	0.613	0.66	0.607	0.021	Probability of being cytotoxic. Category 1: Cytotoxic; Category 0: Non-cytotoxic.
Drug-Induced Neurotoxicity	0.638	0.826	0.106	0.885	0	Probability of being neurotoxic. Category 1: Neurotoxic; Category 0: Non-neurotoxic.
**(H) Environmental Toxicity**						
Bioconcentration Factors	1.211	1.791	1.406	1.586	0.012	Used for assessing risks to human health via the food chain. Unit: -$\log_{10}$[(mg/L)/(1000*MW)].
IGC_{50}	3.986	4.318	4.151	4.526	1.613	Tetrahymena pyriformis 50% growth inhibition concentration. Unit: -$\log_{10}$[(mg/L)/(1000*MW)].
LC_{50} (FM)	4.881	5.639	5.196	6.09	2.352	96-hour fathead minnow 50% lethal concentration. Unit: -$\log_{10}$[(mg/L)/(1000*MW)].
LC_{50} (DM)	5.44	6.436	5.825	6.479	3.321	48-hour Daphnia magna 50% lethal concentration. Unit: -$\log_{10}$[(mg/L)/(1000*MW)].
**(I) Tox21 Pathway**						
NR-AhR	0.991	0.954	0.947	0.998	0.004	Aryl hydrocarbon receptor. Category 1: Active; Category 0: Inactive.
NR-AR	0.005	0.058	0.002	0.041	0.355	Androgen receptor. Category 1: Active; Category 0: Inactive.
NR-AR-LBD	0.023	0.076	0.012	0.063	0.357	Androgen receptor ligand-binding domain. Category 1: Active; Category 0: Inactive.
NR-Aromatase	0.007	0.032	0.017	0.059	0.002	Aromatase. Category 1: Active; Category 0: Inactive.
NR-ER	0.67	0.485	0.414	0.527	0.685	Estrogen receptor. Category 1: Active; Category 0: Inactive.

Parameter	4e	4g	4h	4i	Acarbose	Comment
NR-ER-LBD	0.007	0.011	0.005	0.007	0.098	Estrogen receptor ligand-binding domain. Category 1: Active; Category 0: Inactive.
NR-PPAR-gamma	0	0.007	0	0.004	0	Peroxisome proliferator-activated receptor gamma. Category 1: Active; Category 0: Inactive.
SR-ARE	0.642	0.711	0.438	0.728	0.01	Antioxidant response element. Category 1: Active; Category 0: Inactive.
SR-ATAD5	0.491	0.34	0.012	0.155	0.001	ATPase family AAA domain-containing protein 5. Category 1: Active; Category 0: Inactive.
SR-HSE	0.094	0.131	0.009	0.071	0	Heat shock factor response element. Category 1: Active; Category 0: Inactive.
SR-MMP	0.899	0.848	0.535	0.881	0.001	Mitochondrial membrane potential. Category 1: Active; Category 0: Inactive.
SR-p53	0.547	0.294	0.045	0.419	0.004	Tumor suppressor protein p53. Category 1: Active; Category 0: Inactive.
**(J) Toxicophore Rules**						
Acute Toxicity Rule	0	0	0	0	0	Contains 20 substructures related to acute toxicity during oral administration.
Genotoxic Carcinogenicity Rule	0	0	5 alerts	4 alerts	1 alert	Indicates carcinogenicity or mutagenicity based on 117 substructures.
Non-Genotoxic Carcinogenicity Rule	0	1 alerts	0	1 alerts	0	Indicates carcinogenicity through non-genotoxic mechanisms based on 23 substructures.
Skin Sensitization Rule	1 alerts	0	0	0	2 alerts	Indicates skin irritation based on 155 substructures.
Aquatic Toxicity Rule	0	1 alerts	0	0	0	Indicates toxicity to liquid (water) based on 99 substructures.
Non-Biodegradable Rule	0	0	2 alerts	0	1 alert	Indicates non-biodegradable compounds based on 19 substructures.
SureChEMBL Rule	0	0	0	0	0	Indicates MedChem unfriendly status based on 164 substructures.
FAF-Drugs4 Rule	6 alerts	5 alerts	7 alerts	5 alerts	1 alert	Indicates toxic substructures (154) as per FAF-Drugs4.

S5. Plots, Statistical Analysis and Visualizations

S5.1. Descriptive Statistics

Metrics: Mean, Standard Deviation (SD), and Coefficient of Variation

Table S4: Descriptive statistics

Compounds	A 450 nm			% inhibition			Mean	SD	CV
	R1	R2	R3	R1	R2	R3			
**4a**	1.511	1.661	1.629	41.141	20.726	33.393	31.75	8.42	26.501
**4b**	1.691	1.787	1.779	34.129	14.702	27.265	25.37	8.04	31.714
**4c**	1.543	1.679	1.522	39.907	19.862	37.768	32.51	8.99	27.644
**4d**	1.606	1.719	1.748	37.425	17.957	28.540	27.97	7.96	28.447
**4e**	0.313	0.484	0.442	87.826	76.893	81.926	82.21	4.47	5.435
**4f**	1.158	1.141	1.186	54.885	45.542	51.513	50.65	3.86	7.628
**4g**	0.184	0.203	0.244	92.817	90.334	90.041	91.06	1.25	1.367
**4h**	0.234	0.262	0.270	90.888	87.484	88.949	89.11	1.39	1.564
**4i**	0.408	0.418	0.390	84.106	80.043	84.052	82.73	1.90	2.300
**4j**	1.117	0.960	1.247	56.471	54.191	49.039	53.23	3.11	5.839
**Acarbose**	0.399	0.337	0.352	84.457	83.914	85.609	84.66	0.71	0.835
**Buffer**	2.567	2.095	2.446						

The mean % inhibition revealed that compounds **4g** (91.06%), **4h** (89.11%), **4e** (82.21%), and **4i** (82.73%) exhibited strong inhibitory activity, comparable to or exceeding the positive control, Acarbose (84.66%). These results suggest that these compounds are promising candidates for further investigation, as their inhibitory effects are among the most potent in the tested series. In

contrast, compounds **4b** (25.37%), **4d** (27.97%), and **4a** (31.75%) showed lower inhibition, indicating weaker activity relative to both Acarbose and the other compounds.

The variability among replicates, as indicated by the standard deviation (SD), was generally low for compounds with strong inhibition, such as Acarbose (SD: 0.87%), **4g** (SD: 1.53%), and **4h** (SD: 1.71%), suggesting high reproducibility. However, compounds like **4b** (SD: 9.85%) and **4d** (SD: 9.75%) displayed significantly higher variability, indicating potential inconsistencies in assay conditions or experimental handling.

To further interpret these findings, a coefficient of variation (CV) was calculated, providing a relative measure of variability normalized to the mean. Compounds with low CV values, such as Acarbose (CV: 1.02%), **4g** (CV: 1.67%), and **4h** (CV: 1.92%), demonstrated excellent reproducibility, whereas compounds like **4b** (CV: 38.84%) and **4d** (CV: 34.84%) highlighted the need for careful scrutiny of these data points.

Compounds **4g**, **4h**, **4e**, and **4i** emerged as strong and consistent inhibitors, with inhibitory activity comparable to or exceeding Acarbose. These findings underscore their potential as candidates for further evaluation. In contrast, compounds with high variability or lower % inhibition, such as **4b** and **4d**, may require additional optimization or replication to confirm their activity. These results provide a foundation for subsequent inferential statistical analyses and biological validation of the identified inhibitors.

5.2. Comparative Analysis

5.2.1. Comparison of Compounds

1) ANOVA Results:

F-statistic: 0.39

p-value: 0.681

The high p-value (> 0.05) indicates that there are no statistically significant differences in the mean % inhibition among the tested compounds, including Acarbose. This suggests that the inhibitory activities of the compounds are not significantly different from one another.

2) Tukey's Post-hoc Test Results

Tukey's post-hoc test was performed to identify specific pairs of compounds that exhibited statistically significant differences in % inhibition. The results are presented in Table S5.

Table S5: Tukey's post-hoc test results

Group 1	Group 2	Mean Diff	p-value	Lower CI	Upper CI	Reject Null
**4a**	**4b**	-6.388	0.9804	-26.0209	13.2449	FALSE
**4a**	**4c**	0.759	1	-18.8739	20.3919	FALSE
**4a**	**4d**	-3.7793	0.9997	-23.4122	15.8535	FALSE
**4a**	**4e**	50.4617	0	30.8288	70.0945	TRUE
**4a**	**4f**	18.8933	0.0661	-0.7395	38.5262	FALSE
**4a**	**4g**	59.3107	0	39.6778	78.9435	TRUE
**4a**	**4h**	57.3537	0	37.7208	76.9865	TRUE
**4a**	**4i**	50.9803	0	31.3475	70.6132	TRUE
**4a**	**4j**	21.4803	0.0243	1.8475	41.1132	TRUE
**4a**	Acarbose	52.9067	0	33.2738	72.5395	TRUE
**4b**	**4c**	7.147	0.9589	-12.4859	26.7799	FALSE

Group 1	Group 2	Mean Diff	p-value	Lower CI	Upper CI	Reject Null
**4b**	**4d**	2.6087	1	-17.0242	22.2415	FALSE
**4b**	**4e**	56.8497	0	37.2168	76.4825	TRUE
**4b**	**4f**	25.2813	0.0051	5.6485	44.9142	TRUE
**4b**	**4g**	65.6987	0	46.0658	85.3315	TRUE
**4b**	**4h**	63.7417	0	44.1088	83.3745	TRUE
**4b**	**4i**	57.3683	0	37.7355	77.0012	TRUE
**4b**	**4j**	27.8683	0.0017	8.2355	47.5012	TRUE
**4b**	Acarbose	59.2947	0	39.6618	78.9275	TRUE
**4c**	**4d**	-4.5383	0.9986	-24.1712	15.0945	FALSE
**4c**	**4e**	49.7027	0	30.0698	69.3355	TRUE
**4c**	**4f**	18.1343	0.0873	-1.4985	37.7672	FALSE
**4c**	**4g**	58.5517	0	38.9188	78.1845	TRUE
**4c**	**4h**	56.5947	0	36.9618	76.2275	TRUE
**4c**	**4i**	50.2213	0	30.5885	69.8542	TRUE
**4c**	**4j**	20.7213	0.0328	1.0885	40.3542	TRUE
**4c**	Acarbose	52.1477	0	32.5148	71.7805	TRUE
**4d**	**4e**	54.241	0	34.6081	73.8739	TRUE
**4d**	**4f**	22.6727	0.015	3.0398	42.3055	TRUE
**4d**	**4g**	63.09	0	43.4571	82.7229	TRUE
**4d**	**4h**	61.133	0	41.5001	80.7659	TRUE
**4d**	**4i**	54.7597	0	35.1268	74.3925	TRUE
**4d**	**4j**	25.2597	0.0052	5.6268	44.8925	TRUE
**4d**	Acarbose	56.686	0	37.0531	76.3189	TRUE
**4e**	**4f**	-31.5683	0.0004	-51.2012	-11.9355	TRUE
**4e**	**4g**	8.849	0.8614	-10.7839	28.4819	FALSE
**4e**	**4h**	6.892	0.9674	-12.7409	26.5249	FALSE
**4e**	**4i**	0.5187	1	-19.1142	20.1515	FALSE
**4e**	**4j**	-28.9813	0.0011	-48.6142	-9.3485	TRUE
**4e**	Acarbose	2.445	1	-17.1879	22.0779	FALSE
**4f**	**4g**	40.4173	0	20.7845	60.0502	TRUE
**4f**	**4h**	38.4603	0	18.8275	58.0932	TRUE
**4f**	**4i**	32.087	0.0003	12.4541	51.7199	TRUE
**4f**	**4j**	2.587	1	-17.0459	22.2199	FALSE
**4f**	Acarbose	34.0133	0.0001	14.3805	53.6462	TRUE

Group 1	Group 2	Mean Diff	p-value	Lower CI	Upper CI	Reject Null
**4g**	**4h**	-1.957	1	-21.5899	17.6759	FALSE
**4g**	**4i**	-8.3303	0.8989	-27.9632	11.3025	FALSE
**4g**	**4j**	-37.8303	0	-57.4632	-18.1975	TRUE
**4g**	Acarbose	-6.404	0.9801	-26.0369	13.2289	FALSE
**4h**	**4i**	-6.3733	0.9808	-26.0062	13.2595	FALSE
**4h**	**4j**	-35.8733	0.0001	-55.5062	-16.2405	TRUE
**4h**	Acarbose	-4.447	0.9988	-24.0799	15.1859	FALSE
**4i**	**4j**	-29.5	0.0009	-49.1329	-9.8671	TRUE
**4i**	Acarbose	1.9263	1	-17.7065	21.5592	FALSE
**4j**	Acarbose	31.4263	0.0004	11.7935	51.0592	TRUE

Compounds **4e**, **4g**, and **4h** demonstrated significantly higher inhibitory activity compared to weaker inhibitors, such as **4a**, **4b**, **4c**, and **4d**. For instance, **4e** showed a significant mean difference of 50.46% ($p < 0.05$) compared to **4a**, highlighting its superior potency. Similarly, **4g** and **4h** also exhibited strong inhibitory effects compared to weaker inhibitors.

When compared with Acarbose, **4g** and **4h** showed no statistically significant differences, suggesting that their inhibitory activity is comparable to the positive control. This finding underscores their potential as alternatives to Acarbose in α-glucosidase inhibition. Although **4e** displayed slightly lower inhibitory activity than Acarbose, the difference was statistically significant, reinforcing its efficacy.

In contrast, no significant differences were observed among the weaker inhibitors (**4a**, **4b**, **4c**, and **4d**), indicating similar levels of low potency. These compounds may require further optimization or structural modifications to enhance their inhibitory activity. Moreover, the consistency

observed among the high-inhibitory compounds (**4g**, **4h**, and Acarbose) reinforces their reliability as strong inhibitors under the tested conditions.

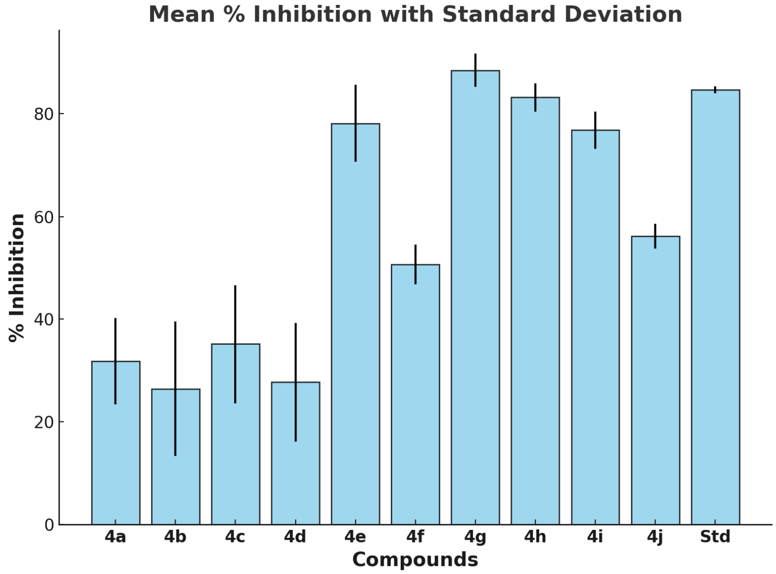

Figure S5: Bar graph showing the mean % inhibition of α-glucosidase activity for the tested compounds (**4a–4j**) and the standard (Acarbose, labeled as "Std"). Error bars represent the standard deviation (SD) across three replicates.

5.2.2. Comparison to Positive Control (Acarbose)

Unpaired t-Tests Results:

Unpaired t-tests were performed to compare the mean % inhibition of **α-glucosidase activity** for each compound against the positive control, **Acarbose**. This analysis aimed to identify compounds with statistically significant differences in inhibitory activity relative to the standard, thereby assessing their potential as effective alternatives.

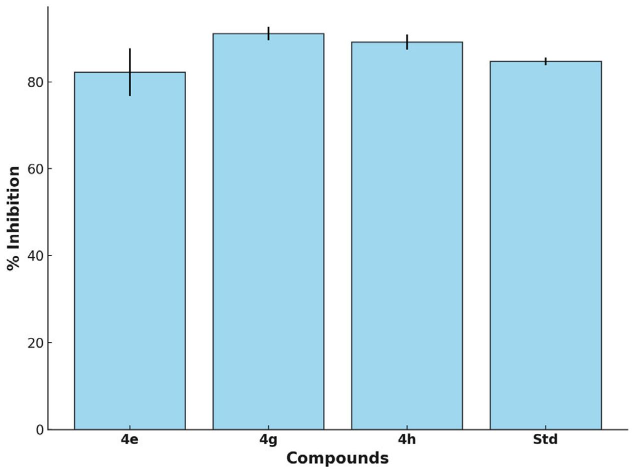

Figure S6: Bar graph comparing the % inhibition of α-glucosidase activity for the most active compounds (4e, 4g, 4h) and the standard (Acarbose, labeled as "Std").

The analysis revealed that compounds **4a**, **4b**, **4c**, **4d**, **4f**, **4g**, **4h**, and **4j** exhibited significantly lower % inhibition compared to Acarbose, with p-values < 0.05. For example, **4a** demonstrated a significant difference (t = -8.89, p = 0.012), reflecting its substantially weaker activity. Similar results were observed for **4b**, **4c**, and **4d**, highlighting their relatively limited inhibitory potential under the experimental conditions. These findings suggest that these compounds may require further optimization or structural modifications to enhance their potency.

In contrast, compounds **4e** and **4i** did not show statistically significant differences in % inhibition compared to Acarbose, indicating that their inhibitory efficacy is comparable to the standard. The lack of a significant difference underscores the potential of **4e** as an alternative inhibitor, meriting further investigation, particularly in terms of dose-response relationships and specific mechanisms of inhibition.

5.3. Variability and Reliability

Intra-Class Correlation Coefficient (ICC).

An ICC value of **0.835** indicates a high level of consistency among replicates within each compound. This suggests that the measurements for % inhibition are highly reliable and reproducible across the three replicates for each compound.

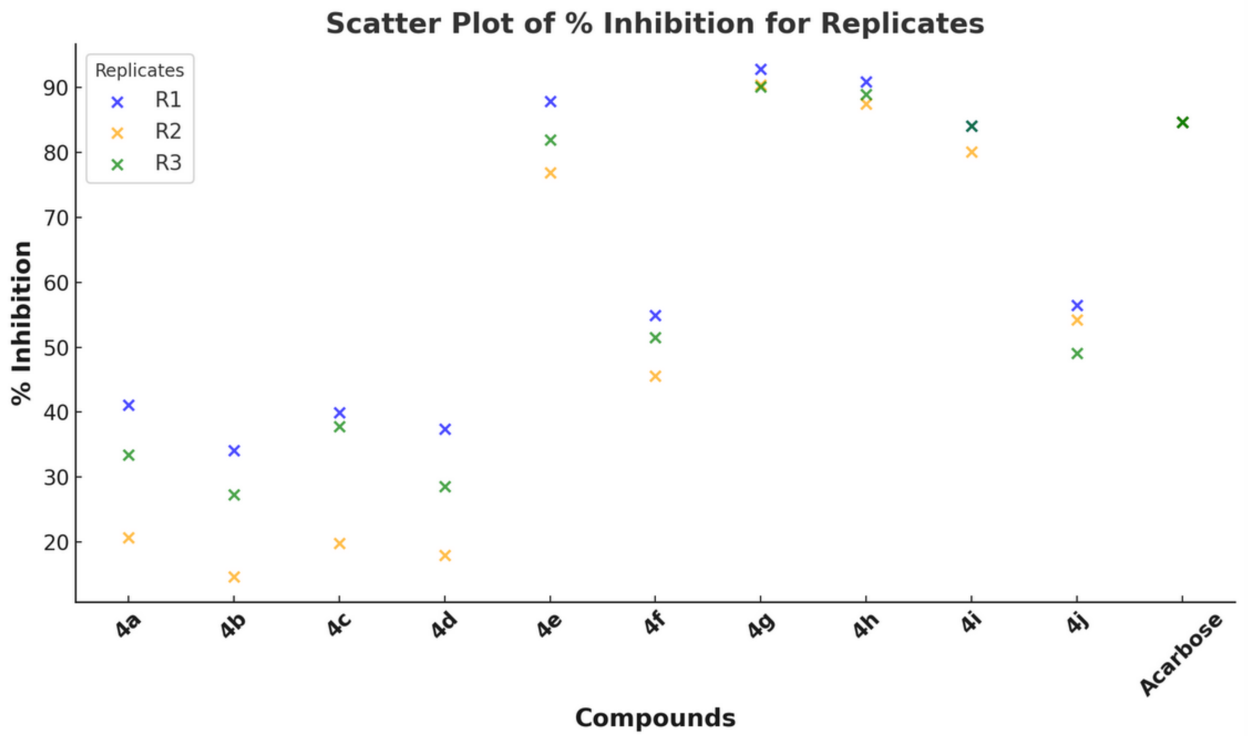

Figure S7: Scatter plot showing the % inhibition values of α-glucosidase activity for replicates (R1, R2, R3) across all tested compounds (**4a–4j**) and the standard (Acarbose).

6. Structure-Activity Relationship (SAR)

6.1. Comparative % Inhibition of Compounds

The activity threshold was set at % inhibition > 84% to include Acarbose (84.66%), the standard control, as a benchmark for evaluating the inhibitory performance of the tested compounds. Compounds exhibiting % inhibition greater than the threshold were categorized as active, while those below were considered less effective inhibitors under the tested conditions.

Figure S8 highlights that compounds **4g** (91.06%), **4h** (89.11%), **4e** (82.21%), and **4i** (82.73%) surpassed or matched the activity of Acarbose, indicating their superior inhibitory potential. These active compounds are distinguished in red, emphasizing their performance relative to other tested compounds. In contrast, compounds such as **4a**, **4b**, and **4c**, with % inhibition well below the threshold, demonstrated limited activity, suggesting weaker interactions with the enzyme.

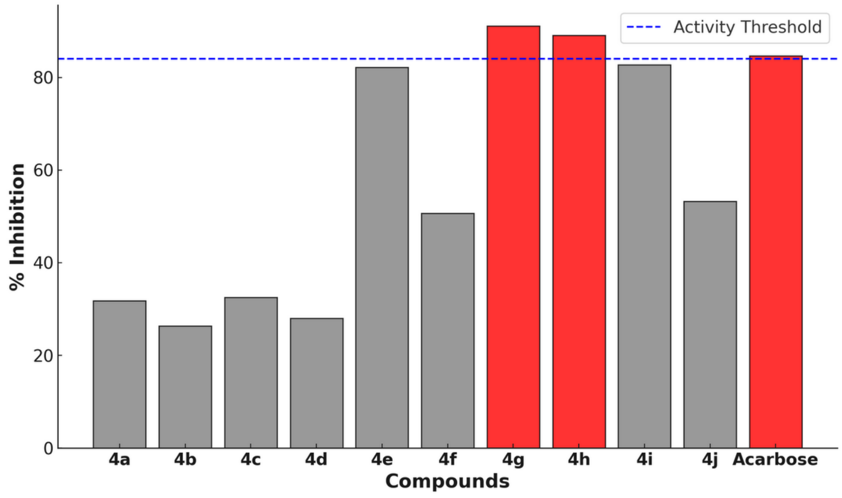

Figure S8: Comparative % inhibition of α-glucosidase activity for tested compounds (**4a–4j**) and Acarbose. Active compounds (% inhibition > 84%) are highlighted in red, with the activity threshold indicated as a dashed blue line.

The dashed blue line at 84% provides a clear visual reference for identifying active compounds. This threshold-based analysis underscores the significant role of structural features, such as nitro and bromine substitutions, in enhancing inhibitory activity.

Table S6 provides a comparative summary of the top-performing compounds (**4g**, **4h**, **4e**, and **4i**) and the standard control, Acarbose. The % inhibition values reflect the compounds' effectiveness against α-glucosidase activity, with **4g** (91.06%) and **4h** (89.11%) exhibiting inhibition levels comparable to or exceeding Acarbose (84.66%).

The table emphasizes the role of structural features in enhancing inhibitory activity. For instance, the halogen substitution in **4g** (3-Bromo) and the electron-withdrawing nitro group in **4h** are key contributors to their strong performance. Similarly, the hydroxyl and methoxy groups in **4e** positively influence its binding interactions, resulting in good activity (82.21%). The bulky naphthalene substitution in 4i highlights the importance of steric effects in modulating enzyme binding.

Table S6: Summary of functional groups and % inhibition of active compounds. Planar aromatic rings and electron-withdrawing halogen groups are key contributors to higher activity.

Compound	% Inhibition	Functional Group	Comments
**4g**	91.06	3-Bromo	Highest inhibition; halogen substitution enhances activity
**4h**	89.11	4-Nitro	Strong inhibition; electron-withdrawing nitro group enhances binding
**4e**	82.21	2-Hydroxy-4-Methoxy	Good activity; hydroxyl and methoxy groups contribute positively
**4i**	82.73	Naphthalene	Good activity; bulky aromatic substitution influences binding
Acarbose	84.66	Standard Control	Standard control; reliable benchmark for comparison

6.2. Molecular Weight vs % Inhibition

The relationship between molecular weight and % inhibition of α-glucosidase activity was investigated to assess whether larger compounds exhibit enhanced inhibitory activity. The % inhibition data for compounds **4a–4j** were plotted against their molecular weights, excluding the standard control (Acarbose). The activity threshold was set at 84% inhibition to identify active compounds, which were highlighted in red on the scatter plot (Figure S9).

The analysis revealed a moderate positive correlation between molecular weight and % inhibition, with a correlation coefficient (r) of 0.617. However, the p-value of 0.057 indicates that this correlation is not statistically significant. While heavier compounds, such as **4g** (molecular weight: 372.22 g/mol) and **4h** (338.32 g/mol), exhibited high % inhibition (91.06% and 89.11%, respectively), this trend was not uniform across all compounds. Notably, compounds **4a** and **4b**, with lower molecular weights (300.34 and 294.31 g/mol, respectively), displayed significantly lower inhibitory activity (31.75% and 26.37%).

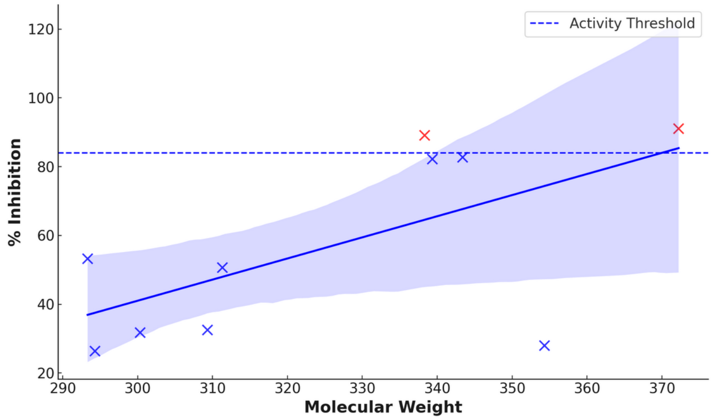

Figure S9: Scatter plot of molecular weight vs. % inhibition of α-glucosidase activity for tested compounds (**4a–4j**). Active compounds (% inhibition > 84%) are highlighted in red, with the activity threshold marked as a dashed blue line.

The scatter plot in Figure S9 includes a regression line with confidence intervals, providing a visual representation of the observed trend. The lack of a strong correlation suggests that molecular weight alone does not primarily govern inhibitory activity in this dataset. This underscores the importance of other factors, such as functional group composition, electronic effects, and spatial arrangement, in determining the binding efficiency and inhibitory potential of the compounds.

6.3. Functional Groups and Their Electronic Effects Analysis:

Table S7 provides a detailed summary of the functional groups present in compounds **4a–4j**, their electronic effects, and observations on their inhibitory activity. Functional groups were categorized based on their electronic effects as electron-donating groups (EDG), electron-withdrawing groups (EWG), or contributing to resonance stabilization.

Compounds with strong EWGs, such as **4h** (nitrobenzene) and **4g** (bromobenzene), demonstrated high inhibition (89.11% and 91.06%, respectively), highlighting the positive role of these groups in enhancing enzyme binding. Similarly, **4e** (hydroxy-methoxybenzene), with a combination of hydroxyl and methoxy groups (mixed EDG), showed good activity (82.21%), likely due to improved hydrogen bonding interactions.

In contrast, compounds with weaker EWGs, such as **4a** (thiazole) and **4b** (pyridine), exhibited low inhibition, suggesting limited binding interactions with the enzyme. Compounds **4i** (naphthalene) and **4j** (benzene), which benefit from resonance stabilization, displayed moderate to good activity (82.73% and 53.23%, respectively), emphasizing the importance of structural stability in enzyme interaction.

Table S7: Summary of functional groups, electronic effects, and their impact on % inhibition. Moderate EWG groups (halogens) and resonance stabilization are key contributors to high activity.

Compound	Functional Group	Electronic Effect	Observation
**4a**	Thiazole	Weak EWG	Low inhibition; weak interaction with enzyme
**4b**	Pyridine	Moderate EWG	Low inhibition; moderate EWG limits activity
**4c**	Hydroxybenzene	EDG (OH)	Moderate inhibition; hydroxyl enhances binding
**4d**	Hydroxy-nitrobenzene	Strong EWG (NO_2)	Low inhibition; nitro group likely disrupts binding
**4e**	Hydroxy-methoxybenzene	Mixed EDG (OH, OCH_3)	Good inhibition; hydroxyl and methoxy groups enhance activity
**4f**	Fluorobenzene	Weak EWG (F)	Moderate inhibition; fluorine contributes to binding
**4g**	Bromobenzene	Moderate EWG (Br)	High inhibition; bromine enhances activity via halogen bonding
**4h**	Nitrobenzene	Strong EWG (NO_2)	High inhibition; nitro group enhances binding efficiency
**4i**	Naphthalene	Resonance Stabilization	Good inhibition; resonance stabilization contributes to activity
**4j**	Benzene	Resonance Stabilization	Moderate inhibition; resonance stabilization supports activity

6.4. Impact of Electronic Effects on % Inhibition

Figure S10 illustrates the relationship between electronic effects of functional groups and % inhibition of α-glucosidase activity for the tested compounds. The compounds were categorized

into five groups based on their electronic effects: weak EWGs, moderate EWGs, strong EWGs, mixed EDGs, and resonance-stabilized groups.

The boxplot highlights that resonance-stabilized compounds, such as **4i** and **4j**, tend to exhibit relatively high inhibitory activity, likely due to enhanced stability and effective binding to the enzyme's active site. Compounds with moderate EWGs, including **4g** (bromobenzene), show the highest median activity, indicating the positive contribution of halogen atoms to enzyme inhibition.

In contrast, weak EWGs (e.g., **4a**, **4b**) and strong EWGs (e.g., **4h**) exhibit variable activity levels, suggesting that the strength of electron-withdrawing effects does not always correlate directly with inhibitory performance. Mixed EDGs, represented by **4e**, display intermediate activity, driven by the combined effects of hydroxyl and methoxy substituents.

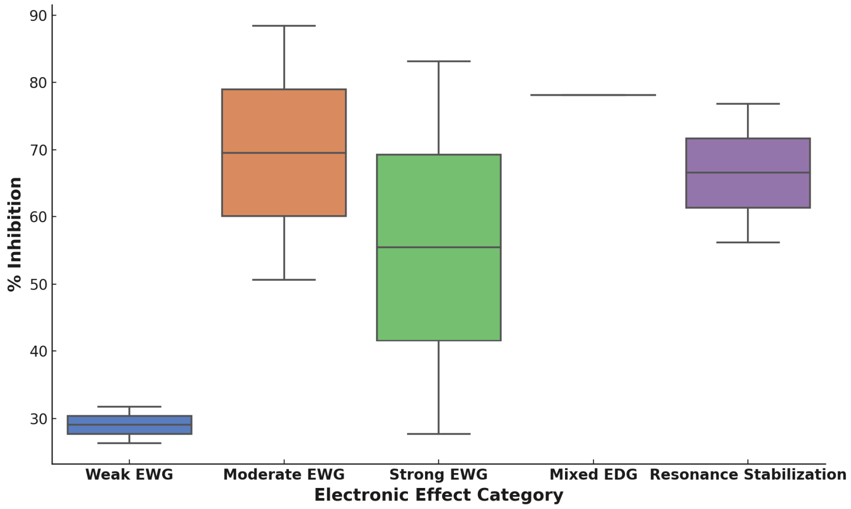

Figure S10: Boxplot showing the impact of electronic effects on % inhibition of α-glucosidase activity for tested compounds (**4a–4j**).

6.5. Structure-Activity Relationship (SAR) Trends of Active Compounds

Table S8 summarizes the SAR trends for the most active compounds (**4g**, **4h**, **4e**, and **4i**) based on their functional groups, electronic effects, and observed inhibitory activity. These compounds were selected for their high % inhibition values (>76%), indicating strong α-glucosidase inhibitory potential.

4g (91.06%) exhibited the highest activity among the tested compounds. Its bromobenzene functional group, characterized by moderate electron-withdrawing effects, likely enhances enzyme binding through halogen bonding. Similarly, **4h** (89.11%) benefits from a strong electron-withdrawing nitro group, which improves binding efficiency through enhanced electronic interactions.

4e (82.21%), featuring hydroxy-methoxybenzene, leverages mixed EDG effects to facilitate hydrogen bonding with the enzyme's active site, contributing to its inhibitory activity. Finally, **4i** (82.73%) achieves good activity through resonance stabilization provided by its naphthalene group, which improves structural stability and interaction with the enzyme.

Table S8: SAR trends for active compounds, highlighting % inhibition, functional groups, electronic effects, and key trends that influence α-glucosidase inhibitory activity.

Compound	% Inhibition	Functional Group	Electronic Effect	Key Trends
**4g**	91.06	Bromobenzene	Moderate EWG (Br)	Halogen bonding enhances activity
**4h**	89.11	Nitrobenzene	Strong EWG (NO_2)	Electron-withdrawing nitro group improves binding
**4e**	82.21	Hydroxy-methoxybenzene	Mixed EDG (OH, OCH_3)	Hydroxyl and methoxy groups facilitate hydrogen bonding
**4i**	82.73	Naphthalene	Resonance Stabilization	Resonance stabilization contributes to activity
**Acarbose**	84.66	Standard	-	Positive control, benchmark.

6.6. Steric Effects on % Inhibition:

Table S9 provides a comprehensive summary of steric bulk, functional groups, and % inhibition for all tested compounds (**4a–4j**). The analysis demonstrates that compounds with moderate to large steric bulk, such as **4g** (bromobenzene, 91.06%), **4h** (nitrobenzene, 89.11%), **4i** (naphthalene, 82.73%), achieve high inhibitory activity. These findings suggest that increased steric bulk facilitates optimal interactions with the enzyme's active site, enhancing inhibitory potential.

In contrast, compounds with smaller functional groups, such as **4a** (thiazole, 31.75%) and **4b** (pyridine, 26.37%), exhibit significantly lower activity, likely due to reduced binding efficiency. Moderately bulky compounds, such as **4e** (hydroxy-methoxybenzene, 82.21%), benefit from a balance of steric and electronic effects, contributing to their high activity. These trends highlight the importance of steric effects in modulating enzyme binding and provide a framework for designing inhibitors with enhanced potency.

Table S9: Steric bulk, functional groups, and % inhibition for all tested compounds (**4a–4j**), illustrating the impact of steric effects on α-glucosidase inhibitory activity.

Compound	Steric Bulk	Functional Group	Observation
**4a**	Small (Thiazole)	Thiazole	Low activity due to weak interaction with the enzyme
**4b**	Small (Pyridine)	Pyridine	Low activity; limited steric and electronic contributions
**4c**	Small (Hydroxybenzene)	Hydroxybenzene	Moderate activity; hydroxyl enhances binding

Compound	Steric Bulk	Functional Group	Observation
**4d**	Moderate (Hydroxy-nitrobenzene)	Hydroxy-nitrobenzene	Low activity; nitro group disrupts binding
**4e**	Moderate (Hydroxy-methoxybenzene)	Hydroxy-methoxybenzene	High activity; optimal steric and electronic balance
**4f**	Small (Fluorobenzene)	Fluorobenzene	Moderate activity; fluorine enhances binding moderately
**4g**	Moderate (Bromobenzene)	Bromobenzene	High activity; bromine contributes via halogen bonding
**4h**	Moderate (Nitrobenzene)	Nitrobenzene	High activity; nitro group enhances binding
**4i**	Large (Naphthalene)	Naphthalene	High activity; resonance stabilization contributes
**4j**	Small (Benzene)	Benzene	Moderate activity; benzene allows basic interaction

Steric Hindrance: Figure S11a highlights the relationship between steric hindrance and % inhibition of α-glucosidase activity. Compounds with moderate steric hindrance, such as **4e** (hydroxy-methoxybenzene) and **4g** (bromobenzene), exhibit the highest inhibitory activity, indicating that moderate steric bulk optimally facilitates enzyme binding. In contrast, compounds with small steric bulk, such as **4a** (thiazole), demonstrate significantly lower activity, likely due to limited interactions with the enzyme active site. Excessive steric hindrance, as observed in **4i** (naphthalene), slightly reduces activity, potentially due to disruptions in effective binding.

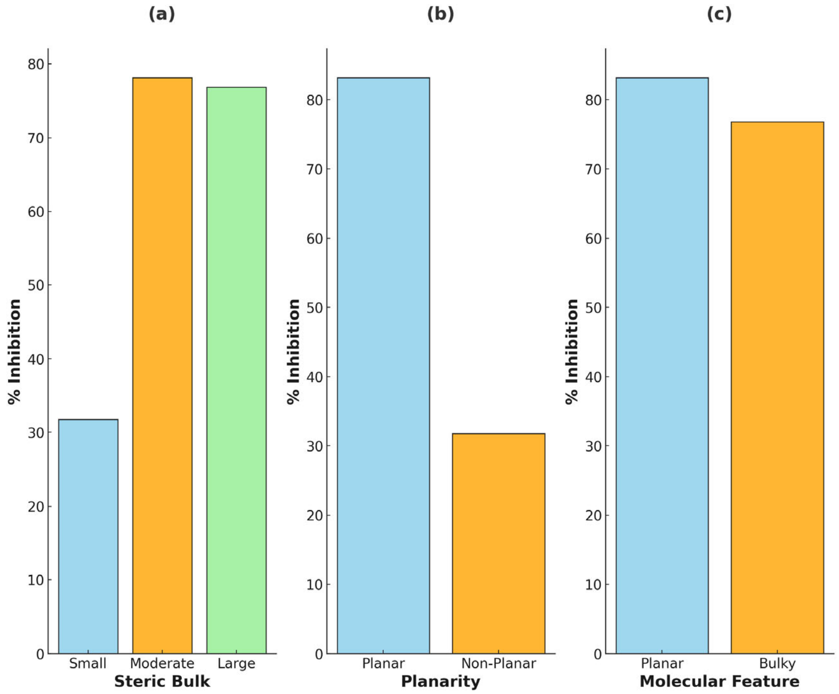

Figure S11: Impact of molecular features on % inhibition of α-glucosidase activity: (a) Steric hindrance; (b) Molecular planarity; (c) Planarity vs. Bulk. The panels illustrate the roles of steric bulk and molecular planarity in modulating inhibitory activity.

Planarity: The significance of molecular planarity is illustrated in Figure S11b. Planar compounds, such as **4g** and **4h** (nitrobenzene), achieve consistently high activity due to their ability to establish stable interactions within the enzyme's active site. In contrast, non-planar compounds, such as **4a**, show significantly reduced activity, emphasizing the importance of planarity in optimizing enzyme-ligand binding.

Comparison of Planar vs. Bulky Groups: Figure S11c compares the inhibitory activity of planar and bulky compounds. Planar compounds, such as **4h** and **4g**, outperform bulky structures, such as **4i**, despite the latter's favorable steric bulk. This finding underscores the critical role of molecular planarity in enhancing biological activity. The interplay between planarity and steric effects offers valuable insights for designing α-glucosidase inhibitors with optimized structural and electronic features.

6.7. Binding Energies and Molecular Interactions

The relationship between binding free energies (BFE) and % inhibition was analyzed to determine the strength of molecular interactions between the compounds and the target enzyme. Lower binding free energy values (more negative) typically indicate stronger binding affinity and a higher likelihood of inhibitory activity.

Figure S2 and Table S4 present the binding free energies (BFE), their standard deviations, and the associated binding affinity (AFF) values in pK units. The compounds with the most favorable binding energies include **4h** (-6.77 kcal/mol), **4g** (-6.75 kcal/mol), and **4e** (-6.37 kcal/mol). Notably, these compounds also exhibit high inhibitory activity (% inhibition > 84%), supporting the correlation between lower BFE values and stronger biological activity.

Additionally, compound **4i** (-6.43 kcal/mol) also shows a favorable binding free energy, aligning with its strong enzyme inhibition (82.73%). The standard inhibitor Acarbose remains the reference point with a BFE of -7.40 kcal/mol, further validating the computational predictions.

These results suggest that halogen substitutions (**4g**, **4h**), hydroxyl/methoxy groups (**4e**), and bulky aromatic systems (**4i**) contribute significantly to enhanced enzyme binding and inhibitory potency. The electronic effects and spatial orientation of these functional groups likely facilitate key interactions within the enzyme's active site, reinforcing the importance of molecular docking studies in structure-based drug design.

6.8 HOMO-LUMO Gap Analysis

The HOMO-LUMO energy gaps (ΔE) values ranged from 6.20 eV to 7.71 eV, reflecting variations in electronic excitation thresholds across the series. Lower ΔE values are typically associated with

higher electronic reactivity, which could influence molecular interactions with the α-glucosidase enzyme.

However, a direct correlation between ΔE values and biological activity (% inhibition or IC_{50}) was not observed. For instance, **4d** (ΔE = 6.20 eV) displayed low inhibition (27.97%), whereas **4g** (ΔE = 7.01 eV) showed high inhibition (91.06%), despite a relatively higher ΔE value. Similarly, **4h** (ΔE = 6.23 eV) exhibited strong inhibition (89.11%), reinforcing that electronic properties alone are not sufficient to predict inhibitory potential.

Conversely, compounds with higher ΔE values, such as **4e** (7.71 eV) and **4j** (7.14 eV), still demonstrated strong inhibitory activity (82.21% and 53.23%, respectively). These findings suggest that steric effects, molecular interactions, and binding site complementarity play critical roles alongside electronic factors in determining biological activity.

These observations underscore the complexity of enzyme inhibition mechanisms, emphasizing the necessity of integrating electronic property analyses, such as ΔE, with binding affinities, molecular docking, and SAR studies to derive a comprehensive understanding of structure-activity relationships (SAR).

6.9 MEP Correlation Analysis

To investigate the correlation between MEP and inhibitory activity, the electrostatic potential values at key molecular regions were compared to the % inhibition data. Compounds with strong electron-withdrawing groups (EWGs), such as **4h** (NO_2) and **4g** (Br), exhibited highly polarized MEP distributions, potentially enhancing enzyme-ligand electrostatic complementarity. Notably,

4h and **4g** demonstrated high inhibition (89.11% and 91.06%), supporting the role of electrostatic interactions in enzyme binding.

Conversely, compounds with weaker EWGs or EDGs, such as **4a** (thiazole) and **4b** (pyridine), exhibited less polarized MEP surfaces, correlating with their lower inhibitory activity (31.75% and 26.37%). The resonance-stabilized systems, **4i** (naphthalene) and **4j** (phenyl), displayed moderate electrostatic potential distributions, which may contribute to their intermediate inhibition profiles.

These findings emphasize that MEP-based charge distributions significantly influence molecular recognition and binding affinity. However, MEP alone is not the sole determinant of inhibition, as steric hindrance, solvation effects, and dynamic protein-ligand interactions also contribute to biological activity. Further quantum chemical analyses and docking simulations are needed to refine this correlation and optimize molecular design strategies for improved enzyme inhibition.

7. NMR Spectra

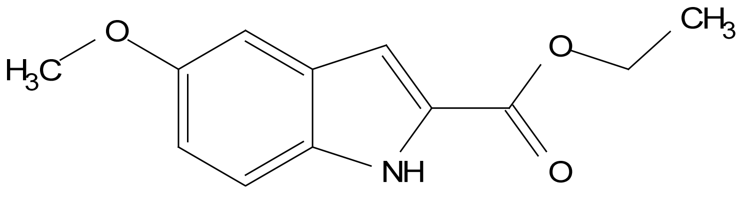
H3C
O
NH
O
O
CH3

2

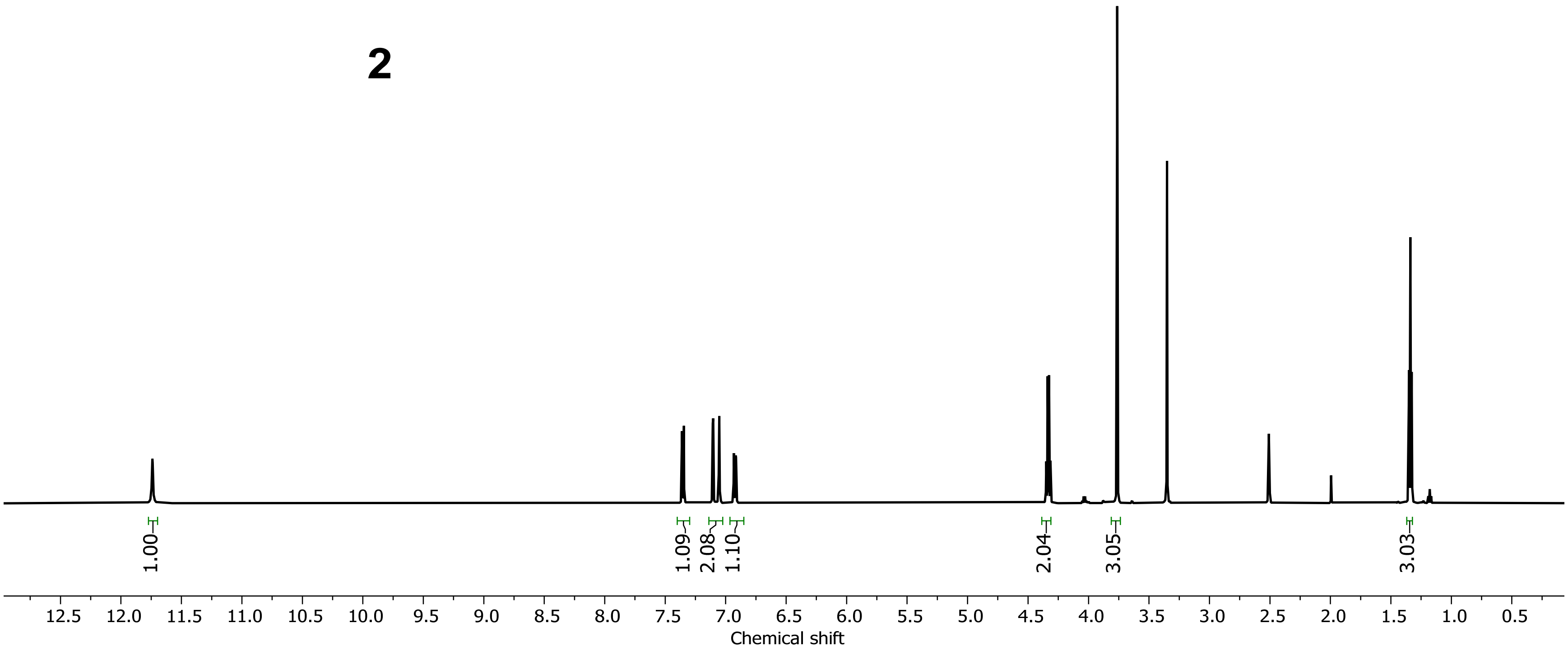
1.00
1.09
2.08
1.10
2.04
3.05
3.03
12.5 12.0 11.5 11.0 10.5 10.0 9.5 9.0 8.5 8.0 7.5 7.0 6.5 6.0 5.5 5.0 4.5 4.0 3.5 3.0 2.5 2.0 1.5 1.0 0.5
Chemical shift

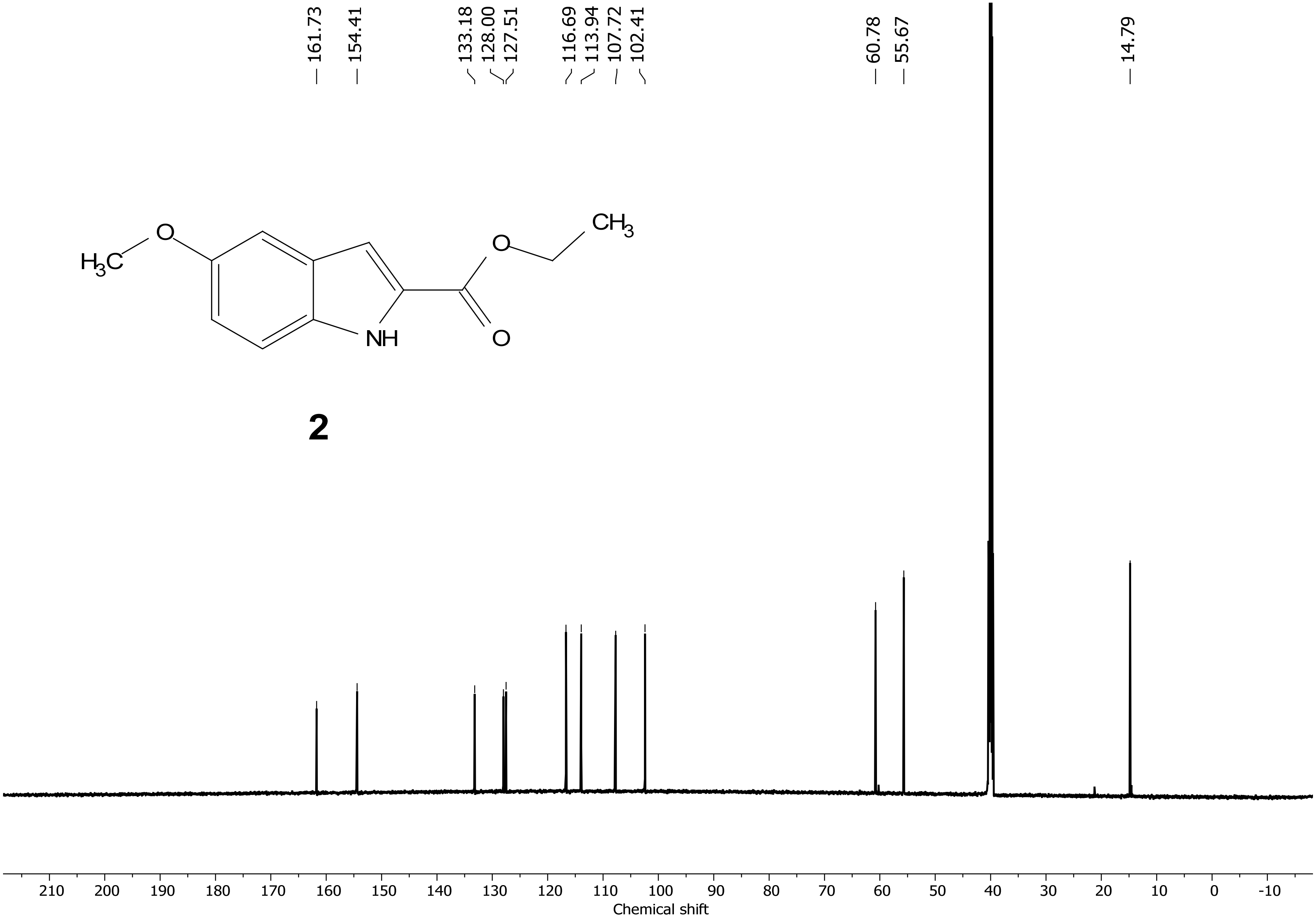

161.73
154.41
133.18
128.00
127.51
116.69
113.94
107.72
102.41
60.78
55.67
14.79
H3C
O
CH3
O
NH
O
2
210 200 190 180 170 160 150 140 130 120 110 100 90 80 70 60 50 40 30 20 10 0 -10
Chemical shift

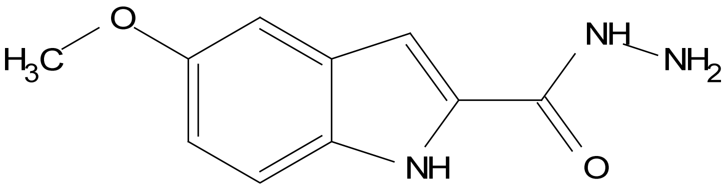

3

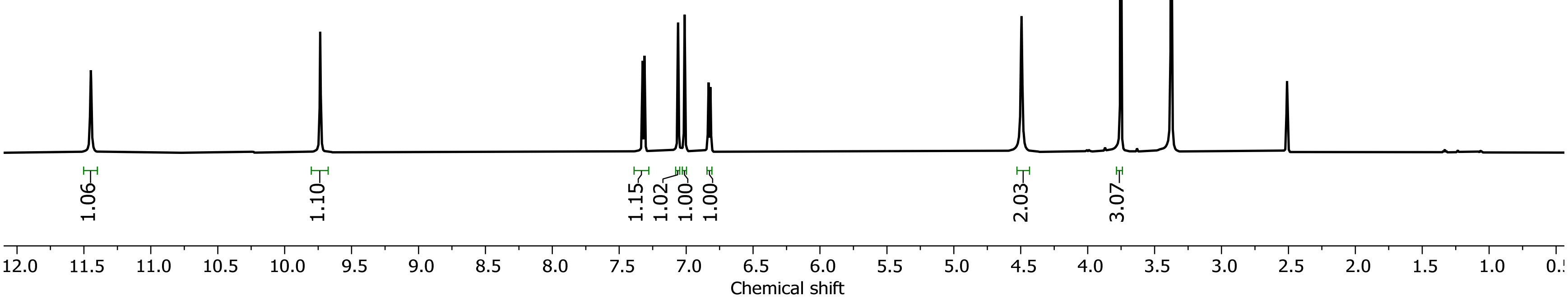

1.06
1.10
1.15
1.02
1.00
1.00
2.03
3.07
12.0 11.5 11.0 10.5 10.0 9.5 9.0 8.5 8.0 7.5 7.0 6.5 6.0 5.5 5.0 4.5 4.0 3.5 3.0 2.5 2.0 1.5 1.0
Chemical shift

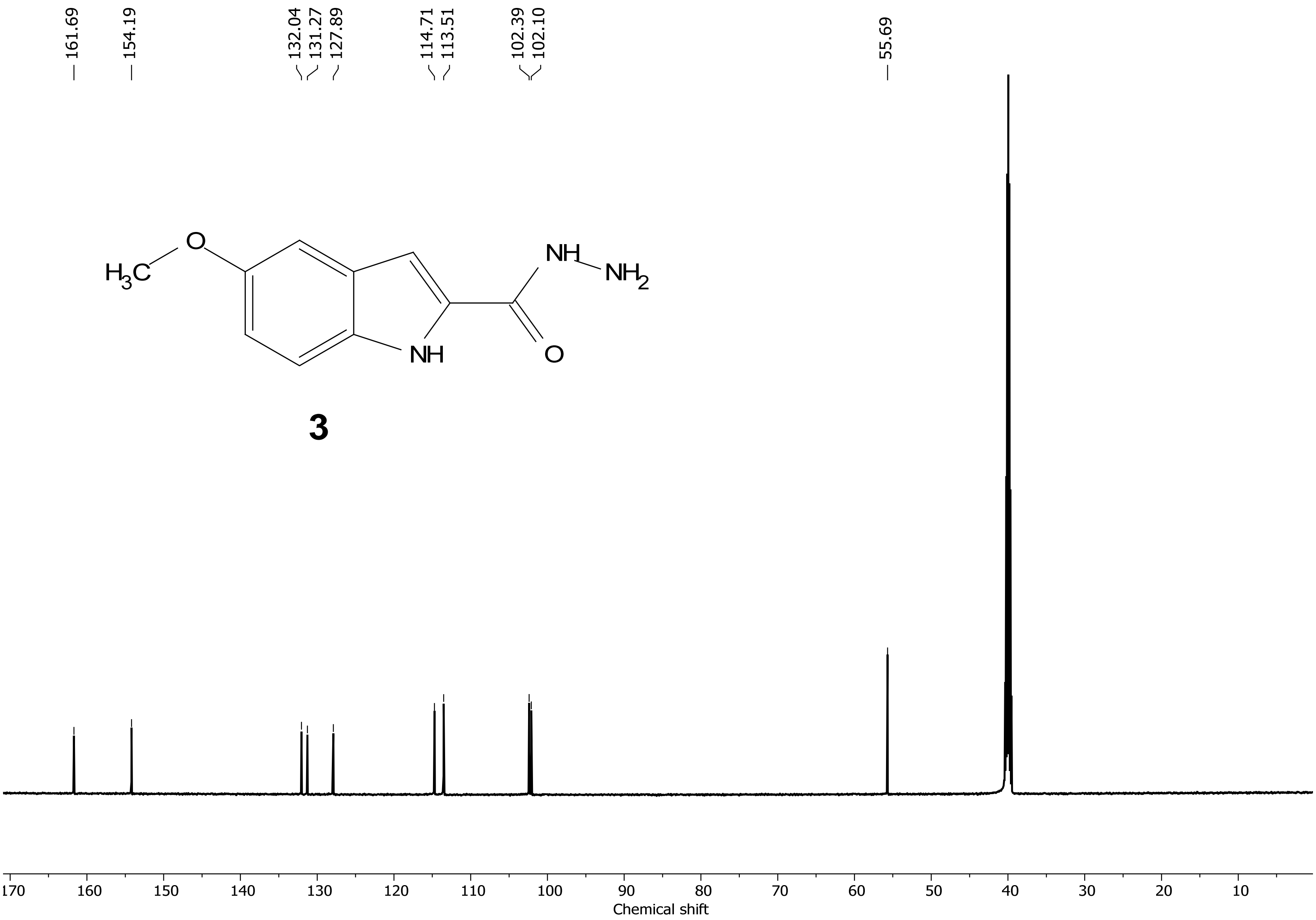
161.69
154.19
132.04
131.27
127.89
114.71
113.51
102.39
102.10
55.69
H_3C
O
NH
NH_2
NH
O
3
170 160 150 140 130 120 110 100 90 80 70 60 50 40 30 20 10
Chemical shift

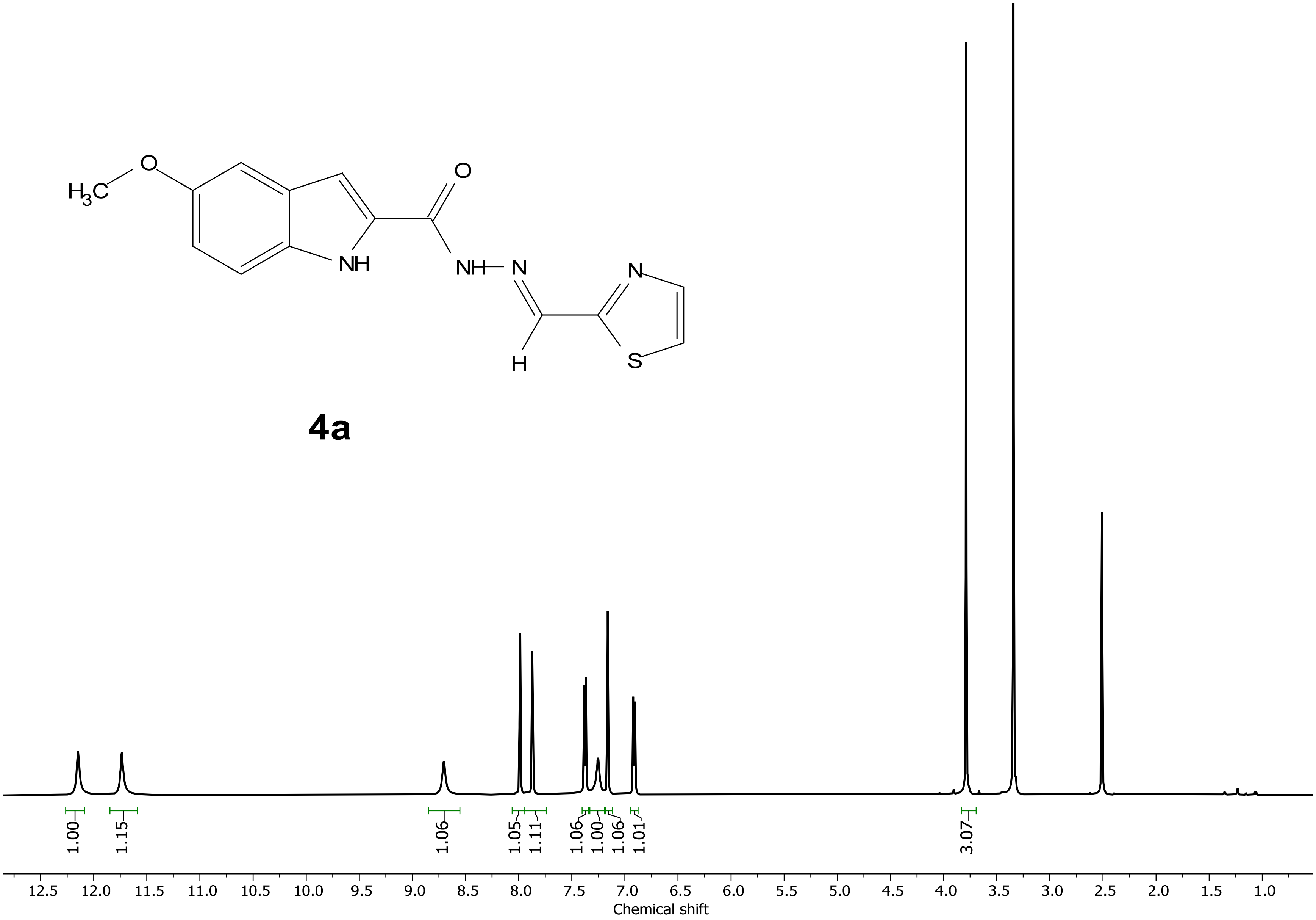

H3C
O
O
NH
NH
N
N
H
S
4a
1.00
1.15
1.06
1.05
1.11
1.06
1.00
1.06
1.01
3.07
12.5 12.0 11.5 11.0 10.5 10.0 9.5 9.0 8.5 8.0 7.5 7.0 6.5 6.0 5.5 5.0 4.5 4.0 3.5 3.0 2.5 2.0 1.5 1.0
Chemical shift

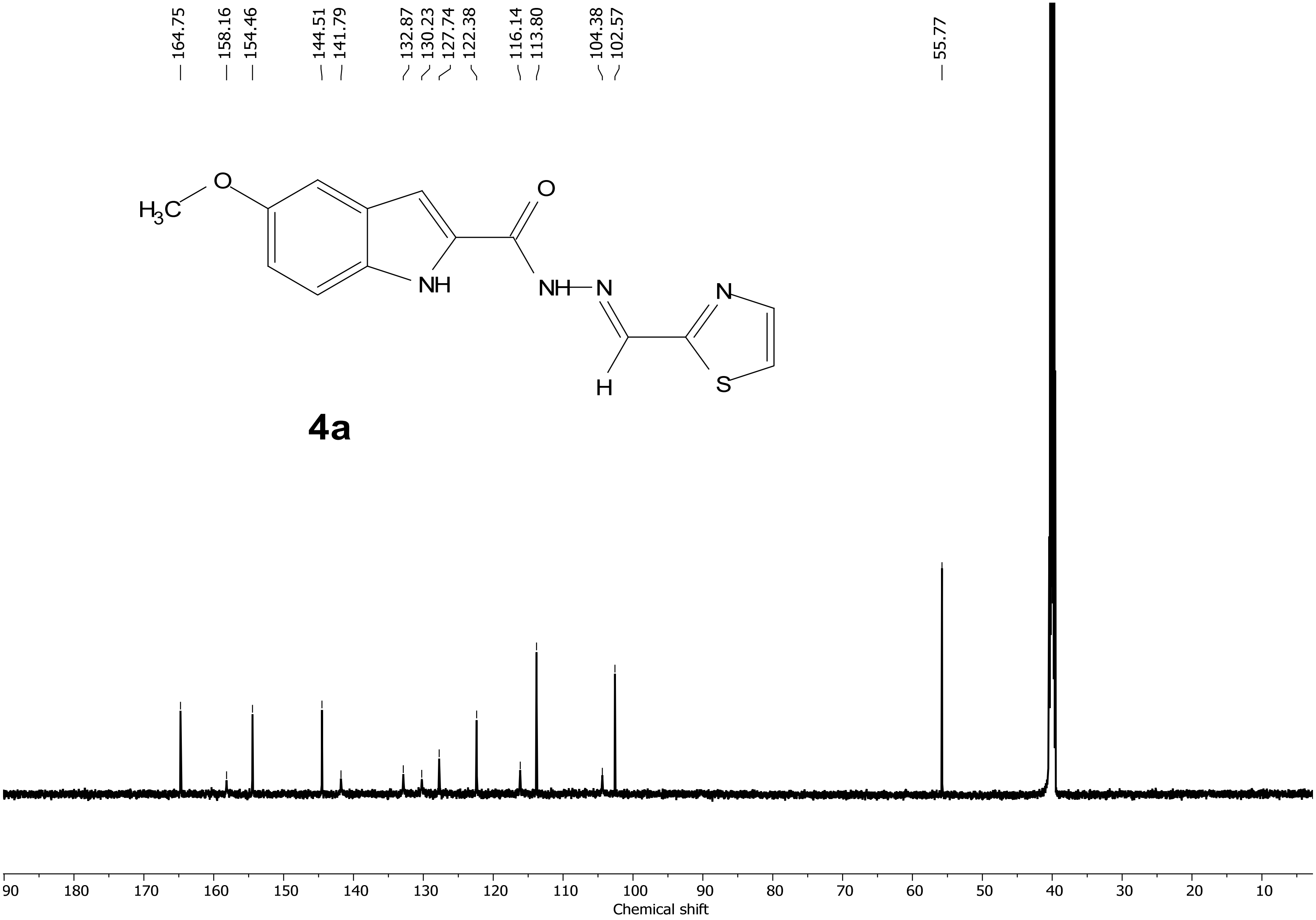

164.75
158.16
154.46
144.51
141.79
132.87
130.23
127.74
122.38
116.14
113.80
104.38
102.57
55.77
4a
90
180
170
160
150
140
130
120
110
100
90
80
70
60
50
40
30
20
10
Chemical shift

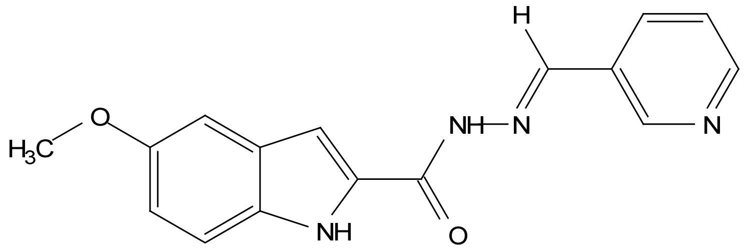

H
NH
N
N
O
H3C
NH
O

4b

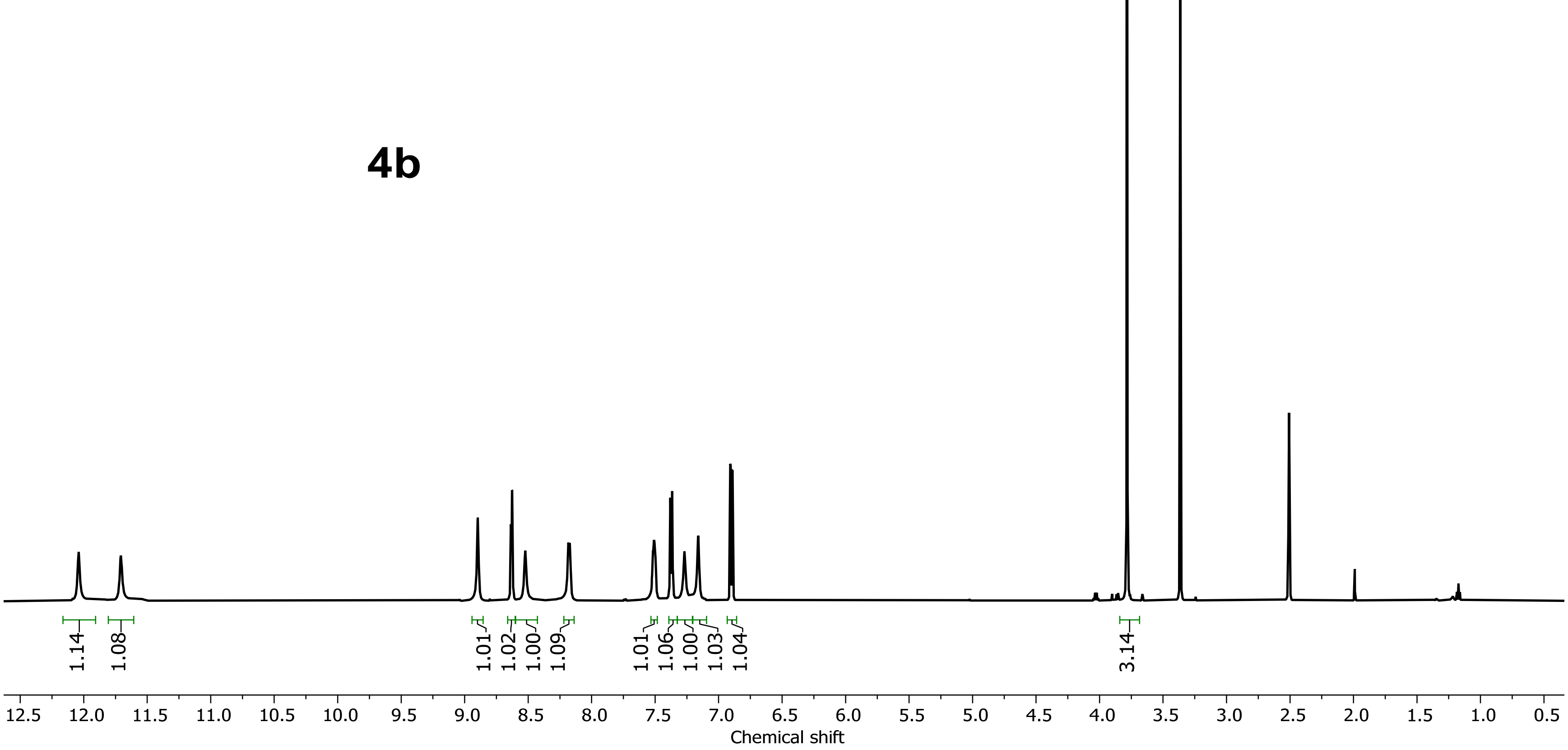

1.14
1.08
1.01
1.02
1.00
1.09
1.01
1.06
1.00
1.03
1.04
3.14
12.5 12.0 11.5 11.0 10.5 10.0 9.5 9.0 8.5 8.0 7.5 7.0 6.5 6.0 5.5 5.0 4.5 4.0 3.5 3.0 2.5 2.0 1.5 1.0 0.5
Chemical shift

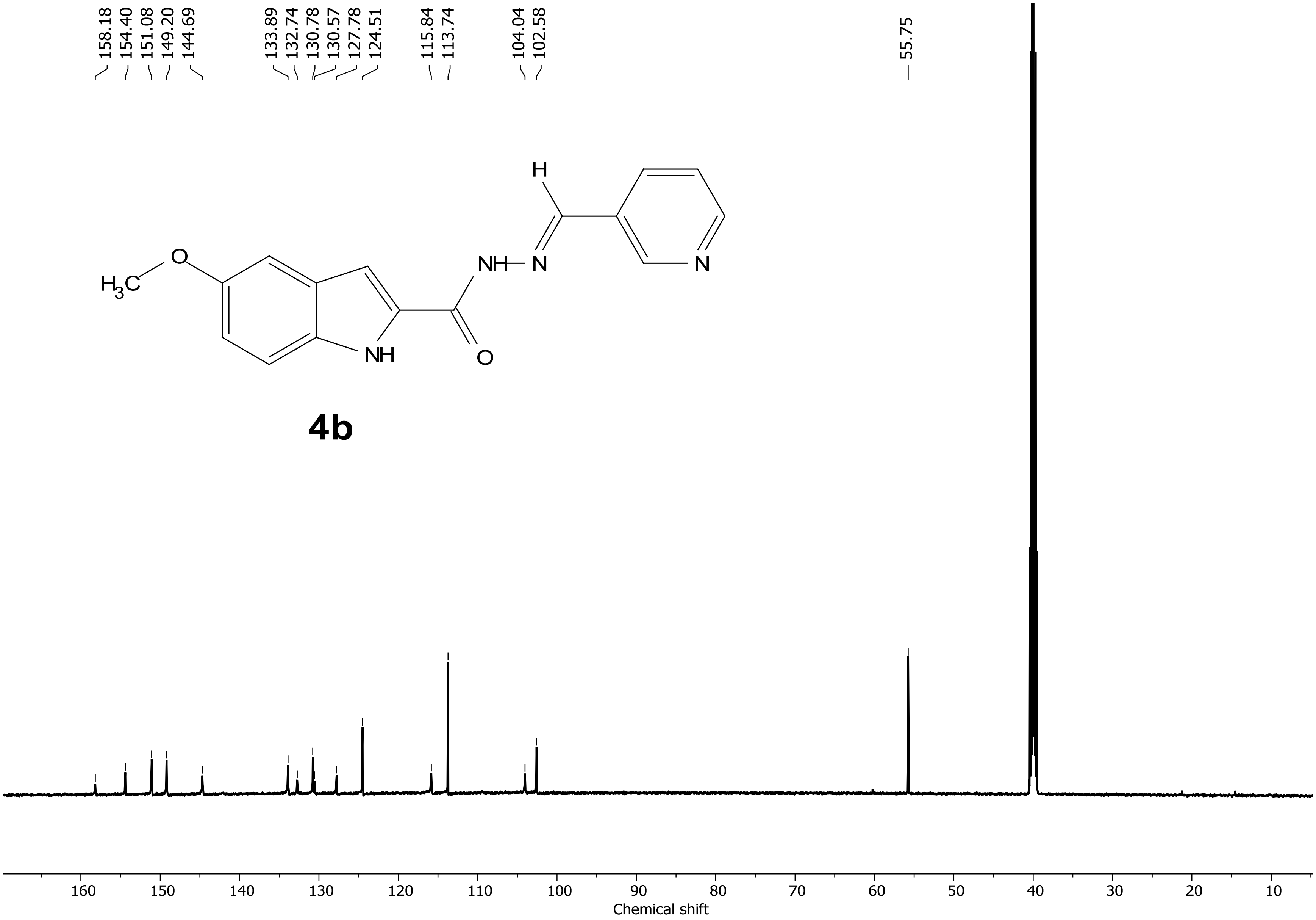

158.18
154.40
151.08
149.20
144.69
133.89
132.74
130.78
130.57
127.78
124.51
115.84
113.74
104.04
102.58
55.75
H
H_3C
O
NH
N
N
NH
O
160
150
140
130
120
110
100
90
80
70
60
50
40
30
20
10
Chemical shift

4b

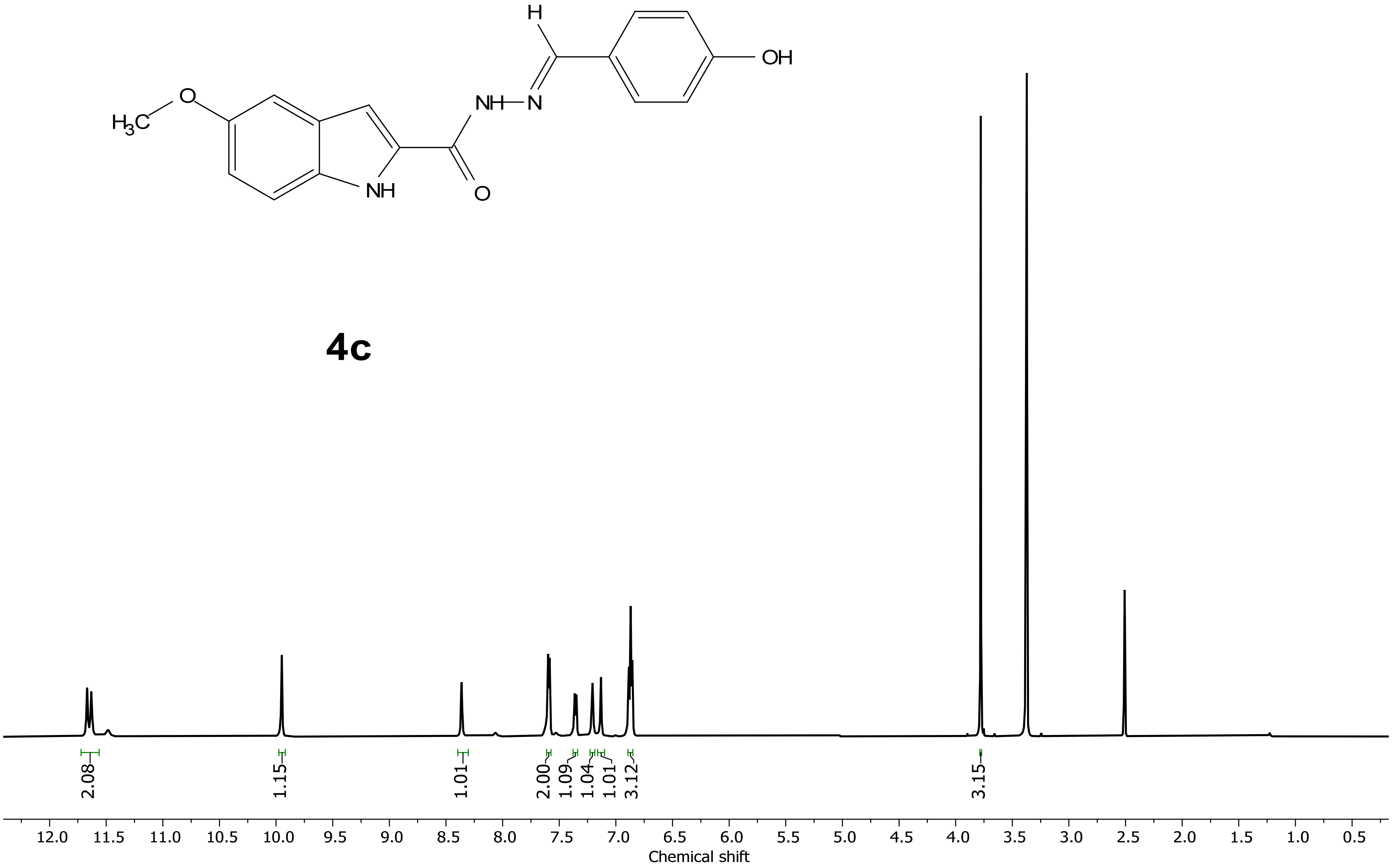
H
OH
H_3C
O
NH
N
NH
O
2.08
1.15
1.01
2.00
1.09
1.04
1.01
3.12
3.15
12.0 11.5 11.0 10.5 10.0 9.5 9.0 8.5 8.0 7.5 7.0 6.5 6.0 5.5 5.0 4.5 4.0 3.5 3.0 2.5 2.0 1.5 1.0 0.5
Chemical shift

4c

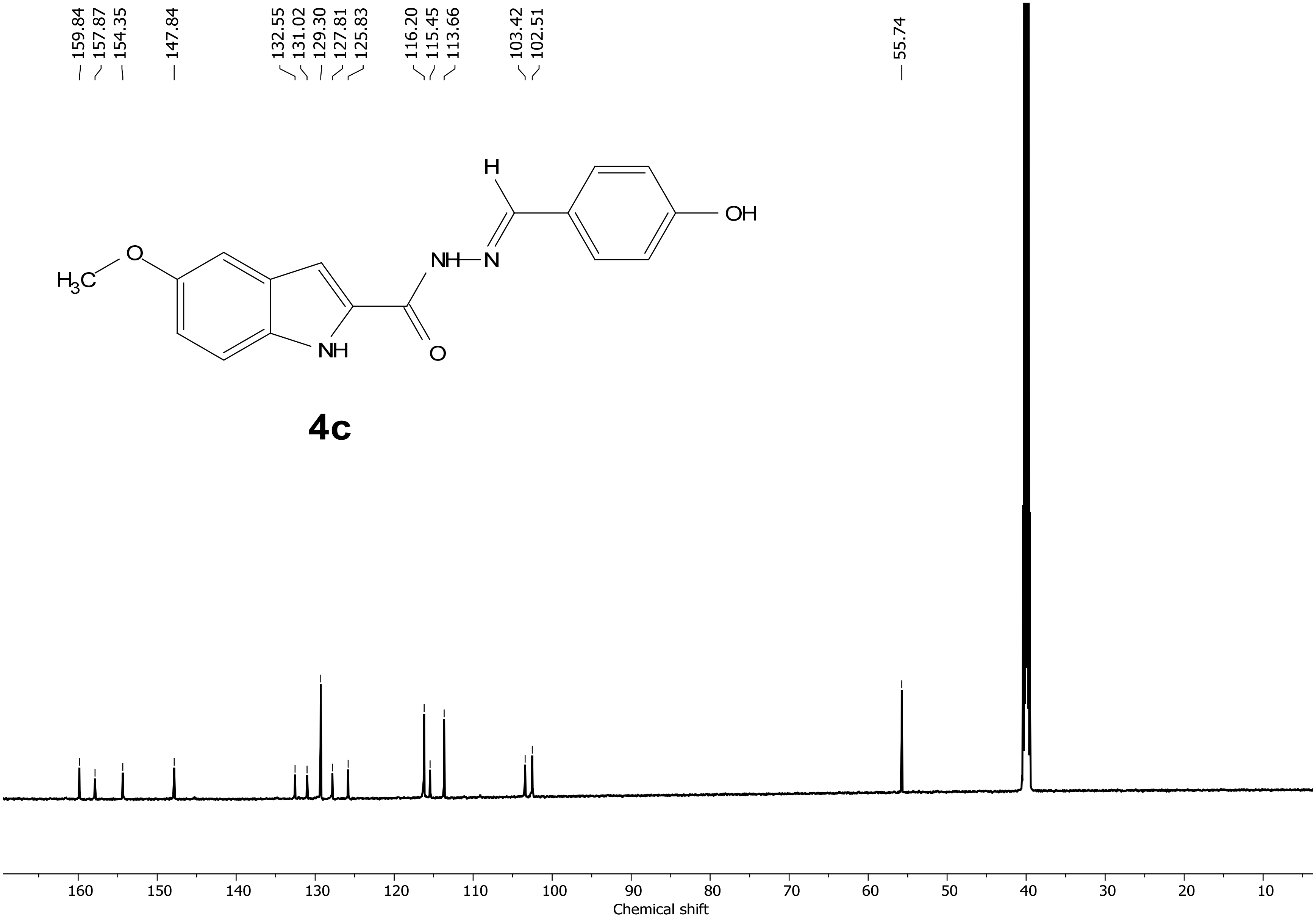
159.84
157.87
154.35
147.84
132.55
131.02
129.30
127.81
125.83
116.20
115.45
113.66
103.42
102.51
55.74
H
OH
H_3C
O
NH
N
NH
O
160
150
140
130
120
110
100
90
80
70
60
50
40
30
20
10
Chemical shift

4c

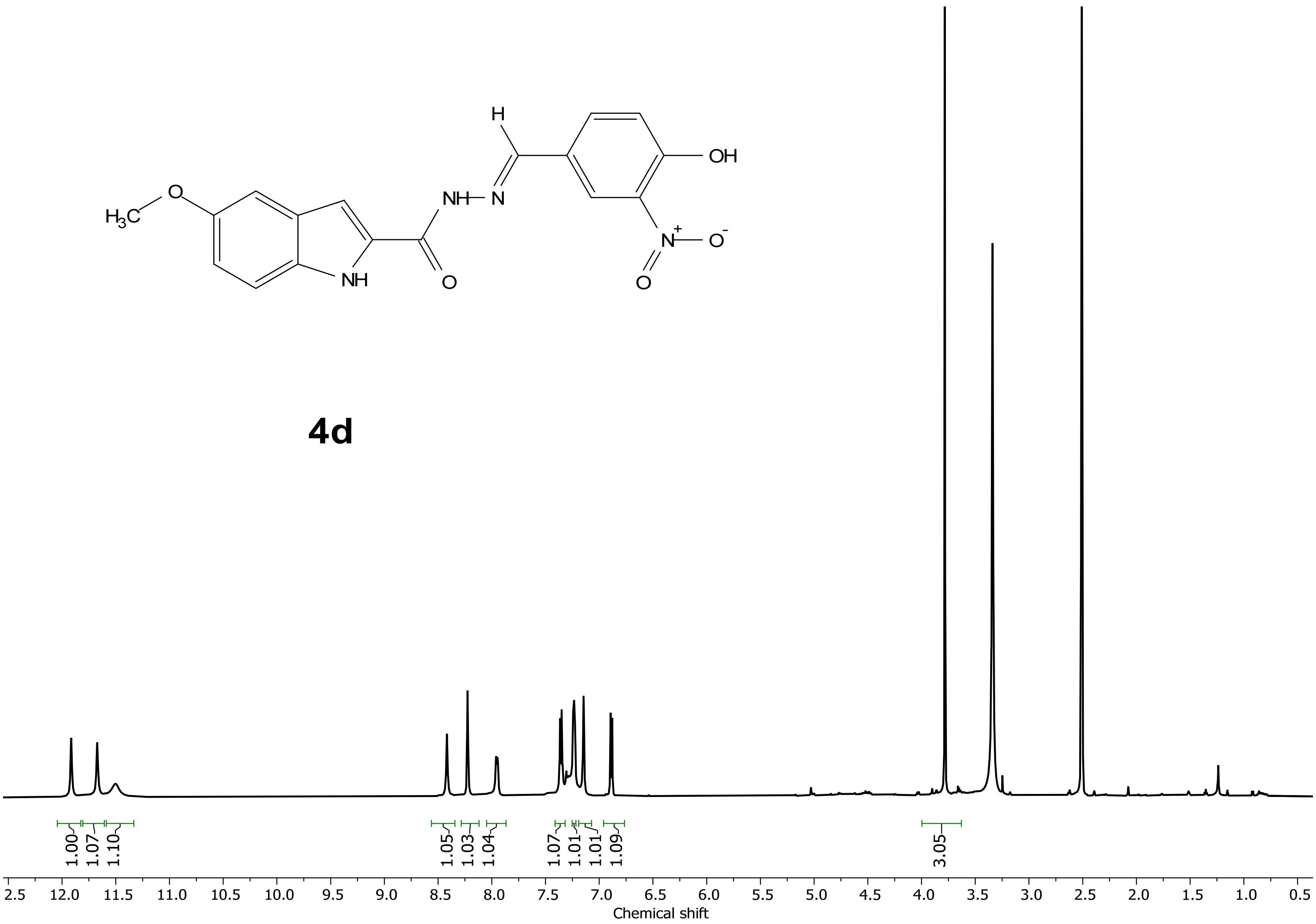
H
OH
H3C
O
NH
N
N+
O-
NH
O
O
1.00
1.07
1.10
1.05
1.03
1.04
1.07
1.01
1.01
1.09
3.05
2.5
12.0
11.5
11.0
10.5
10.0
9.5
9.0
8.5
8.0
7.5
7.0
6.5
6.0
5.5
5.0
4.5
4.0
3.5
3.0
2.5
2.0
1.5
1.0
0.5
Chemical shift

4d

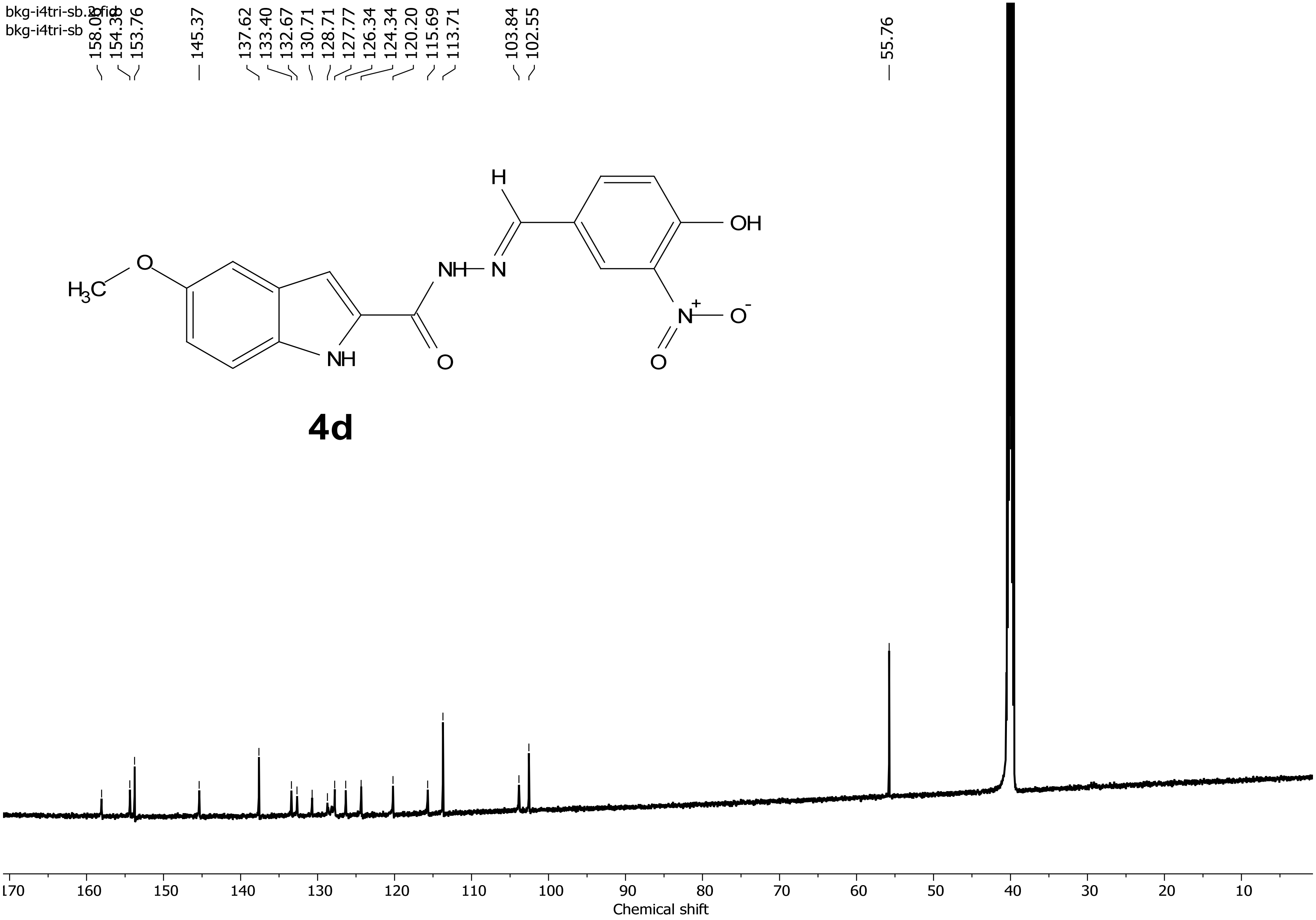
bkg-i4tri-sb.2.fid
bkg-i4tri-sb
158.02
154.38
153.76
145.37
137.62
133.40
132.67
130.71
128.71
127.77
126.34
124.34
120.20
115.69
113.71
103.84
102.55
55.76
H
OH
O
H_3C
NH
N
N^+
O^-
NH
O
O
4d
170
160
150
140
130
120
110
100
90
80
70
60
50
40
30
20
10
Chemical shift

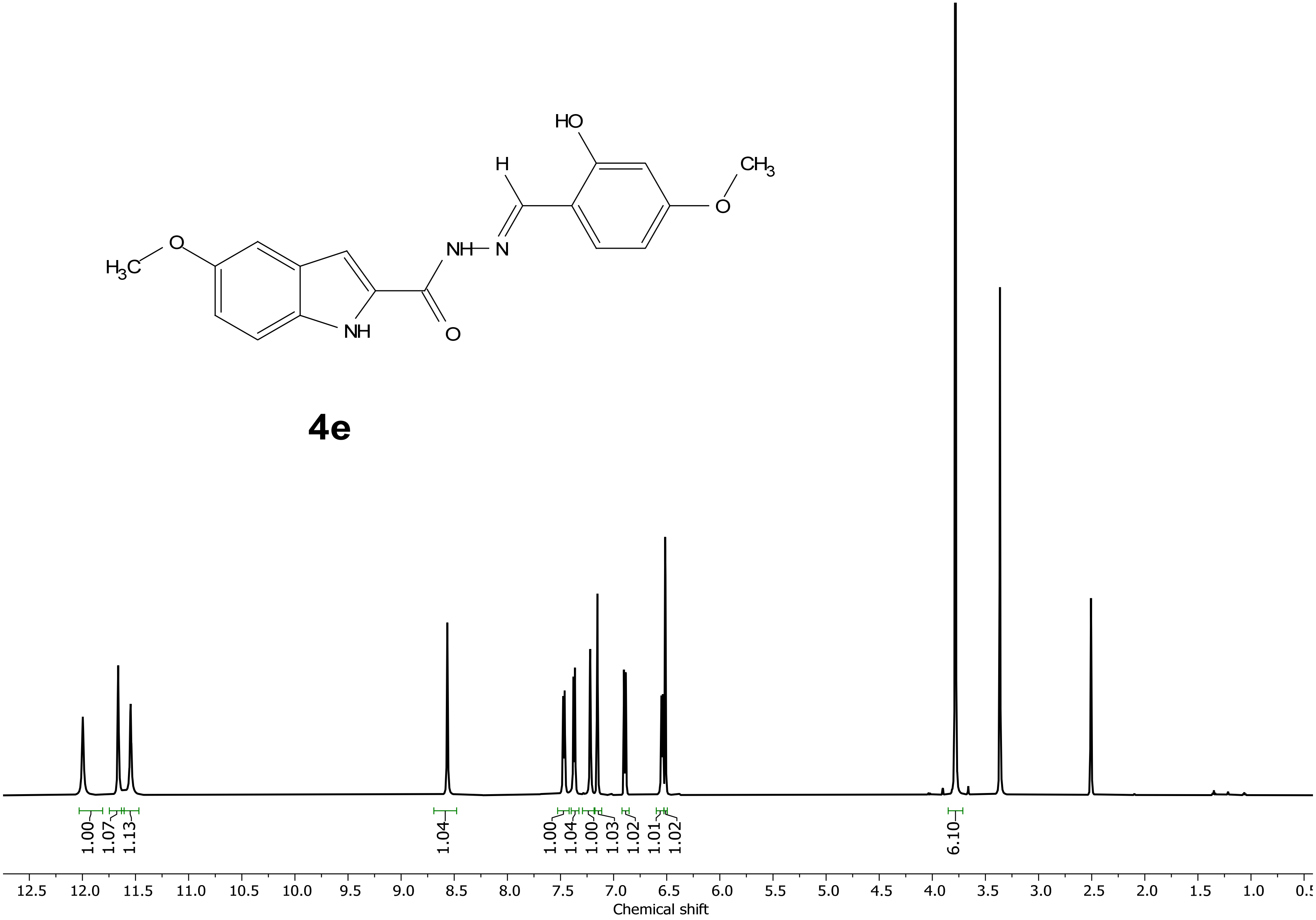
HO
H
CH3
O
H3C
O
NH
N
NH
O
4e
1.00
1.07
1.13
1.04
1.00
1.04
1.00
1.03
1.02
1.01
1.02
6.10
12.5 12.0 11.5 11.0 10.5 10.0 9.5 9.0 8.5 8.0 7.5 7.0 6.5 6.0 5.5 5.0 4.5 4.0 3.5 3.0 2.5 2.0 1.5 1.0
Chemical shift

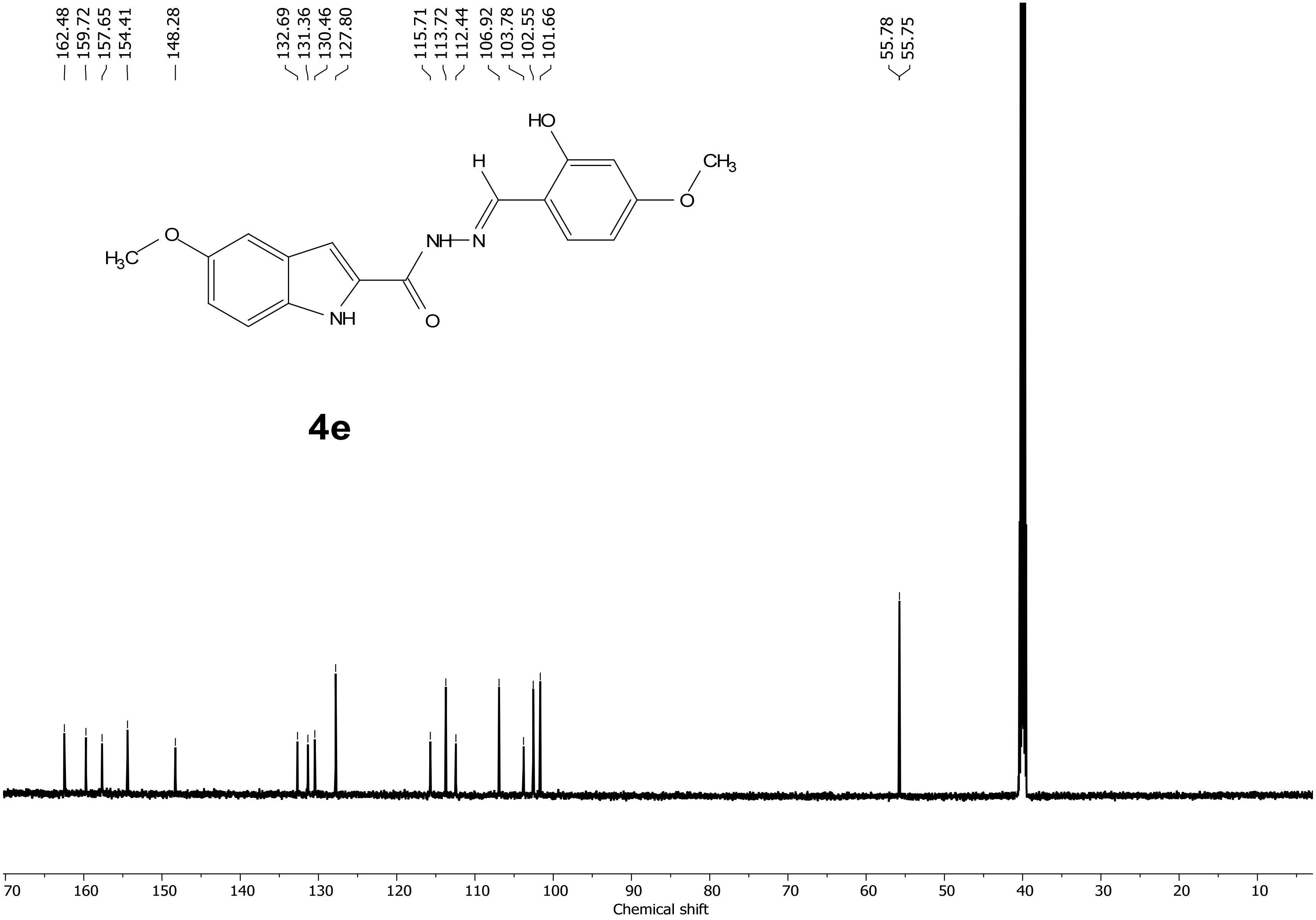

162.48
159.72
157.65
154.41
148.28
132.69
131.36
130.46
127.80
115.71
113.72
112.44
106.92
103.78
102.55
101.66
55.78
55.75
HO
H
CH3
O
NH
N
H3C
O
NH
O
Chemical shift
170
160
150
140
130
120
110
100
90
80
70
60
50
40
30
20
10

4e

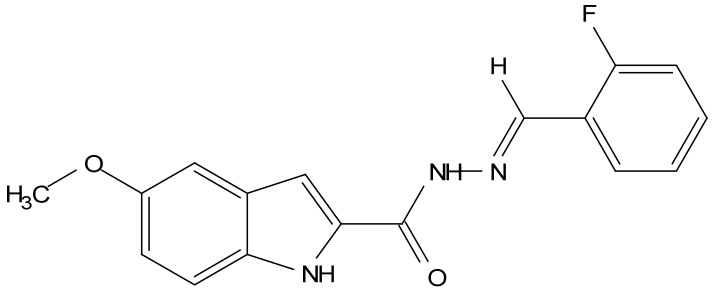

4f

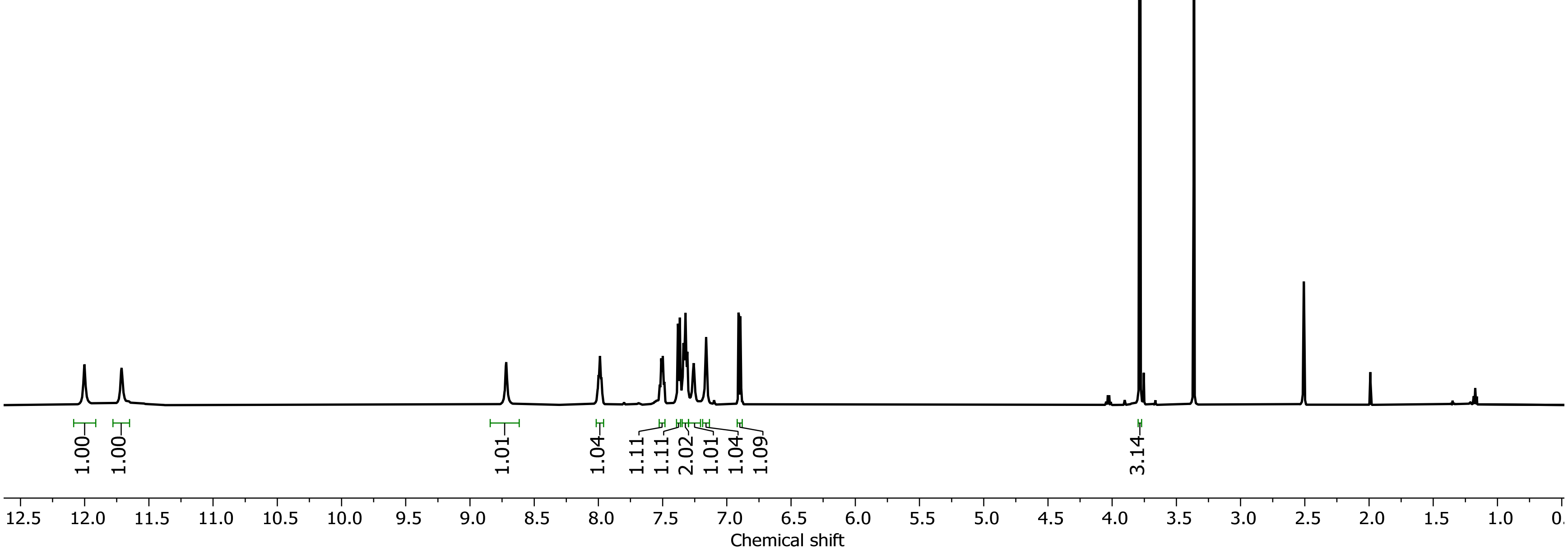

1.00
1.00
1.01
1.04
1.11
1.11
2.02
1.01
1.04
1.09
3.14
12.5
12.0
11.5
11.0
10.5
10.0
9.5
9.0
8.5
8.0
7.5
7.0
6.5
6.0
5.5
5.0
4.5
4.0
3.5
3.0
2.5
2.0
1.5
1.0
Chemical shift

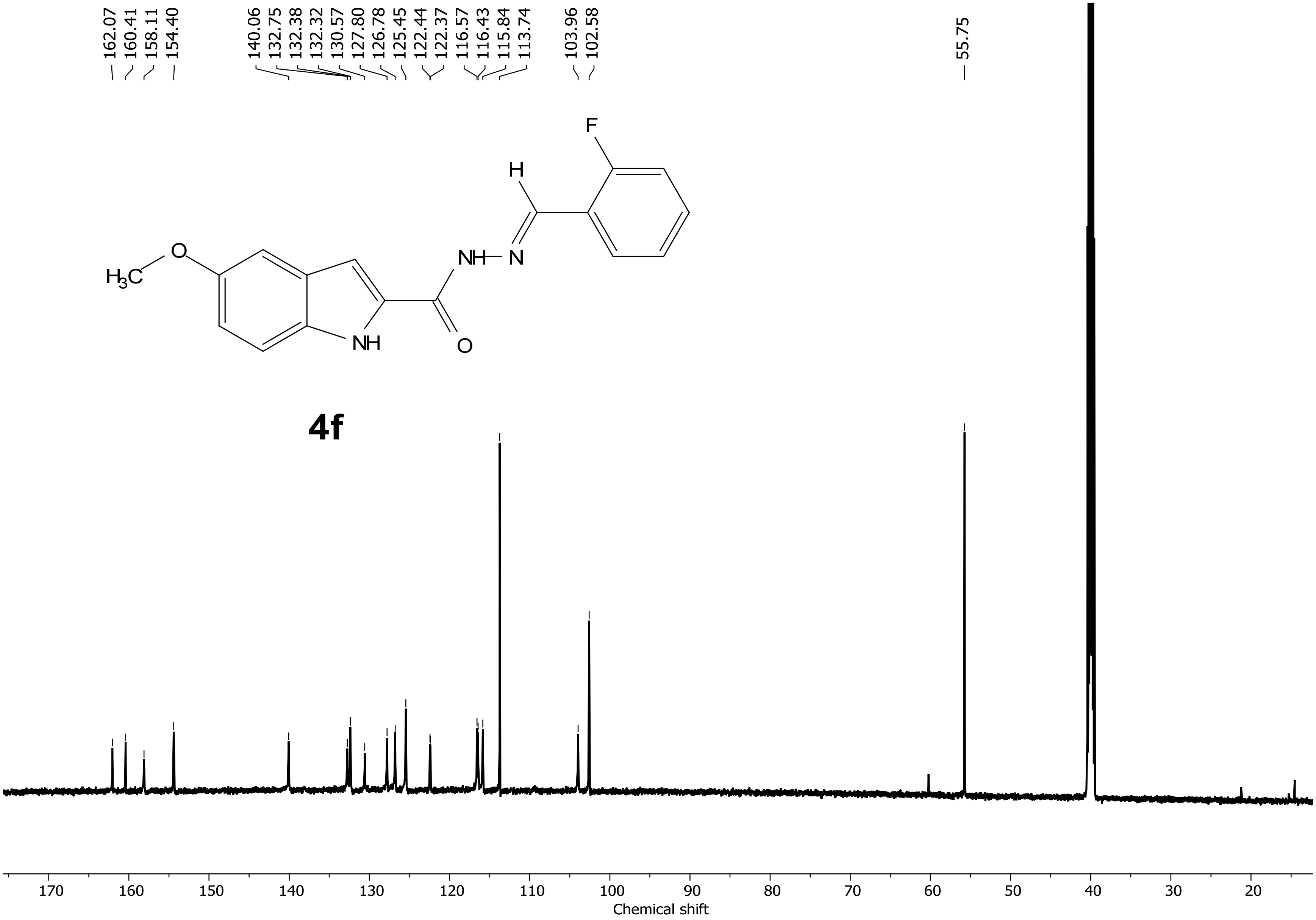
162.07
160.41
158.11
154.40
140.06
132.75
132.38
132.32
130.57
127.80
126.78
125.45
122.44
122.37
116.57
116.43
115.84
113.74
103.96
102.58
55.75
F
H
O
H_3C
NH
N
NH
O
4f
170
160
150
140
130
120
110
100
90
80
70
60
50
40
30
20
Chemical shift

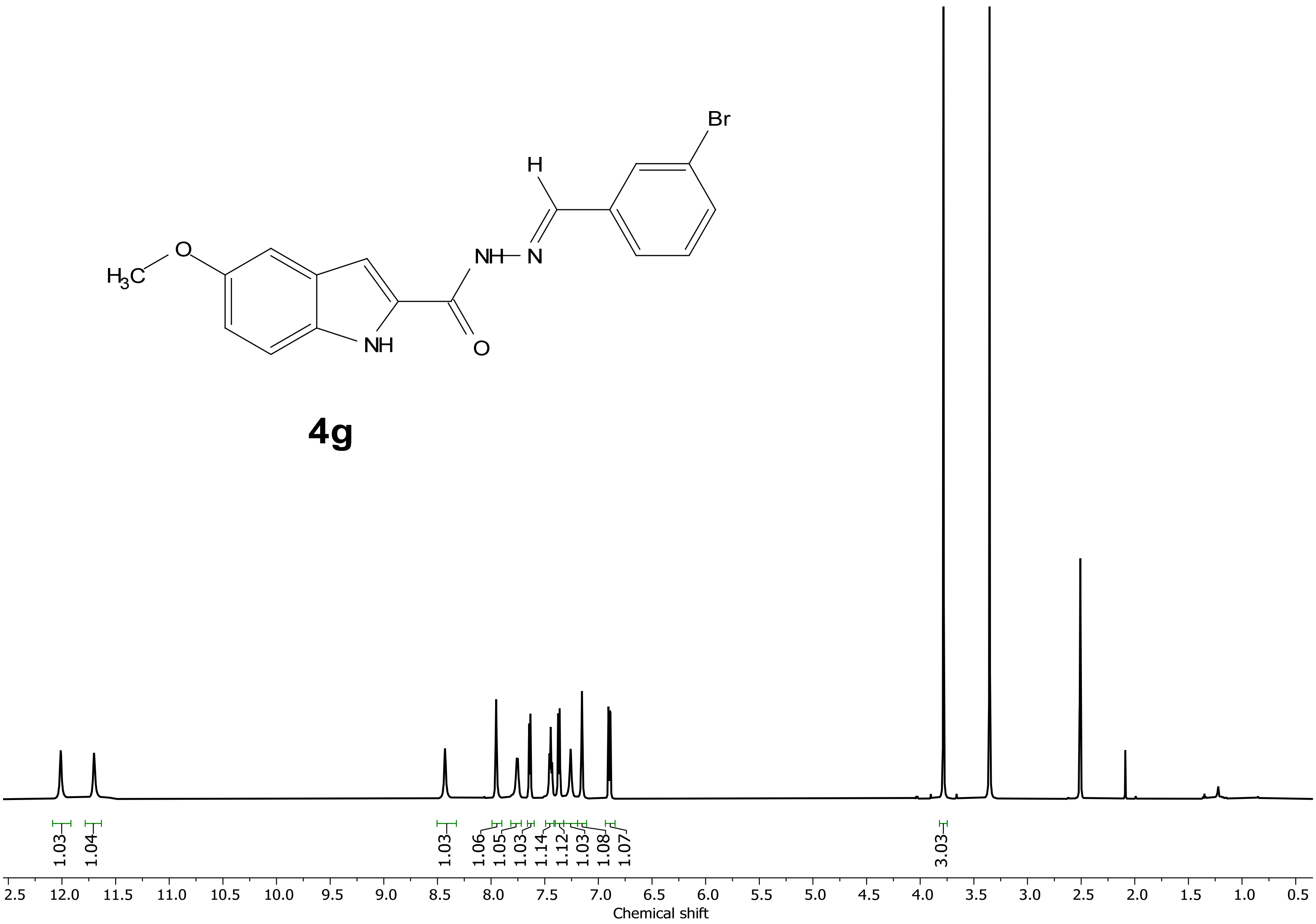
Br
H
NH
N
O
H3C
NH
O
4g
1.03
1.04
1.03
1.06
1.05
1.03
1.14
1.12
1.03
1.08
1.07
3.03
2.5 12.0 11.5 11.0 10.5 10.0 9.5 9.0 8.5 8.0 7.5 7.0 6.5 6.0 5.5 5.0 4.5 4.0 3.5 3.0 2.5 2.0 1.5 1.0 0.5
Chemical shift

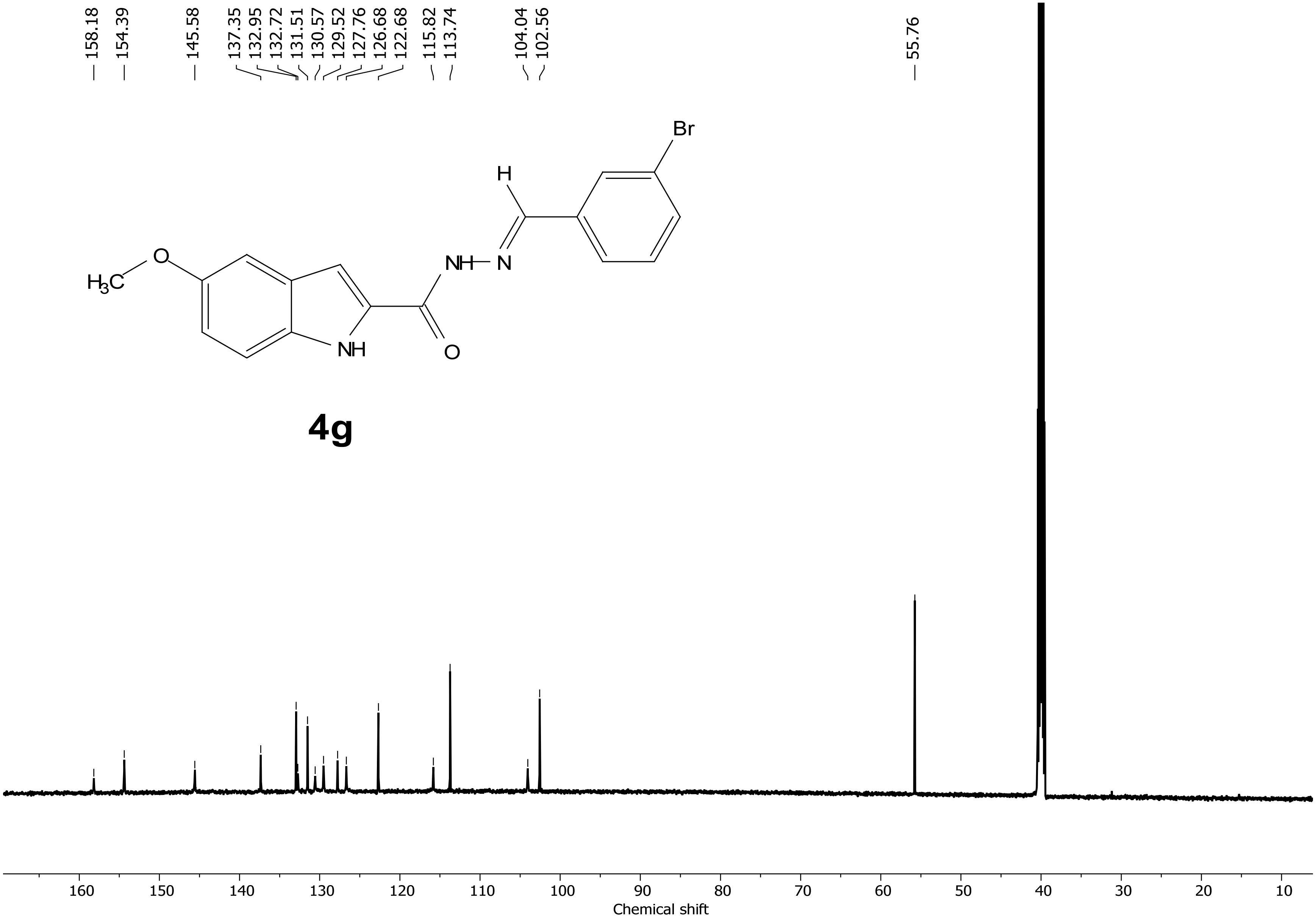
158.18
154.39
145.58
137.35
132.95
132.72
131.51
130.57
129.52
127.76
126.68
122.68
115.82
113.74
104.04
102.56
55.76
Br
H
O
H_3C
NH
N
NH
O
160
150
140
130
120
110
100
90
80
70
60
50
40
30
20
10
Chemical shift

4g

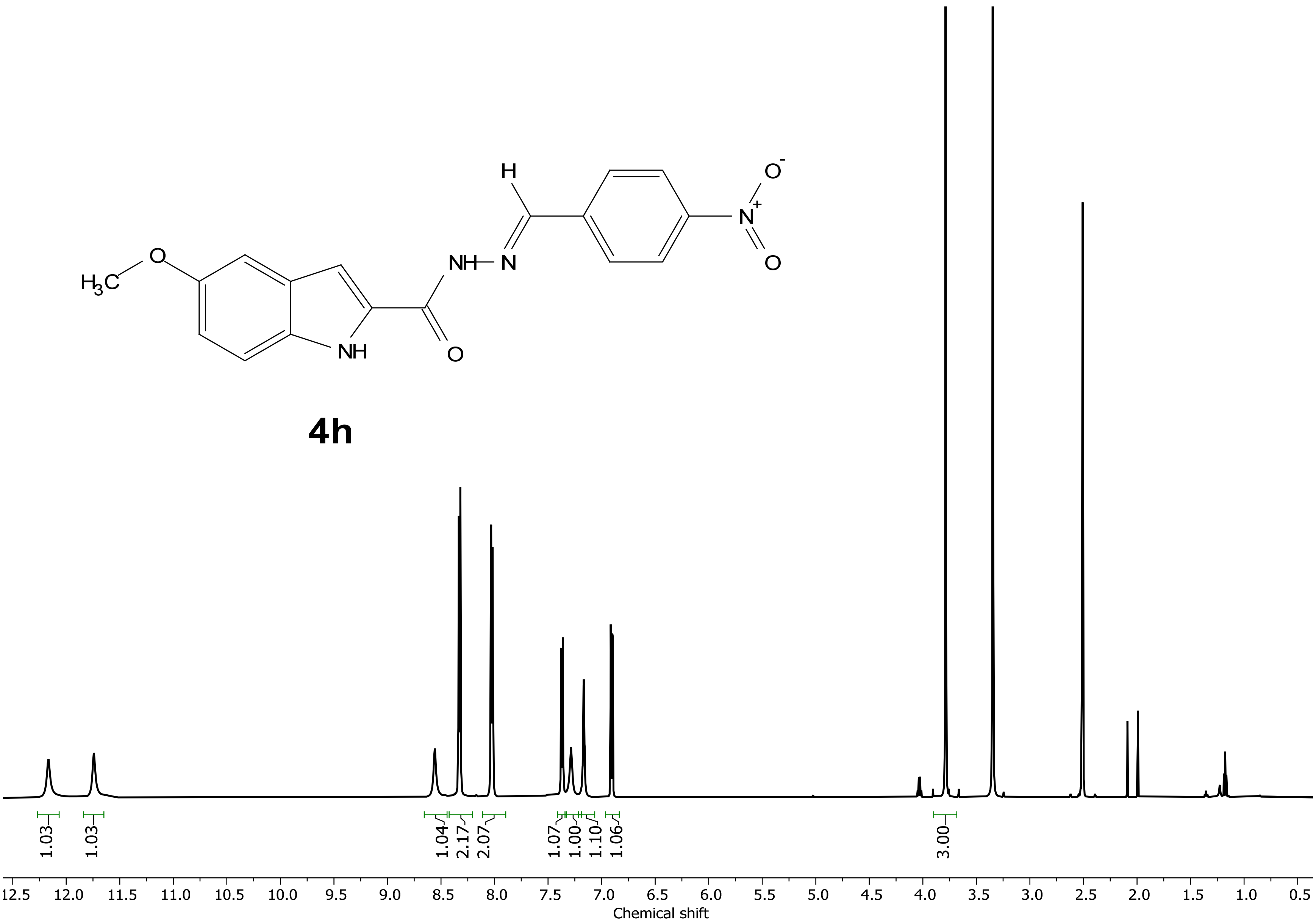
H
O
H_3C
O
NH
N
N
O
NH
O
1.03
1.03
1.04
2.17
2.07
1.07
1.00
1.10
1.06
3.00
12.5 12.0 11.5 11.0 10.5 10.0 9.5 9.0 8.5 8.0 7.5 7.0 6.5 6.0 5.5 5.0 4.5 4.0 3.5 3.0 2.5 2.0 1.5 1.0 0.5
Chemical shift

4h

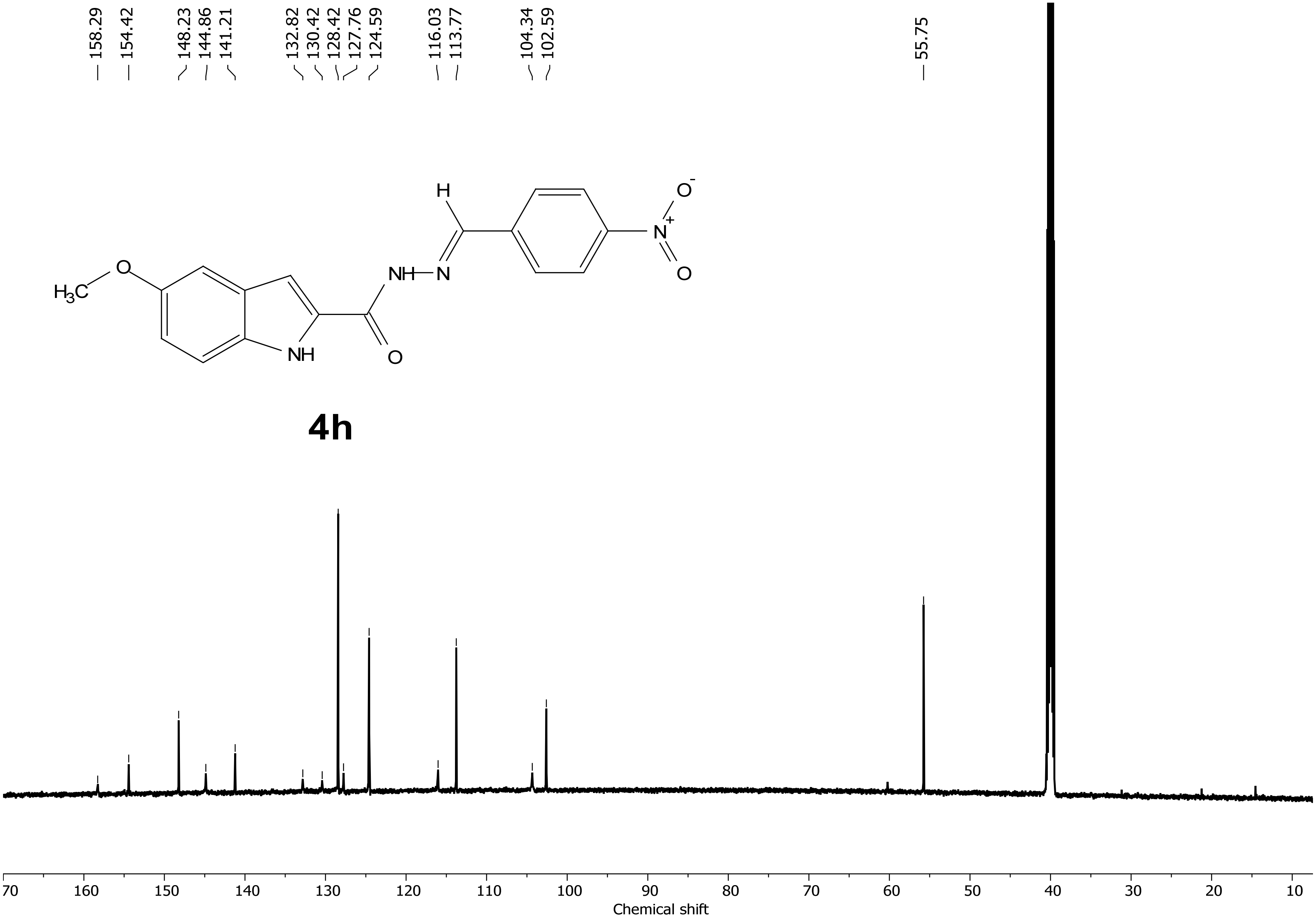
158.29
154.42
148.23
144.86
141.21
132.82
130.42
128.42
127.76
124.59
116.03
113.77
104.34
102.59
55.75
H₃C O
NH
NH N
H
O
N+
O-
O
4h
70
160
150
140
130
120
110
100
90
80
70
60
50
40
30
20
10
Chemical shift

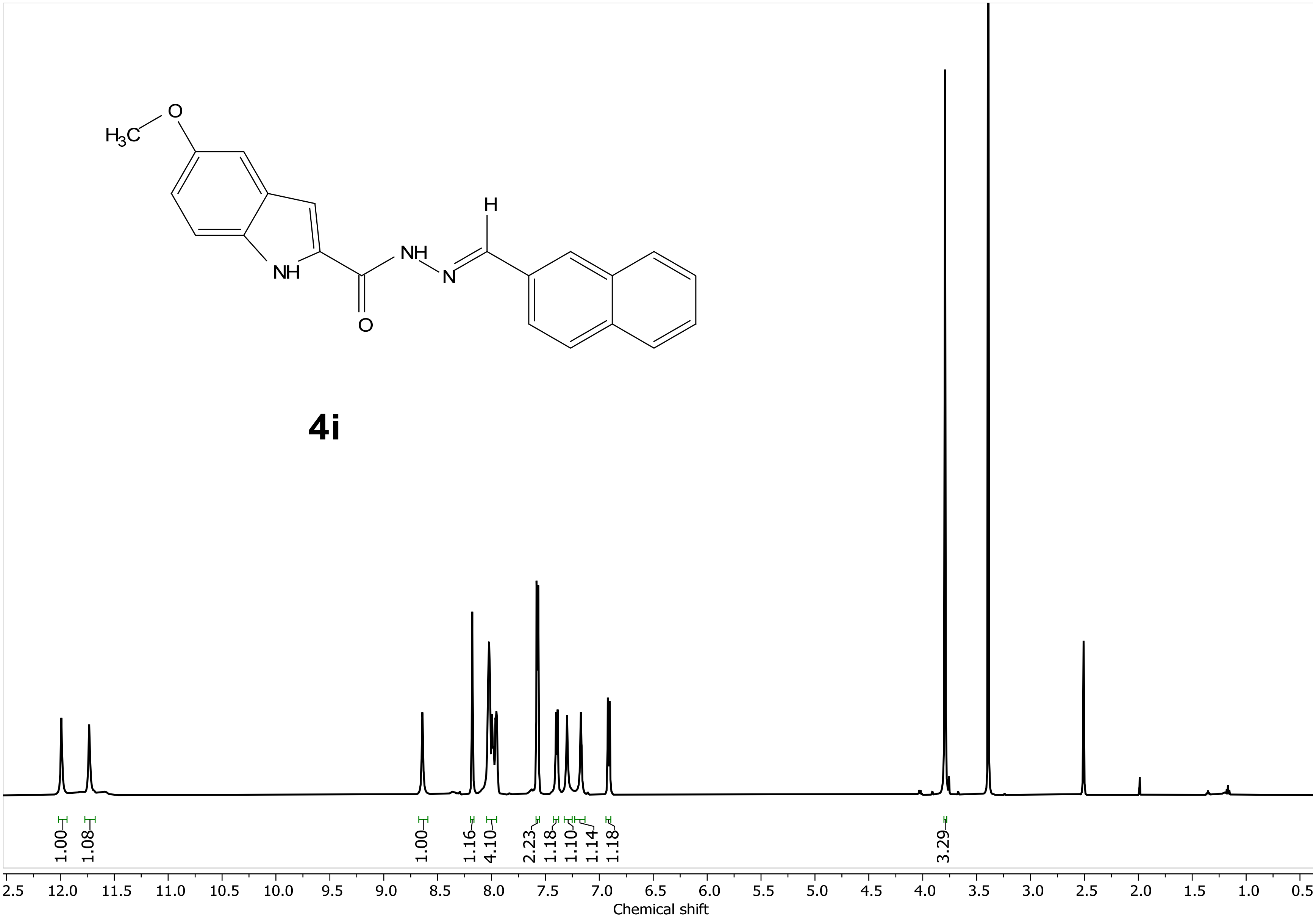

H3C
O
NH
NH
N
H
O
4i
1.00
1.08
1.00
1.16
4.10
2.23
1.18
1.10
1.14
1.18
3.29
12.0 11.5 11.0 10.5 10.0 9.5 9.0 8.5 8.0 7.5 7.0 6.5 6.0 5.5 5.0 4.5 4.0 3.5 3.0 2.5 2.0 1.5 1.0 0.5
Chemical shift

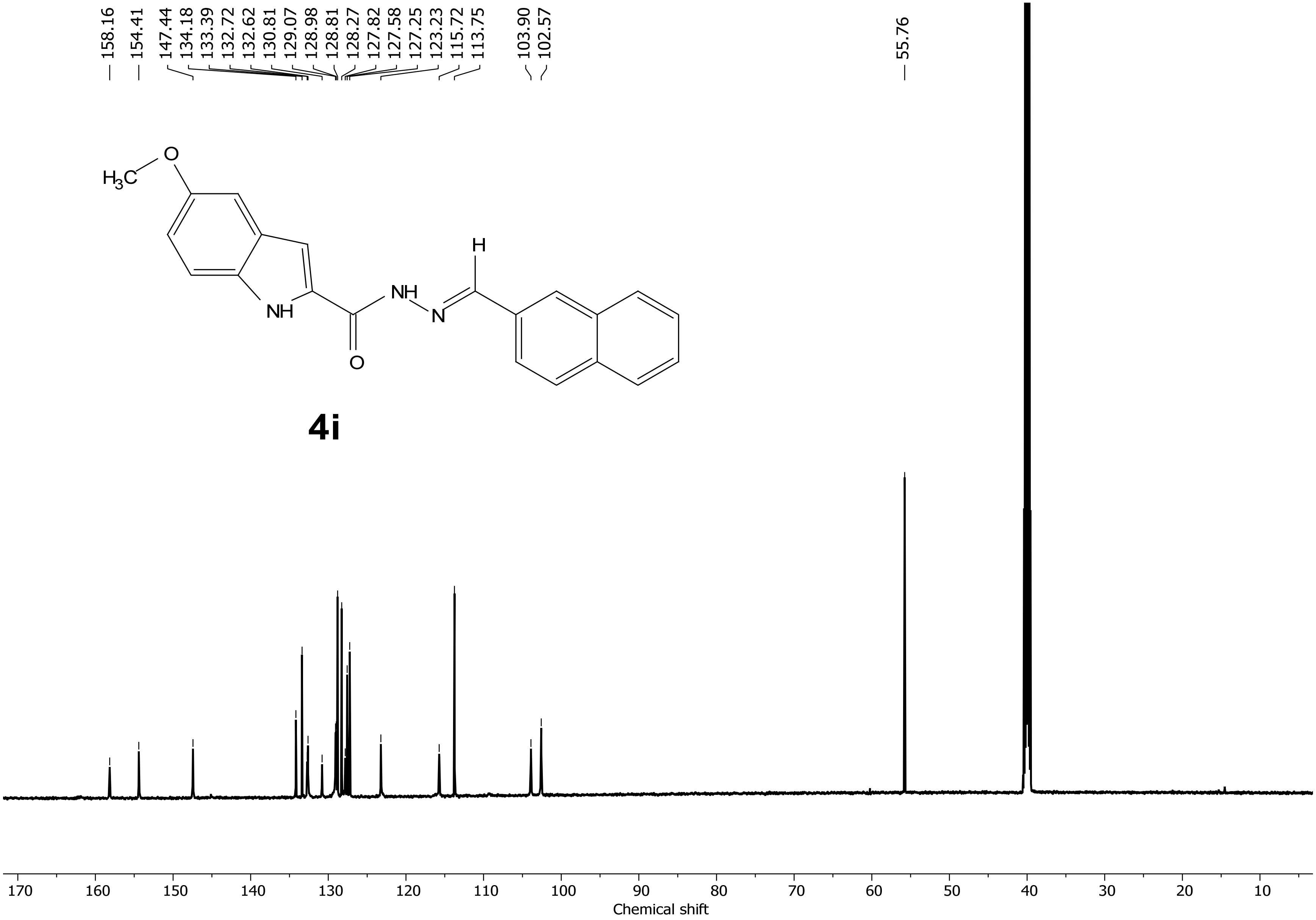
158.16
154.41
147.44
134.18
133.39
132.72
132.62
130.81
129.07
128.98
128.81
128.27
127.82
127.58
127.25
123.23
115.72
113.75
103.90
102.57
55.76
H3C
O
NH
NH
N
H
O
4i
170 160 150 140 130 120 110 100 90 80 70 60 50 40 30 20 10
Chemical shift

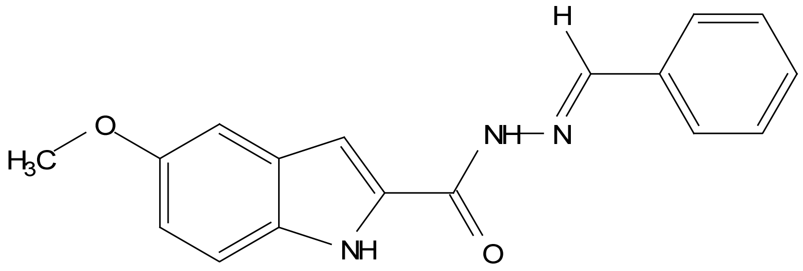

4j

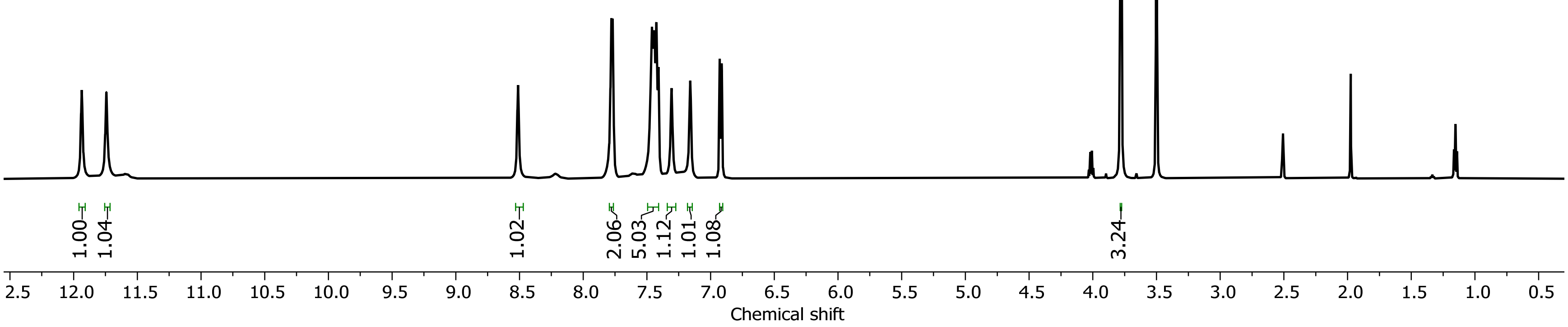

1.00
1.04
1.02
2.06
5.03
1.12
1.01
1.08
3.24
2.5
12.0
11.5
11.0
10.5
10.0
9.5
9.0
8.5
8.0
7.5
7.0
6.5
6.0
5.5
5.0
4.5
4.0
3.5
3.0
2.5
2.0
1.5
1.0
0.5
Chemical shift

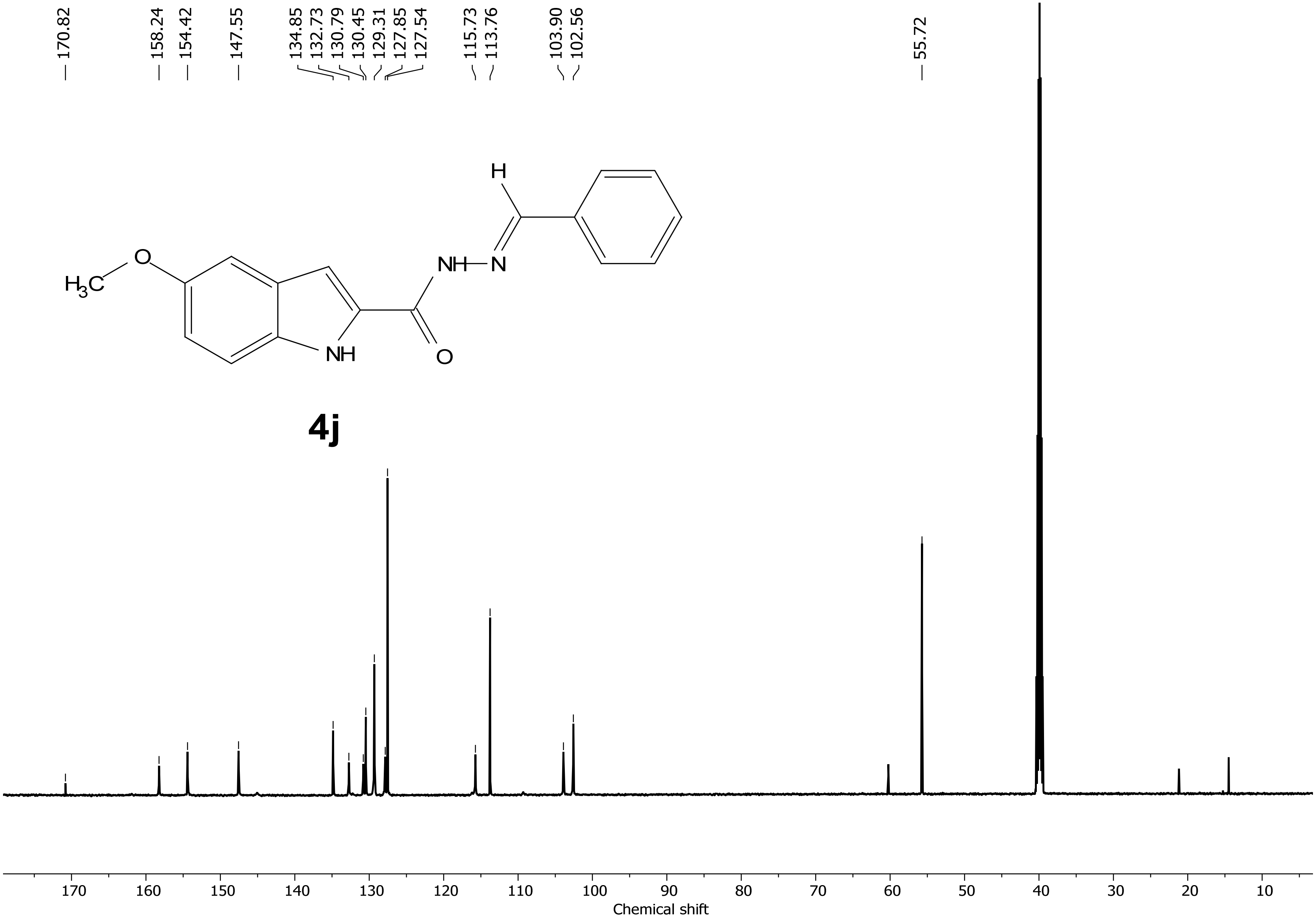

170.82
158.24
154.42
147.55
134.85
132.73
130.79
130.45
129.31
127.85
127.54
115.73
113.76
103.90
102.56
55.72
H3C
O
NH
NH
N
H
O
4j
170
160
150
140
130
120
110
100
90
80
70
60
50
40
30
20
10
Chemical shift

8. Mass Spectra

4a

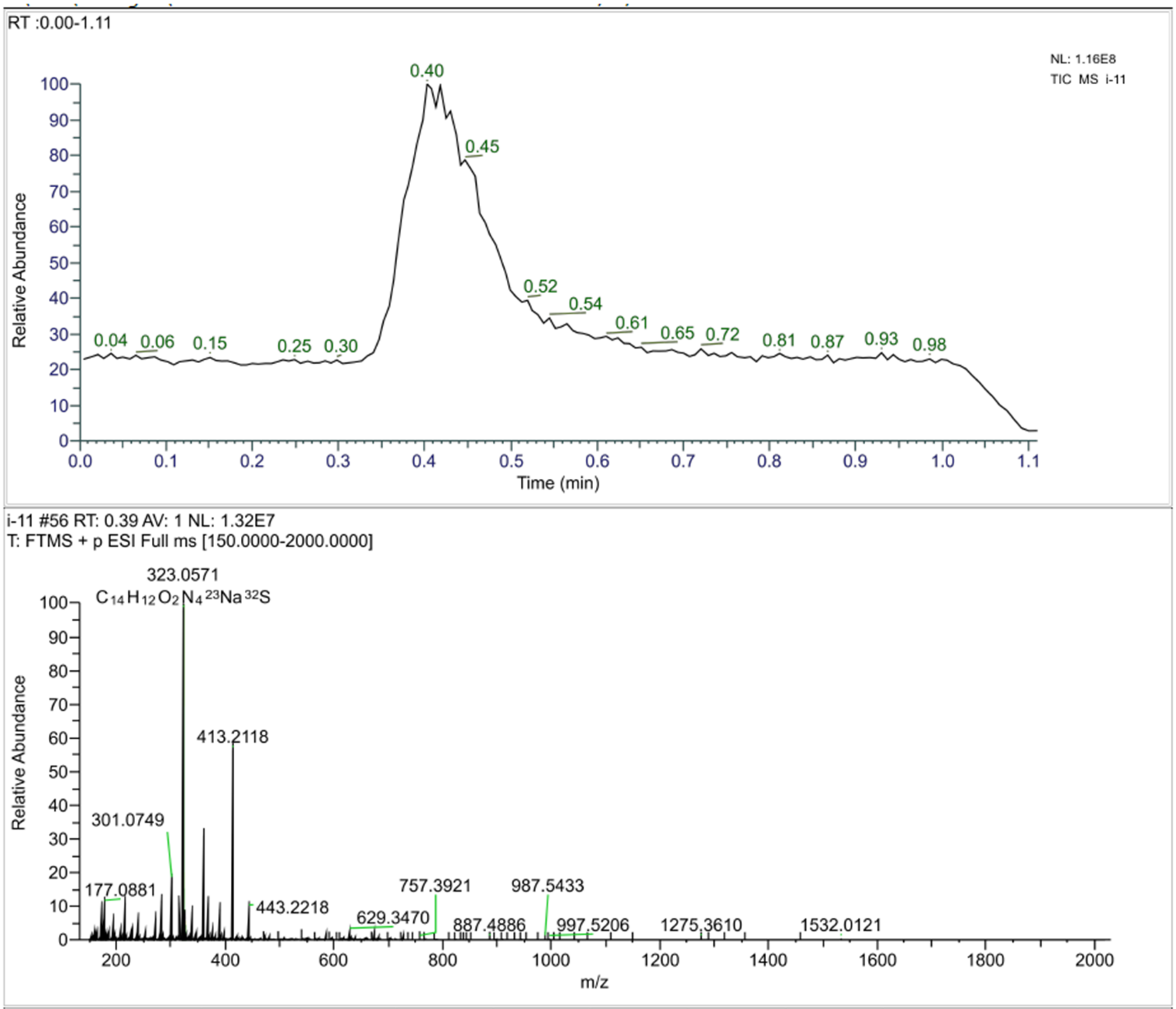

Display F...	S Fit	RDB	Delta [m...	Theo. mass	Rank	Combine...	# Matche...	# Missed...	MS Cov. [...	Pattern C...	MSMS M...
$C_{14}H_{12}O_2N_4{}^{23}Na^{32}S$	76.2440931213142	10.50	-0.25	323.05732	1	92.88	5	0	93.8	100	(Collection)

4b

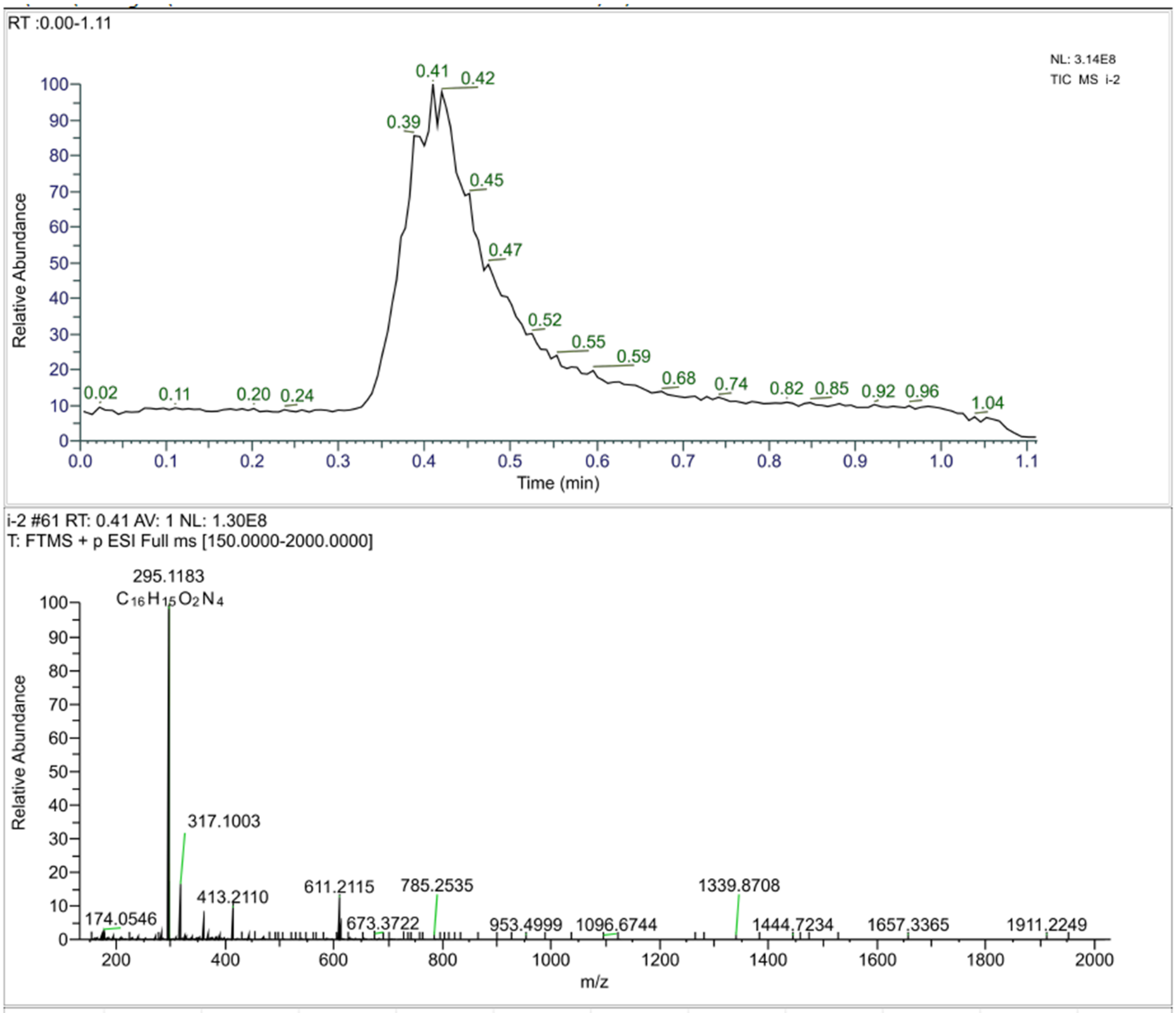

Display F...	S Fit	RDB	Delta [m...	Theo. mass	Rank	Combine...	# Matche...	# Missed...	MS Cov. [...	Pattern C...	MSMS M...
$C_{16}H_{15}O_2N_4$	78.895561 1363881	11.50	-0.70	295.11895	1	98.46	4	0	99.54	100	(Collection)

4c

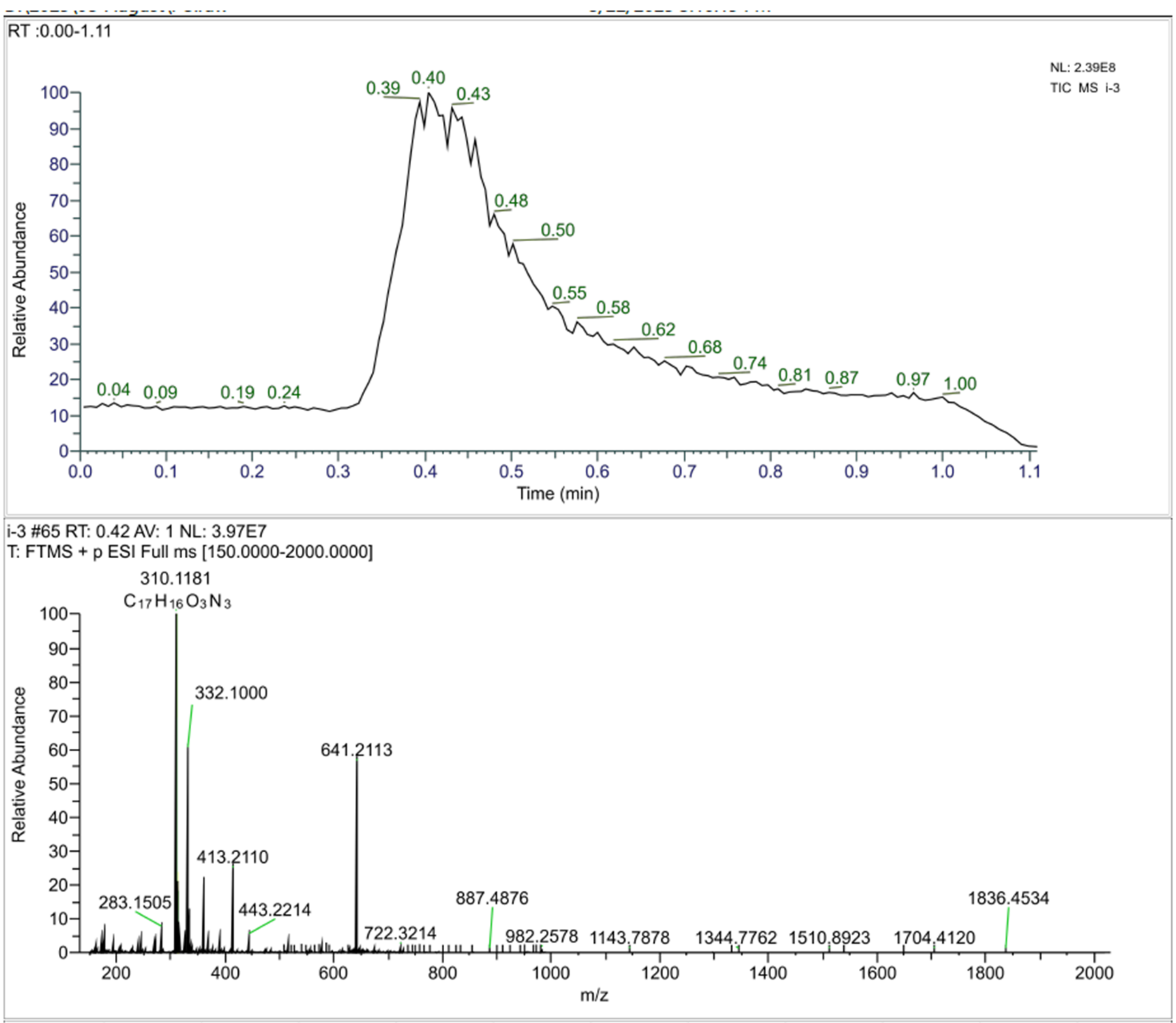

Display F...	S Fit	RDB	Delta [m...	Theo. mass	Rank	Combine...	# Matche...	# Missed...	MS Cov. [...	Pattern C...	MSMS M...
$C_{17}H_{16}O_3N_3$	44.6464122810167	11.50	-0.55	310.11862	1	96.58	4	0	99.46	99.86	(Collection)

4d

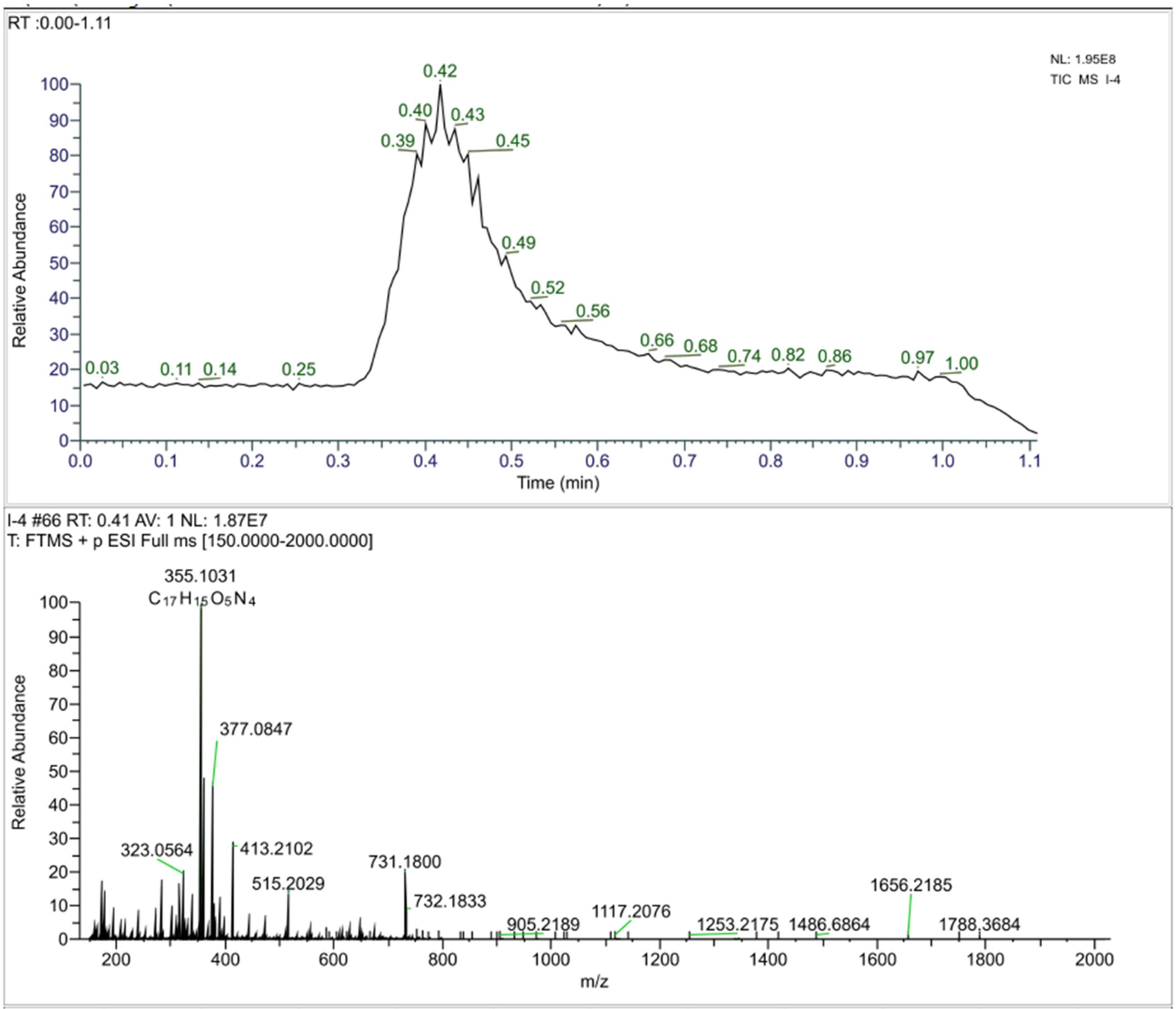

Display F...	S Fit	RDB	Delta [m...	Theo. mass	Rank	Combine...	# Matche...	# Missed...	MS Cov. [...	Pattern C...	MSMS M...
$C_{17}H_{15}O_5N_4$	47.955602727168	12.50	-0.61	355.10370	1	95.55	4	0	98.2	99.79	(Collection)

4e

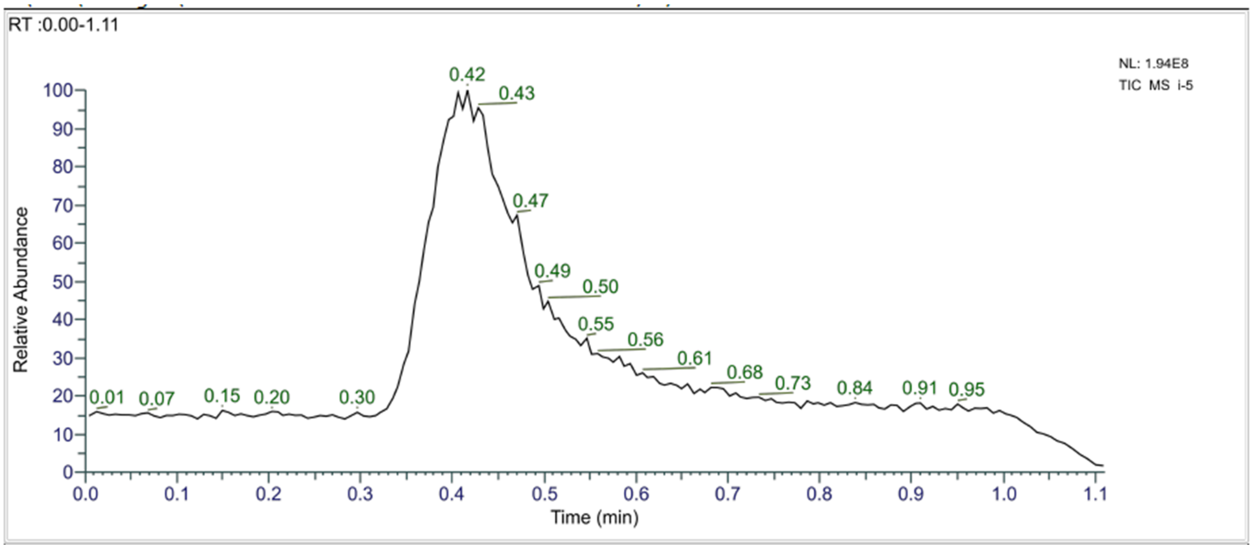

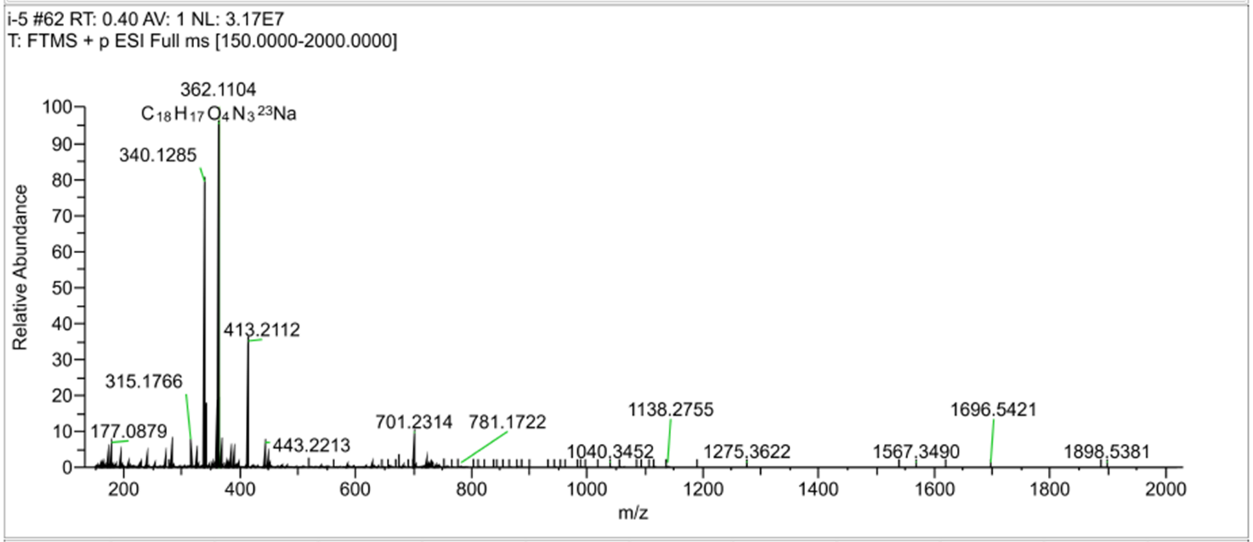

Display F...	S Fit	RDB	Delta [m...	Theo. mass	Rank	Combine...	# Matche...	# Missed...	MS Cov. [...	Pattern C...	MSMS M...
$C_{18}H_{17}O_4N_3{}^{23}Na$	76.021905 3509641	11.50	-0.78	362.11113	1	98.42	4	0	99.66	100	(Collection)

4f

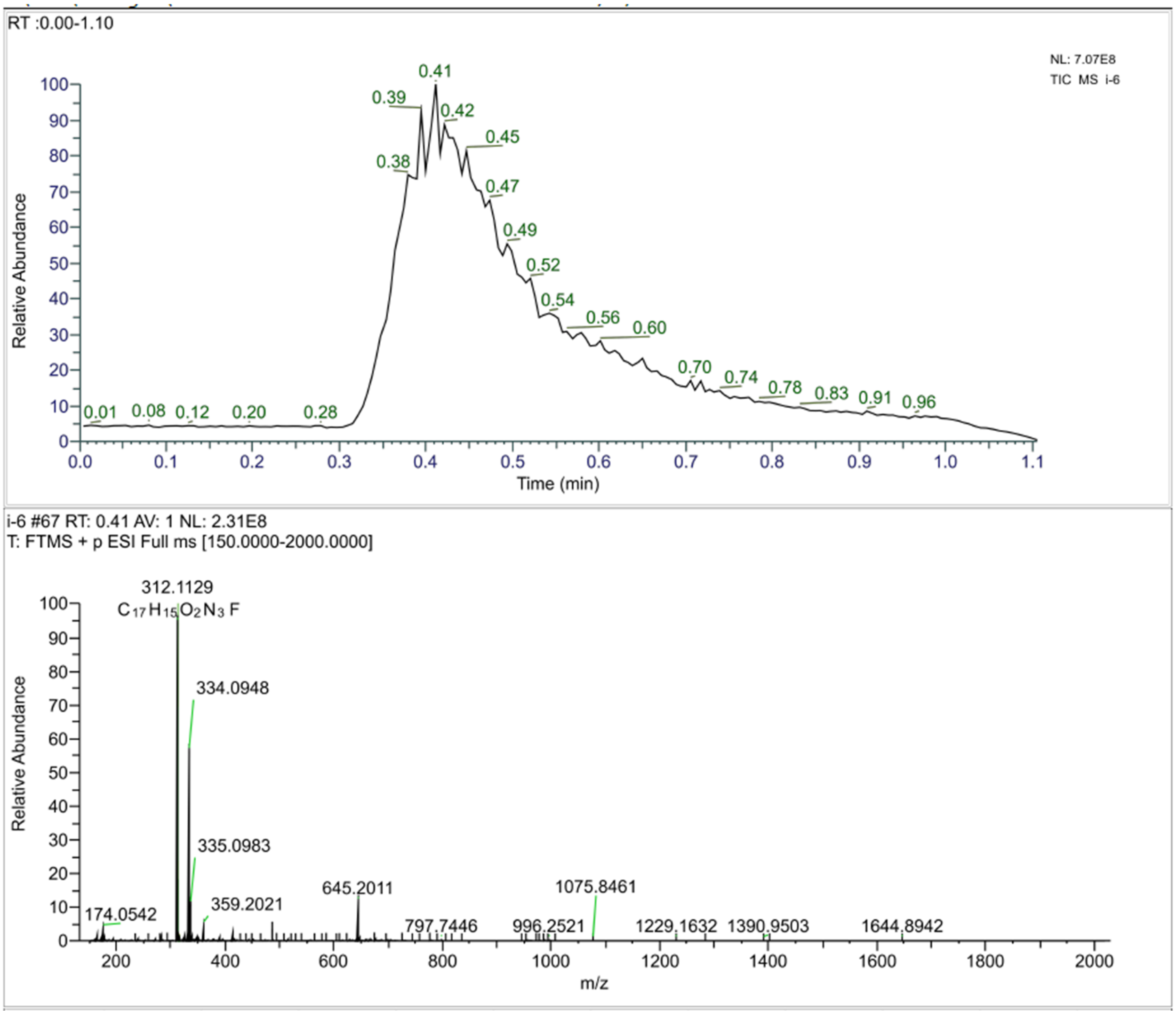

Display F...	S Fit	RDB	Delta [m...	Theo. mass	Rank	Combine...	# Matche...	# Missed...	MS Cov. [...	Pattern C...	MSMS M...
$C_{17}H_{15}O_2N_3F$	71.7950955459627	11.50	-1.40	312.11428	1	97.94	4	0	99.4	100	(Collection)

4g

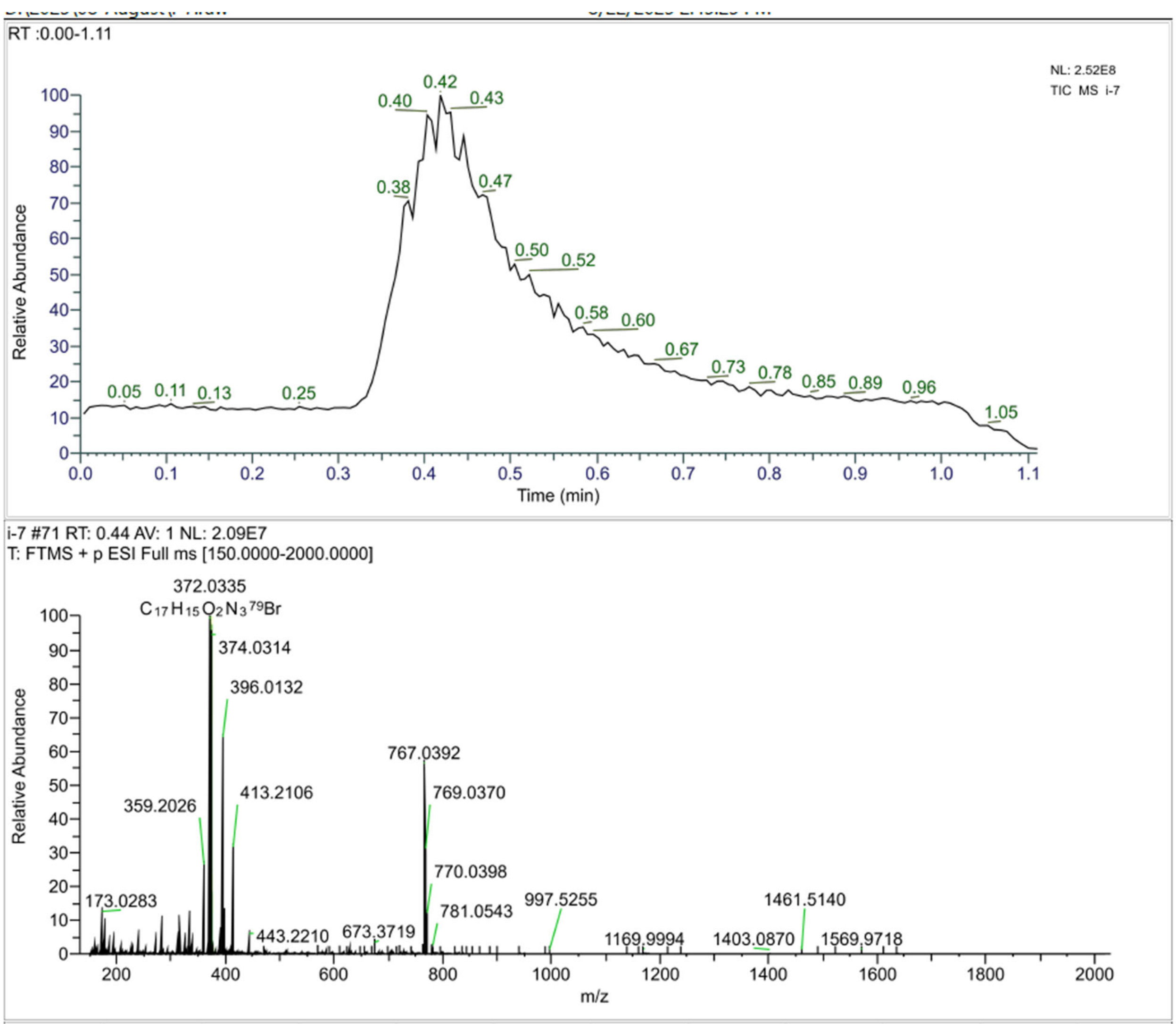

Display F...	S Fit	RDB	Delta [m...	Theo. mass	Rank	Combine...	# Matche...	# Missed...	MS Cov. [...	Pattern C...	MSMS M...
$C_{17}H_{15}O_2N_3{}^{79}Br$	73.932997 1680894	11.50	-0.71	372.03422	1	98.29	6	0	99.64	100	(Collection)

4h

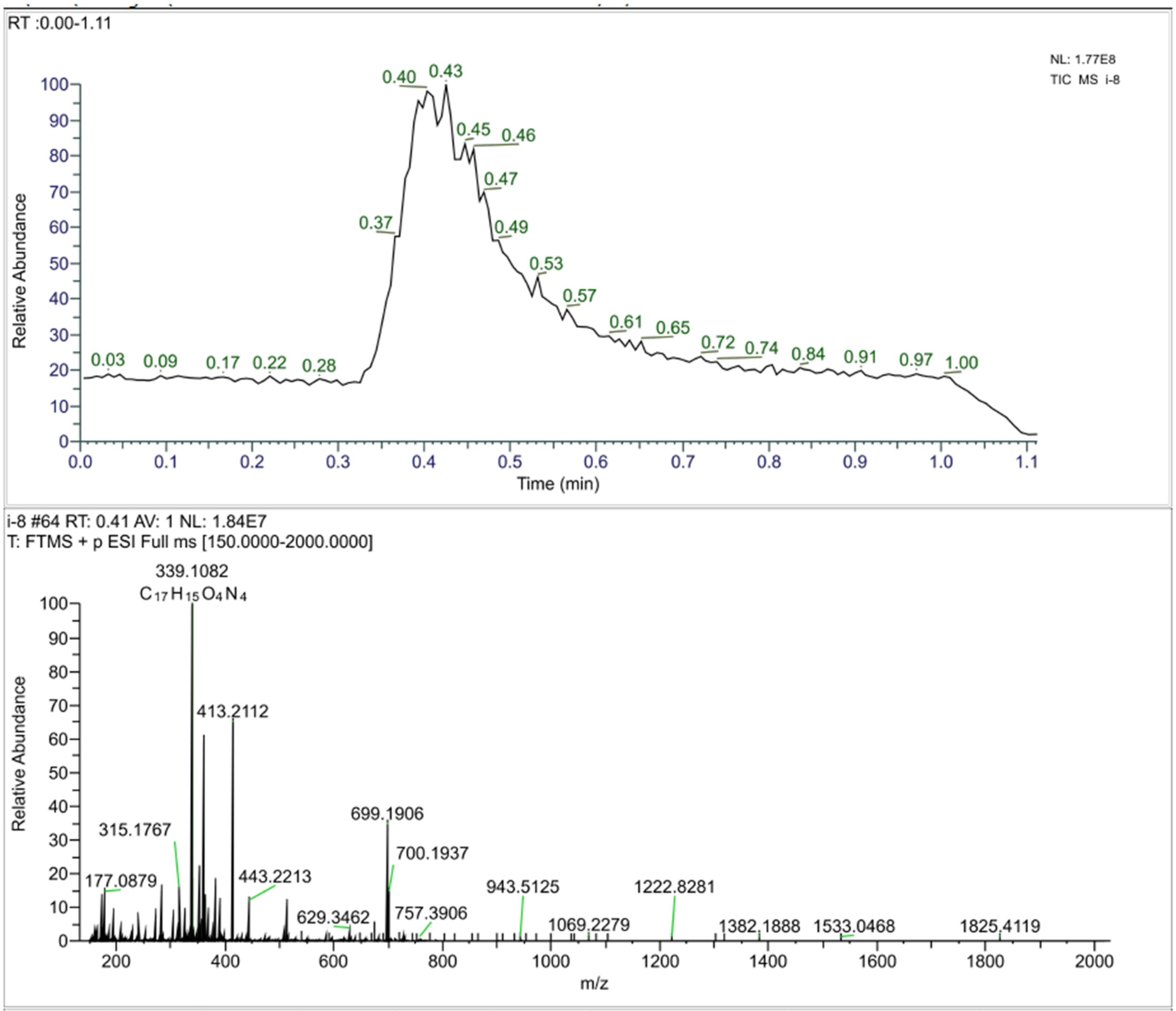

Display F...	S Fit	RDB	Delta [m...	Theo. mass	Rank	Combine...	# Matche...	# Missed...	MS Cov. [...	Pattern C...	MSMS M...
$C_{17}H_{15}O_4N_4$	77.2911708428035	12.50	-0.54	339.10878	1	94.63	4	0	95.6	100	(Collection)

4i

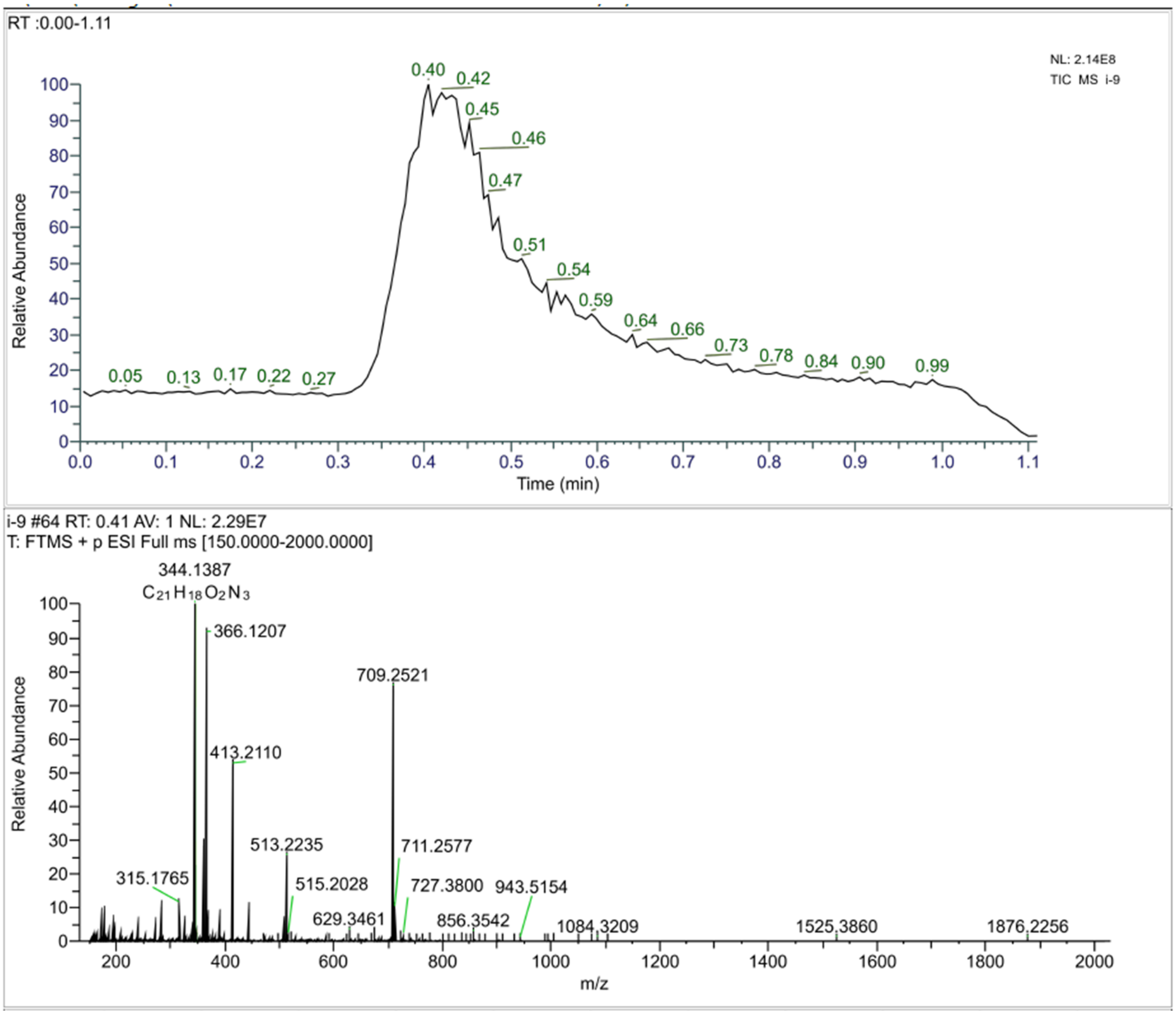

Display F...	S Fit	RDB	Delta [m...	Theo. mass	Rank	Combine...	# Matche...	# Missed...	MS Cov. [...	Pattern C...	MSMS M...
$C_{21}H_{18}O_2N_3$	81.3210395948922	14.50	-0.62	344.13935	1	98.37	4	0	99.32	100	(Collection)

4j

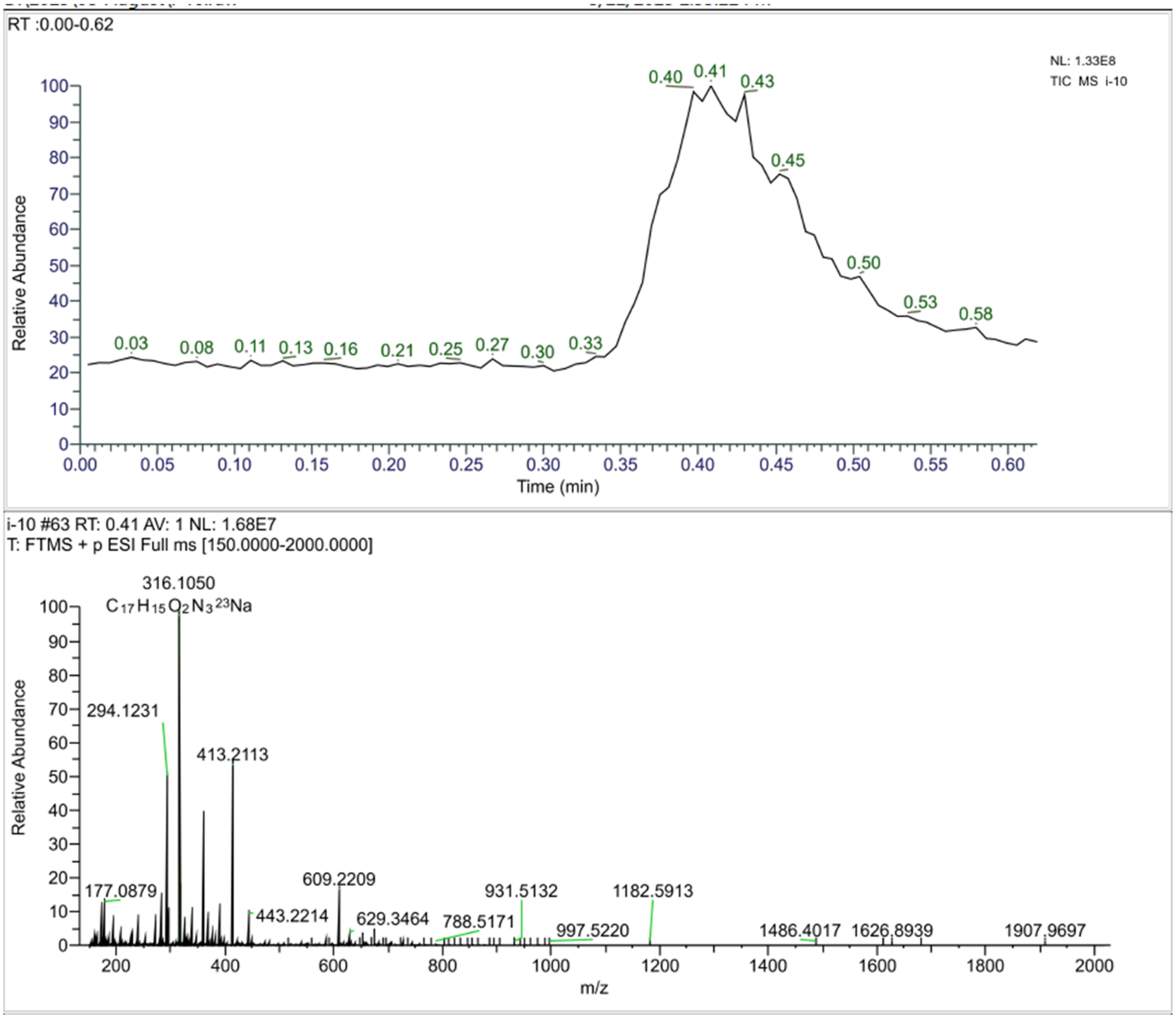

Display F...	S Fit	RDB	Delta [m...	Theo. mass	Rank	Combine...	# Matche...	# Missed...	MS Cov. [...	Pattern C...	MSMS M...
$C_{17}H_{15}O_2N_3{}^{23}Na$	45.6043985643276	11.50	-0.61	316.10565	1	94.85	4	0	97.59	99.88	(Collection)

9. References

(1) M. J. Frisch, G. W. Trucks, H. B. Schlegel, G. E. Scuseria, M. A. Robb, J. R. Cheeseman, G. Scalmani, V. Barone, B. Mennucci, G. A. Petersson, H. Nakatsuji, M. Caricato, X. Li, H. P. Hratchian, A. F. Izmaylov, J. Bloino, G. Zheng, J. L. Sonnenberg, M. Hada, M. Ehara, K. Toyota, R. Fukuda, J. Hasegawa, M. Ishida, T. Nakajima, Y. Honda, O. Kitao, H. Nakai, T. Vreven, J. A. Montgomery Jr., J. E. Peralta, F. Ogliaro, M. Bearpark, J. J. Heyd, E. Brothers, K. N. Kudin, V. N. Staroverov, T. Keith, R. Kobayashi, J. Normand, K. Raghavachari, A. Rendell, J. C. Burant, S. S. Iyengar, J. Tomasi, M. Cossi, N. Rega, J. M. Millam, M. Klene, J. E. Knox, J. B. Cross, V. Bakken, C. Adamo, J. Jaramillo, R. Gomperts, R. E. Stratmann, O. Yazyev, A. J. Austin, R. Cammi, C. Pomelli, J. W. Ochterski, R. L. Martin, K. Morokuma, V. G. Zakrzewski, G. A. Voth, P. Salvador, J. J. Dannenberg, S. Dapprich, A. D. Daniels, Ö. Farkas, J. B. Foresman, J. V. Ortiz, J. Cioslowski and D. J. Fox, *Gaussian 09, Revision D.01*, Gaussian, Inc., Wallingford, CT, 2010.

(2) J.-D. Chai and M. Head-Gordon, Long-range corrected hybrid density functionals with damped atom–atom dispersion corrections. *Phys. Chem. Chem. Phys.*, 2008, **10**, 6615–6620. DOI: 10.1039/b810189b.

(3) F. Weigend and R. Ahlrichs, Balanced basis sets of split valence, triple zeta valence and quadruple zeta valence quality for H to Rn: Design and assessment of accuracy. *Phys. Chem. Chem. Phys.*, 2005, **7**, 3297–3305. DOI: 10.1039/b508541a.

(4) A. B. Nielsen and A. J. Holder, *GaussView 5.0, User's Reference*, Gaussian Inc., Pittsburgh, PA, 2009.
